# Simultaneous 5-HT_1B_R agonist/5-HT_6_R antagonist action as a potential treatment of Parkinson’s disease and its comorbidities

**DOI:** 10.1016/j.jpet.2024.100055

**Published:** 2024-12-09

**Authors:** Paweł Zajdel, Mikołaj Matłoka, Jolanta Konieczny, Tomasz Kos, Josie C. Lammers, Natalie G. Cavalco, Allison A. Clark, Tomasz Lenda, Grzegorz Satała, Vittorio Canale, Katarzyna Grychowska, Martyna Krawczyk, Agnieszka Nikiforuk, Anna Partyka, Magdalena Jastrzębska-Więsek, Klemencja Berghauzen-Maciejewska, Dominika Biała, Monika Janicka, Artur Janusz, Radosław Piast, Krzysztof Mulewski, Damian Smuga, Jerzy Pieczykolan, Maciej Wieczorek, Rafał Moszczyński-Pętkowski, Krzysztof Dubiel, Krystyna Ossowska, Andrzej J. Bojarski, Krzysztof Kamiński, John D. McCorvy, Piotr Popik

**Affiliations:** 1 Faculty of Pharmacy, Jagiellonian University Medical College, Kraków, Poland; 2 R&D Centre, Celon Pharma SA, Kazuń Nowy, Poland; 3 Maj Institute of Pharmacology Polish Academy of Sciences, Kraków, Poland; 4 Department of Cell Biology, Neurobiology and Anatomy, Medical College of Wisconsin, Milwaukee, Wisconsin; 5 Neuroscience Research Center, Medical College of Wisconsin, Milwaukee, Wisconsin; 6 Cancer Center, Medical College of Wisconsin, Milwaukee, Wisconsin

**Keywords:** Cognition, Depression, 5-HT_1B_R agonist, 5-HT_6_R antagonist, Parkinson’s disease

## Abstract

Parkinson’s disease (PD) treatment focuses mainly on the augmentation of dopamine transmission, but to alleviate adverse motor effects accompanying L-DOPA use, additional treatments with serotonergic (5-HT) medications may be considered. We propose a novel concept based on the simultaneous activation of 5-HT_1B_R and 5-HT_6_R blockade as a putative therapeutic option for PD treatment. We have developed PZKKN-94, a dual human 5-HT_1B_R agonist (EC_50_ = 39 nM) and human 5-HT_6_R antagonist (*K*_B_ = 7.7 nM), with selectivity over 43 targets, favorable drug-like properties, and brain penetration. Importantly, PZKKN-94 potency was increased or retained at rat 5-HT_1B_ and 5-HT_6_ orthologs but not at mouse 5-HT_6_. Therefore, PZKKN-94 was tested in 2 rat disease models: haloperidol-induced catalepsy and 6-hydroxydopamine–induced sensorimotor deficits in rats, showing antiparkinsonian-like effects in both. Of note, PZKKN-94 did not affect the therapeutic effects of L-DOPA and attenuated L-DOPA–induced motor fluctuation (“on-off” phenomena) in the stepping and vibrissae tests. PZKKN-94 had no effect on L-DOPA–induced contralateral rotation, suggesting no impact on dopamine-mimetic medication effects. In addition, PZKKN-94 reversed scopolamine-, phencyclidine-, and aged-induced learning deficits in the rat novel object recognition test, increased cognitive flexibility in the attention set-shifting task, and displayed antidepressant-like actions in the forced swim test in rats. Our data suggest that dual-acting 5-HT_1B_R agonists/5-HT_6_R antagonists provide a novel therapeutic approach to alleviate both motor symptoms and accompanying cognitive and depression comorbidities in PD. Our present findings highlight the dual 5-HT_1B_R agonist/5-HT_6_R antagonist strategy to simultaneously spare L-DOPA’s action and alleviate motor fluctuations related to L-DOPA treatment.

**Significance Statement:**

The commonly used L-DOPA–based medications for Parkinson’s disease, though effective in alleviating initial disease states, are limited in long-term use due to the motor (dyskinesia and on-off phenomena) and nonmotor (psychotic-like) side effects. A novel nondopaminergic strategy for treatment of Parkinson’s disease based on simultaneous activation of the 5-HT_1B_ receptor and blockade of the 5-HT_6_ receptor is proposed. The compound PZKKN-94 produces an antiparkinsonian-like effect and attenuates motor fluctuations, preserving the efficacy of L-DOPA. In addition, PZKKN-94 demonstrates procognitive and antidepressant-like properties.

## Graphical abstract

**Figure:**
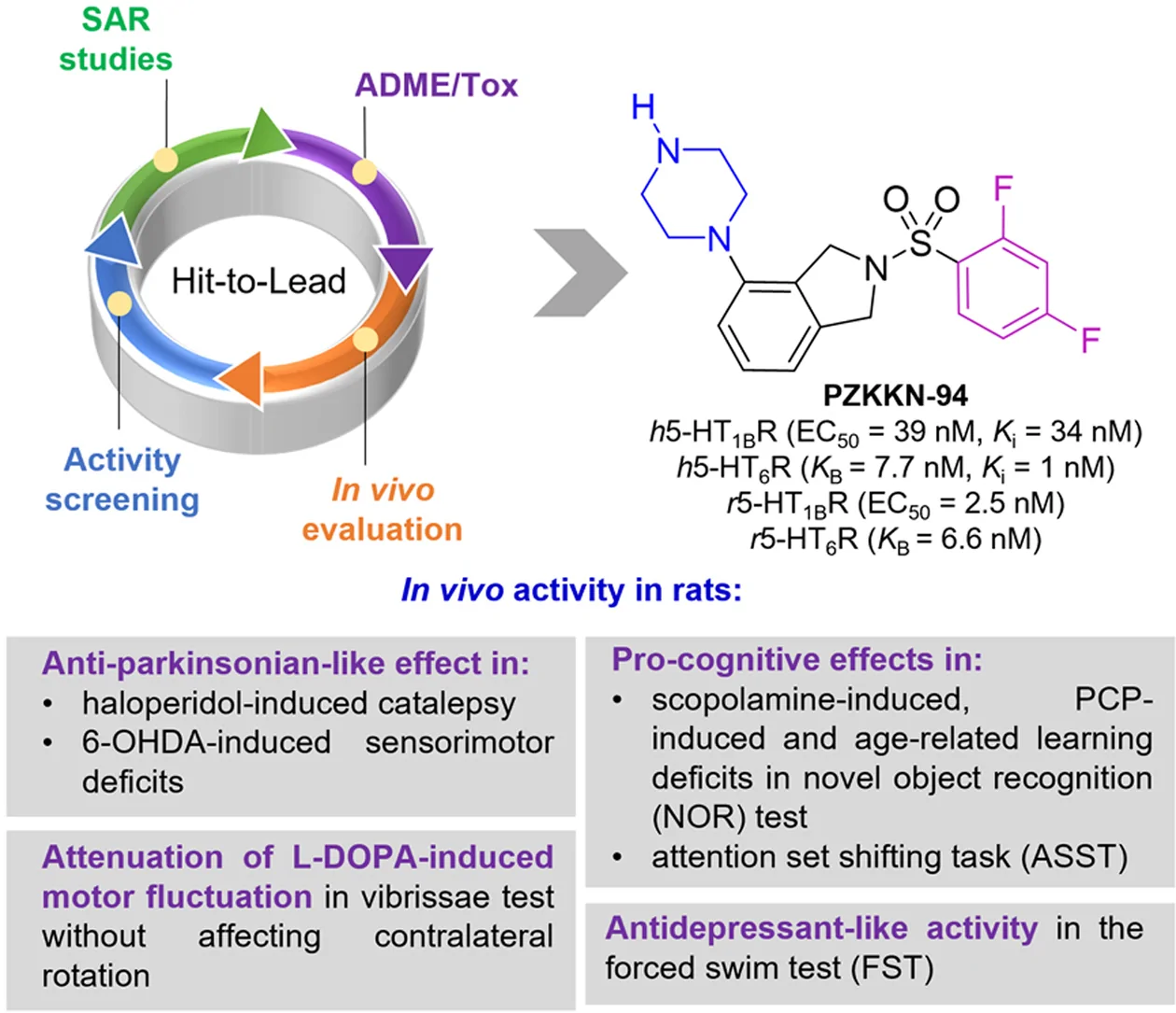

## 1 Introduction

Parkinson’s disease (PD) is a multifactorial disorder characterized by progressive degeneration and death of dopamine neurons in the substantia nigra and by the loss of dopamine in the striatum. It is the second most common neurological disorder in the elderly, with a prevalence of about 1%–2% among patients older than 65 years. The Global Burden of Disease study identified PD as the fastest-growing neurological disorder, with disability-adjusted life years increased by 81% since 2000 (Schiess et al, 2022).

PD motor symptoms, including slowness of movement, muscle rigidity, and tremor, are typically treated with L-DOPA, a dopamine precursor used in combination with peripheral DOPA decarboxylase inhibitors and other agents that stimulate dopamine neurotransmission. While L-DOPA alleviates PD symptoms in the initial stages of the disease, its long-term treatment is burdened with the occurrence of motor (dyskinesias and on-off/wearing phenomena) and nonmotor (psychotic episodes and autonomic system dysfunctions) side effects.

Clinical and preclinical data reveal that adverse effects of L-DOPA treatment and nonmotor symptoms associated with PD (ie, cognitive deficits and depression) are attributed to the serotonergic system (Carta and Tronci, 2014). For instance, in the early stages of PD, striatal serotonin afferents compensate for the loss of dopaminergic neurons by converting and releasing dopamine derived from exogenous L-DOPA (Carta et al, 2007). However, with disease progression, serotonin neurons lose a feedback mechanism to regulate synaptic dopamine peaks in the brain, thereby contributing to the prodyskinetic effects of L-DOPA (Cenci, 2007).

In this regard, stimulation of serotonin type 1 receptors (5-HT_1_R) that negatively regulate serotonergic neurons has been proposed to manage L-DOPA–induced dyskinesia (Cenci et al, 2022). Studies demonstrated critical localization of 5-HT_1B_R on serotonin terminals in the striatum and striatal GABAergic output pathways (Bruinvels et al, 1994). Importantly, 5-HT_1B_R agonists diminish L-DOPA–induced dyskinesia (Zhang et al, 2008) and exert antiparkinsonian-like effects in the rat models of PD (Chadha et al, 2000; Wang et al, 2013). However, the clinical studies of 5-HT_1A_R and/or 5-HT_1B_R were hampered due to worsening of parkinsonian motor symptoms (Kannari et al, 2002) or due to a decline in L-DOPA therapeutic efficacy (Bézard et al, 2013). In this work, we focused on a novel concept, combining blockade of 5-HT_6_R with activation of 5-HT_1B_R. This is because 5-HT_6_R antagonists in preclinical studies display therapeutic potential in the treatment of PD, purportedly due to the localization of 5-HT_6_Rs in areas involved in motor control, including the striatum (Marin et al, 2020; Barnes et al, 2021). In this regard, Tatara et al, (2012) and Ohno et al, (2015) demonstrated that blockade of 5-HT_6_Rs alleviated haloperidol-induced bradykinesia and catalepsy. As compounds inhibiting 5-HT_6_Rs also display procognitive (Nikiforuk, 2014; Berthoux et al, 2020; Vanda et al, 2021) and antidepressant-like effects (Zajdel et al, 2016; Partyka et al, 2019), here we employed a wide battery of behavioral tests to test for such effects.

We propose that compounds simultaneously stimulating 5-HT_1B_Rs and inhibiting 5-HT_6_Rs could be a novel strategy for the management of PD symptoms. For the first time, we describe the development of the first-in-class dual-acting serotonergic compound PZKKN-94 (2-[(2,4-difluorophenyl)sulfonyl]-4-(piperazin-1-yl)isoindoline hydrochloride) and its in vitro and in vivo effects.

## 2 Materials and methods

### 2.1 In vitro biological evaluation of compound PZKKN-94

Details disclosing synthesis and physicochemical properties of the compound PZKKN-94 (Supplemental Figs. 1 and 2) and cAMP functional data describing the assessment of its activity at human (h)5-HT_1B_R and h5-HT_6_R were according to the method previously disclosed by Zajdel et al, (2021). G protein dissociation activity as measured by bioluminescence resonance energy transfer (BRET) at h5-HT_1B_R, h5-HT_6_R, and h5-HT_1F_R was according to the method previously disclosed (Lewis et al, 2023), and rat and mouse (m)5-HT_1B_R and 5-HT_6_R were cloned into pcDNA3.1 using Gibson Assembly from GeneBlocks synthesized by Integrated DNA Technologies. Details of the G protein dissociation assay are presented in the Supplemental Material. Selectivity panel, P-glycoprotein efflux pump inhibition, protein plasma binding, cytochrome P450 inhibition, and AMES test outcome are presented in the Supplemental Material.

### 2.2 Animals

Male Wistar rats (220–250 g) were used for pharmacokinetic assessment, the forced swim test (FST), and the open field test. Male Wistar Han rats (270–300 g) were used for the stepping, vibrissae, and catalepsy tests. Male Sprague-Dawley rats (170–200 g) were used for the scopolamine and phencyclidine (PCP) novel object recognition (NOR) studies and attention set-shifting task (ASST) study. Young (10 weeks old) and aged (19 months old) male Wistar Han rats were used for the natural forgetting NOR study.

### 2.3 Haloperidol-induced catalepsy

Catalepsy was assessed by using a wooden block (9 cm high and 3 cm wide). Both forepaws of a rat were put on a block, and descent latency was measured 3 times at most for up to 300 seconds each time, and the highest score was taken for further analysis (Konieczny and Lenda, 2013). The endpoint of catalepsy was taken as complete forepaw withdrawal from the block. Catalepsy was induced by haloperidol (0.25 mg/kg). Compound PZKKN-94 (1 and 3 mg/kg) was administered 15 minutes before haloperidol. Testing was performed 60 minutes after haloperidol administration.

#### 2.3.1 Induction of 6-hydroxydopamine lesions

Rats were deeply anesthetized with a mixture of ketamine (70 mg/kg) and xylazine (6 mg/kg) administered intraperitoneally in a volume of 1 mL/kg and placed in a stereotaxic apparatus (David Kopf Instrument). 6-hydroxydopamine (6-OHDA) (8 *μ*g/4 *μ*L, expressed as the free base) was injected into the left medial forebrain bundle at a flow rate of 0.5 *μ*L/min using a Hamilton microsyringe connected via polyethylene tubing to the stainless steel cannula (0.28 mm o.d.), as described previously (Konieczny et al, 2015). The stereotaxic coordinates relative to the bregma were as follows: −2.8 AP, +1.8 ML, and −8.6 DV (Paxinos and Watson, 1986). The cannula was left in place for a further 5 minutes for complete diffusion of the toxin and then was slowly retracted. The solution of 6-OHDA was freshly prepared before surgery, kept on ice, and protected from exposure to light prior to use.

#### 2.3.2 Procedure for 6-OHDA lesioned rats

Evaluation of the antiparkinsonian properties of PZKKN-94 in rats unilaterally treated with 6-OHDA followed the experimental schedule shown in Supplemental Fig. 3. Briefly, after 5-day handling, “prelesion” behavioral testing (the stepping, vibrissae, and catalepsy tests) was performed. Next, rats were lesioned with 6-OHDA. Two weeks after surgery, the lesion degree was verified using an apomorphine (0.25 mg/kg)-induced rotation test (Konieczny et al, 2014). Rats that passed testing criteria (>100 rotations/h; 75% of the initial number of animals) were allocated to individual groups and balanced with respect to their rotational score. Three weeks after lesion, the “postlesion” behavioral testing was conducted to assess motor deficits produced by 6-OHDA. Next day, rats were administered acutely with the compound PZKKN-94 (0, 1, 3, and 10 mg/kg) and tested again, starting 90 minutes following PZKKN-94 administration. Four weeks after lesion, combined administration of PZKKN-94 and L-DOPA (6 mg/kg) given with a peripheral DOPA decarboxylase inhibitor benserazide (6 mg/kg; administered 30 minutes prior to L-DOPA) was started and continued for 16 days. Behavioral tests were carried out 45 minutes after L-DOPA (90 minutes after compound PZKKN-94 administration), 3 times during the combined administration: after the first, before (OFF phase), and after (ON phase) the last (16th) L-DOPA dose. Additionally, rats were tested for rotational behavior 3 times during combined drug administration: after the 2nd, 8th, and 15th L-DOPA dose. Measurements started immediately after L-DOPA administration (45 minutes after compound PZKKN-94) and lasted 90 minutes. One week after completion of the study, rats were sacrificed by decapitation, and their left and right striata were dissected to verify the level of dopamine using the high-performance liquid chromatography method (Supplemental Methods, section 3.4.2).

#### 2.3.3 Stepping test

The stepping test, indicative of gait problems and bradykinesia in PD, was performed according to Olsson et al, (1995). The number of adjusting steps was counted for both forelimbs, and the sequence of testing was the left limb in the forehand and backhand direction, followed by the right limb in the same manner (Lenda et al, 2021). The use of the impaired (contralateral) forelimb (IF) relative to the total number of forelimb use (IF + unimpaired forelimb) was calculated according to the following formula:

(1) $$IF\left(\%\right)=\left(\frac{IF}{IF+UF}\right)\cdot 100\%$$

#### 2.3.4 Vibrissae test

The vibrissae test, considered analogous to cue-induced movement initiation training for patients with PD, was carried out according to Schallert et al, (2000). Each session consisted of 2 independent tests of each forelimb (2 × 10 trials). The use of the IF was calculated according to formula (1).

#### 2.3.5 NOR test

The NOR test in rodents has been used as an ethologically relevant measure of cognitive effects of novel compounds (Ennaceur and Delacour, 1988). This test is based on the spontaneous exploration of 2 objects at the acquisition and retention trials separated by some time. A lower exploration of the familiar object at the retention trial signifies a better memory of the object (Potasiewicz et al, 2020; Zajdel et al, 2021). In 3 sets of experiments, we investigated the cognitive effects of PZKKN-94 in the conditions in which learning was compromised by the administration of anticholinergic scopolamine, *N*-methyl-D-aspartate (NMDA) receptor antagonist PCP, and in aged rats. For detailed methodology, see Supplemental Methods.

#### 2.3.6 ASST

The ASST assesses cognitive flexibility, that is, the ability to modify behavior in response to the altering relevance of stimuli. In this test, rats must select a bowl containing a food reward based on the ability to discriminate the odors or the media covering the bait (Birrell and Brown, 2000). The ASST requires rats to initially learn a rule and form an attentional “set” within the same stimulus dimensions. At the extradimensional (ED) shift stage, the essential phase of the task, animals must switch their attention to a previously irrelevant stimulus dimension and, for example, discriminate between the odors and not between the media covering the bait. The animal’s performance at the ED stage is considered an index of cognitive flexibility, and its facilitation may serve as an index of procognitive action of tested compounds (Denis Goetghebeur and Dias, 2014; Nikiforuk et al, 2020). For detailed methodology, see Supplemental Methods.

#### 2.3.7 FST

The test was performed as described by Porsolt (Porsolt et al, 1978) with the modifications proposed by Detke (Detke et al, 1995). On the first day of the experiment, each animal was placed in Plexiglas cylinders (40 cm high and 18 cm in diameter) containing 30 cm of water at a temperature of 23–25 °C for 15 minutes. On removal from the water, the rat was placed for 30 minutes in a Plexiglas box under a 60 W bulb to dry. Twenty-four hours later, the rat was placed again in the cylinder, and the total duration of immobility, swimming, and climbing was recorded during the 5-minute test period. The swimming behavior entailed active swimming motions, for example, horizontal movement around the cylinder. Climbing activity included upward-directed movements of the forepaws of the rat along the side of the swimming chamber. The immobility was assigned when the rat did not show additional activity other than that necessary to keep its head above the water. Fresh water was used for each animal. The test was performed by 2 female experimenters.

### 2.4 Data and statistical analysis

Statistical analyses were performed using Statistica v.13.3 (StatSoft) and GraphPad Prism v.9.1.0 software (GraphPad). All data were presented as the mean ± SEM. Before analysis, the data were checked for normality using the Shapiro-Wilk or D'Agostino-Pearson tests.

#### 2.4.1 Stepping test, vibrissae test, catalepsy, and rotation

Statistical significance in parametrical data was estimated by the paired Student’s *t* test or one-way ANOVA for repeated measures, followed by the Duncan post hoc test. For data that did not pass the normality test, the Wilcoxon signed-rank test or the Friedman ANOVA test was used.

#### 2.4.2 NOR test

Discrimination index (DI) data were analyzed using one-, two- or three-way ANOVAs. Newman-Keuls post hoc test was used in the evaluation of datasets involving repeated and/or mixed design and Tukey’s Honest Significant Difference (HSD) or a priori planned contrasts analyses as the post hoc tests were used in evaluation of datasets involving between-subjects design. The choice of a post hoc test was based on Cardinal and Aitken (2005).

#### 2.4.3 ASST

The number of trials required to achieve the criterion of 6 consecutive correct responses (ie, trials to criterion) was recorded for each rat and each discrimination phase of the ASST (Drop et al, 2021). The data were analyzed using a two-way ANOVA, with PZKKN-94 treatment as a between-subject factor and the discrimination phase as a repeated measure. Tukey’s HSD was used as the post hoc test.

#### 2.4.4 FST

All data are presented as mean ± SEM. The statistical significance of the results was evaluated using a one-way ANOVA, followed by the Bonferroni comparison test.

## 3 Results

### 3.1 PZKKN-94 displays a distinct 5-HT_1B_/5-HT_6_ receptor profile and favorable in vitro ADME/Tox profile

The compound PZKKN-94 was identified from a library of arylsulfonyl derivatives of isoindolines substituted with different alkyl amines using a pharmacophore-based design approach and merging the structural motifs responsible for intrinsic activity at 5-HT_1B_R and antagonism at 5-HT_6_R (Fig. 1) (Kamiński et al, 2019). A series of high-value isoindoline derivatives was optimized in a sustainable manner to improve metabolic stability, blood-brain barrier permeability, and safety. We finally identified di-fluoro substituted phenyl ring in combination with piperazine, as an amine fragment, as the most preferential substructure in terms of potency at both targets and metabolic stability.

**Fig. 1:**
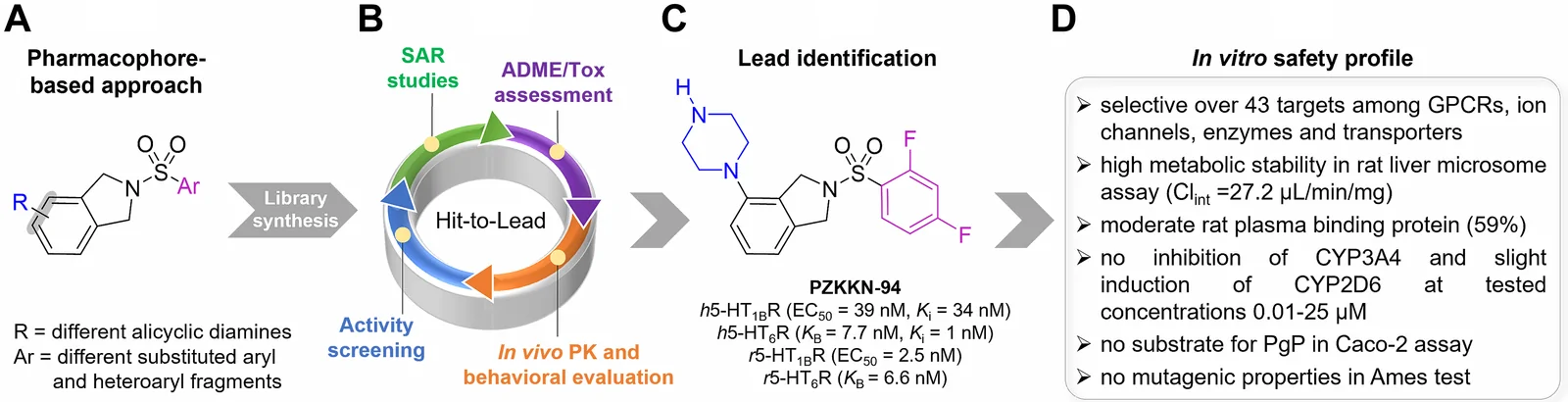
PZKKN-94 is the first-in-class dual-acting 5-HT_1B_R agonist/5-HT_6_R antagonist. (A) The structure of the isoindoline core functionalized with different alicyclic diamine (R) and different substituted aryl and heteroaryl fragment (Ar) diversity points, which was used as a template for chemical modification using the structural properties of known active agonists of 5-HT_1B_R and antagonists of 5-HT_6_R (pharmacophore-based approach). The designed compounds’ library was synthesized using methods shown in the Supplemental Material. (B) Diagram of an iterative approach designed to achieve selectivity of synthesized compounds’ library members, their intestinal and blood-brain barrier permeability, metabolism, and safety using in vitro methods and in vivo pharmacokinetics, followed with preliminary behavioral evaluation. (C) Structure and potency of PZKKN-94 at 5-HT_1B_R and 5-HT_6_R. (D) Summary on selectivity (Supplemental Table 1) and in vitro adsorption, distribution, metabolism, excretion, and toxicology (ADME/Tox) properties for PZKKN-94. Cl_int_, intrinsic clearance; GPCR, G-protein coupled receptor; PgP, P-glycoprotein; PK, pharmacokinetics; SAR, structure-activity relationships.

PZKKN-94 showed a desired dual affinity for h5-HT_1B_R and h5-HT_6_R (*K*_i_ [5-HT_1B_R] = 34 nM and *K*_i_ [5-HT_6_R] = 1 nM). The high affinity of PZKKN-94 was confirmed in functional assays measuring G protein dissociation in BRET assays (Lewis et al, 2023), where PZKKN-94 demonstrated potent agonist activity at h5-HT_1B_R (EC_50_ = 39.5 nM, maximum effect [E_MAX_] = 88.1% of 5-HT response; Fig. 2A), also displaying weak agonist potency at h5-HT_1F_R (EC_50_ = 1490 nM, E_MAX_ = 72.0%; Supplemental Fig. 4A), resulting in greater than 30-fold 5-HT_1B_R selectivity over 5-HT_1F_R. 5-HT_1B_R activity was confirmed in cAMP inhibition experiments where PZKKN-94 demonstrated potent agonist activity in this assay (EC_50_ = 52.1 nM, E_MAX_ = 97.9% of 5-HT response; Fig. 2B). 5-HT_6_R antagonist activity by PZKKN-94 to block 5-HT stimulation was demonstrated in G protein dissociation assays measured by BRET (*K*_B_ = 7.7 nM; Fig. 2C). Similarly, the antagonist activity of PZKKN-94 was also demonstrated in cAMP accumulation assays to block 5-carboxamidotryptamine 5-HT_6_R stimulation (*K*_B_ = 1.2 nM; Fig. 2D). Because of known species differences between human and rodent orthologs of 5-HT_1B_R that can affect translation to animal models (Oksenberg et al, 1992), we chose to measure the activity of PZKKN-94 at both mouse (m) and rat (r)5-HT_1B_R. Comparison of PZKKN-94 at m5-HT_1B_R (EC_50_ = 1.2 nM, E_MAX_ = 104.6%) and r5-HT_1B_R (EC_50_ = 2.5 nM, E_MAX_ = 93.2%) versus h5-HT_1B_R reveals that PZKKN-94 is approximately 10-fold more potent to activate rodent orthologs of 5-HT_1B_R (Fig. 2E), despite similar 5-HT potency between human and rodent orthologs (Supplemental Fig. 4B). Next, we also compared species differences between human and rodent 5-HT_6_R in G protein dissociation assays by BRET and found that rat but not m5-HT_6_R antagonist affinity was preserved relative to human orthologs (r5-HT_6_R *K*_B_ = 6.6 nM; m5-HT_6_R *K*_B_ = 101 nM; Fig. 2F). Finally, there was no difference in potency for 5-HT to activate 5-HT_6_R at human versus rodent orthologs (Supplemental Fig. 4C), and no agonist activity was detected at r5-HT_6_R orthologs versus human in the Gs protein dissociation BRET assay (Supplemental Fig. 4D).

**Fig. 2:**
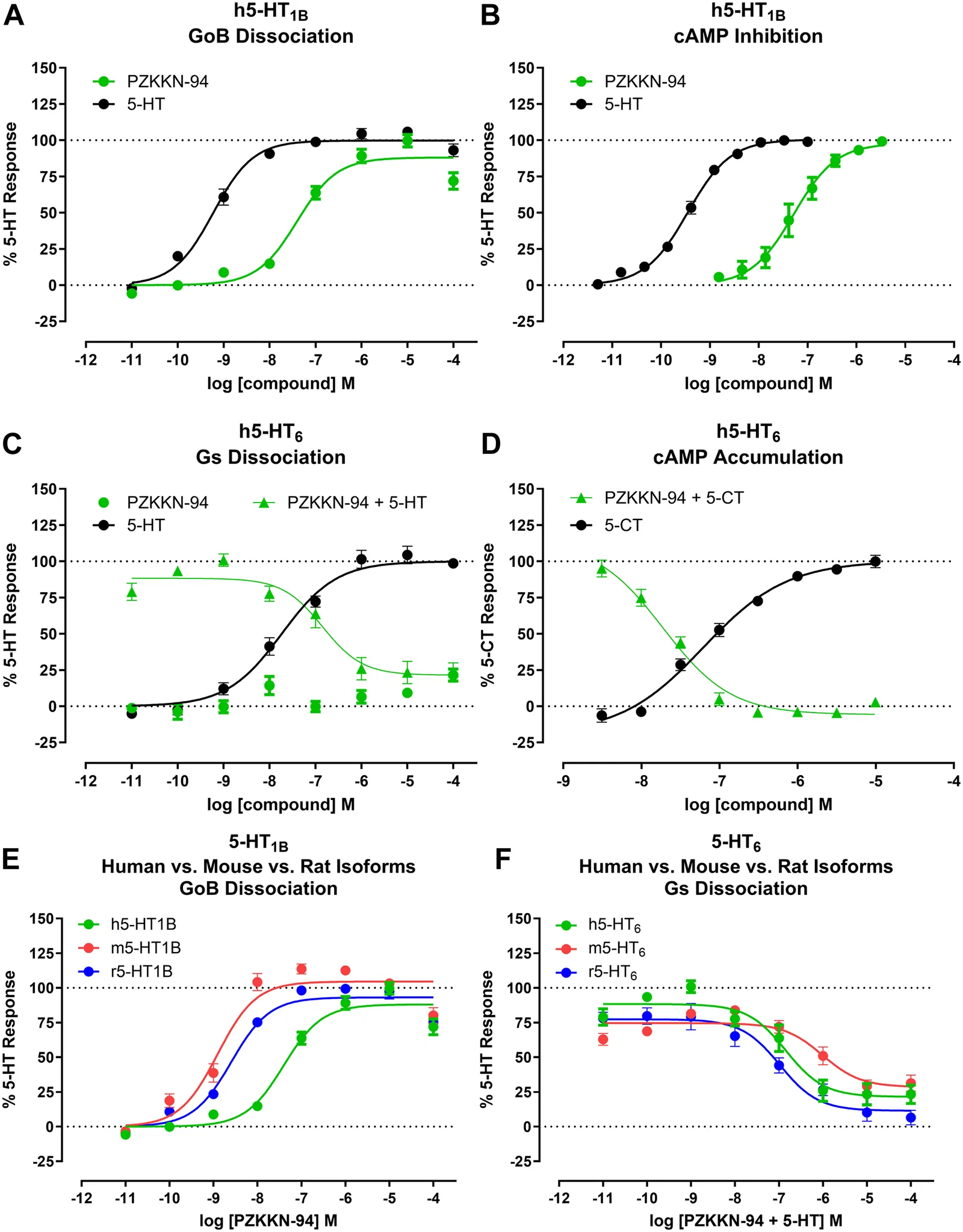
PZKKN-94 behaves as a 5-HT_1B_R agonist/5-HT_6_R antagonist in the BRET and cAMP assays. (A) h5-HT_1B_R G(oB) protein dissociation activity as measured by BRET. PZKKN-94 (green; EC_50_ = 39.5 nM, E_MAX_ = 88.1% of 5-HT response) compared with 5-HT (black; EC_50_ = 0.59 nM). Data represent the mean ± SEM from 5 independent experiments (*n* = 5) performed in duplicate. (B) 5-HT_1B_R cAMP inhibition activity for 5-HT (black; EC_50_ = 0.35 nM) and PZKKN-94 (green; EC_50_ = 52.1 nM, E_MAX_ = 97.9%). Data are presented as the mean ± SEM∗ of the values obtained in 5 independent experiments. ∗SEM < 20%. (C) h5-HT_6_R G protein dissociation activity as measured by BRET. PZKKN-94 as an agonist (green circles; no activity), PZKKN in competition with 320 nM 5-HT (green triangles; *K*_B_ = 7.7 nM), and 5-HT (black; EC_50_ = 18.4 nM). Data represent the mean ± SEM from 3 independent experiments (*n* = 3) performed in duplicate. (D) 5-HT_6_R cAMP accumulation activity of 5-carboxamidotryptamine (5-CT; black; EC_50_ = 62.2 nM) and PZKKN-94 (green; *K*_B_ = 1.2 nM) in competition with 5-CT EC_80_ concentration. Data are presented as the mean ± SEM∗ of the values obtained in 5 independent experiments. ∗SEM < 20%. (E) Comparison of PZKKN-94 at h5-HT_1B_R (green; EC_50_ = 39.5 nM, E_MAX_ = 88.1%), m5-HT_1B_R (red; EC_50_ = 1.2 nM, E_MAX_ = 104.6%), and r5-HT_1B_R (blue; EC_50_ = 2.5 nM, E_MAX_ = 93.2%), as measured by G protein dissociation activity by BRET. Data represent the mean ± SEM from 5 independent experiments (*n* = 5) performed in duplicate. (F) Comparison of antagonist activity of PZKKN-94 in competition with 320 nM 5-HT at h5-HT_6_R (green; *K*_B_ = 7.7 nM), m5-HT_6_R (red; *K*_B_ = 101 nM), and r5-HT_6_R (blue; *K*_B_ = 6.6 nM), as measured by Gs protein dissociation activity by BRET. Data represent the mean ± SEM from 3 independent experiments (*n* = 3) performed in duplicate.

Next, we chose to test PZKKN-94 in a selectivity screen against 43 off-targets, including selected G-protein coupled receptors, enzymes, nuclear receptors, transporters, and ion channels, which revealed an initial result of PZKKN-94 binding to 7 targets (% inhibition at 10 *μ*M higher than 70%; Supplemental Table 1). However, secondary dose-response testing revealed potency values at these targets in the micromolar range (Supplemental Table 2), which, given PZKKN-94’s ADME profile, these potencies may not be considered relevant to the pharmacological activity of PZKKN-94.

Importantly, compound PZKKN-94 showed several promising drug-like properties assessed using in vitro methods. It displayed favorable metabolic stability in rat liver microsomes assay with intrinsic clearance of 27.2 mL/min/mg), moderate plasma protein binding (59%; Supplemental Fig. 5), low inhibitory activity against cytochrome P450 (Supplemental Fig. 6), suggestive of the low propensity of drug-drug interaction, high cell membrane penetration, and no P-glycoprotein substrate activity in a Caco2 permeability assay (Supplemental Table 3), indicating favorable intestinal permeability and no active efflux liability. In a genotoxicity study, PZKKN-94 showed no mutagenic potential in the AMES test (Supplemental Table 4).

### 3.2 PZKKN-94 penetrates the blood-brain barrier and has a good oral bioavailability

Compound PZKKN-94 underwent pharmacokinetic evaluation before advancing to behavioral testing. Rats received PZKKN-94 in a dose of either 3 mg/kg i.v. or 10 mg/kg orally. Pharmacokinetic analyses (Table 1) indicated that compound PZKKN-94 had a good oral bioavailability of 31% with very good exposure to the brain as evidenced by a brain concentration (*C*_max_) of 172 ng/g reached by 1 hour (time of maximum concentration) after per os administration (brain-to-plasma ratio, 2). The compound exhibited half-life of 2 hours after intravenous administration.

**Table 1:** Pharmacokinetic parameters for compound PZKKN-94 in rats. Data are presented as the means ± SEM

Parameter	Per os^a^		Intravenous^b^
	Plasma	Brain	Plasma
C_0_ (ng/mL)	NA	NA	404 ± 36
*C*_max_ (ng/mL)	140 ± 53	172 ± 37	110 ± 7
T_max_ (h)	0.25	1	NA
AUC_(0–t)_ (ng ∗ min/mL)	22,696 ± 2446	42,313 ± 3821	24,834 ± 706
t_1/2_ (h)	NA	NA	1.99 ± 0.24
Cl (mL/min/kg)	NA	NA	112.9
V_d_ (L/kg)	NA	NA	16.2
F (%)	31	NA	NA
B/P	NA	2	NA

AUC, area under the curve; B/P, blood-to-plasma ratio; C_0_, predicted concentration at time 0; Cl, clearance; F, bioavailability; NA, not available; t_1/2_, half-life of elimination; T_max_, time of maximum concentration; V_d_, volume of distribution.

a PZKKN-94 in a dose of 10 mg/kg was administered via oral gavage (*n* = 5, destructive sampling).

b PZKKN-94 in a dose of 3 mg/kg was administered intravenously (*n* = 5, serial sampling).

### 3.3 PZKKN-94 produces an antiparkinsonian-like effect in the haloperidol-induced catalepsy

Haloperidol-induced catalepsy is thought to be mediated primarily through inhibition of dopaminergic D_2_ receptors in the striatum and is often used as a rodent model for the study of motor impairments (akinesia and rigidity) and for the rapid screening of potential antiparkinsonian compounds (Waku et al, 2021). Haloperidol (0.25 mg/kg) produced strong catalepsy measured 60 minutes after drug injection, which was significantly attenuated by the higher dose of compound PZKKN-94 (3 mg/kg) (decrease by 38.81%) (Duncan test following a significant treatment effect: *F*[2,33] = 3.80, *P* < .05) (Supplemental Fig. 7). Furthermore, PZKKN-94 had no influence on the anticataleptic effect of L-DOPA after combined treatment in 6-OHDA hemiparkinsonian rats (Supplemental Fig. 8) and did not impact contralateral rotation induced by L-DOPA in the 6-OHDA model following acute and prolonged treatment (Supplemental Fig. 9). Table 2 provides the summary of findings.

**Table 2:** Summary of in vivo results for the compound PZKKN-94

Test	Purpose/Measure	PZKKN-94 Dose (mg/kg), Route of Administration, and Significant Effect	Rat Strain	Figure	Notes
Haloperidol-induced catalepsy	To examine antiparkinsonian-like effects in haloperidol-induced catalepsy model	1, 3^a^ (i.p.)	Wistar Han	Supplemental Fig. 7	
Stepping	To examine antiparkinsonian-like effects in the 6-OHDA hemiparkinsonian model following acute administration as well as combined acute and prolonged treatment with L-DOPA	1^a^, 3^a^, 10^a^ (per os)	Wistar Han	4, A and B	After combined treatment, no influence on the therapeutic effect of L-DOPA
Vibrissae	To examine anti–parkinsonian-like effects in the 6-OHDA hemiparkinsonian model following acute administration as well as combined acute and prolonged treatment with L-DOPA	1^a^, 3^a^, 10^a^ (per os); combined acute treatment 3^a^, 10^a^ (per os); combined prolonged treatment – OFF phase	Wistar Han	4, C and D	No effect on vibrissae-elicited forelimb placing after acute administration After combined acute and prolonged treatment with L-DOPA (OFF phase), augmentation of L-DOPA effect
Catalepsy produced by 6-OHDA lesion	To examine the effect on akinesia in the 6-OHDA hemiparkinsonian model following acute administration as well as combined acute and prolonged treatment with L-DOPA	1, 3, 10 (per os)	Wistar Han	Supplemental Fig. 8	No effect on catalepsy produced by 6-OHDA after acute administration After combined treatment no influence on the therapeutic effect of L-DOPA
Rotational	To examine the influence on contralateral rotation induced by L-DOPA in the 6-OHDA hemiparkinsonian model following acute and prolonged treatment	1, 3, 10 (per os)	Wistar Han	Supplemental Fig. 9	No influence on the therapeutic effect of L-DOPA
NOR: PCP-induced deficit	To examine cognitive effects in PCP-induced deficit	0.3, 1^a^, 3^a^ (per os)	Sprague-Dawley	5A	
NOR: scopolamine-induced deficit	To examine cognitive effects in scopolamine-induced deficit following acute and prolonged treatment	0.3^a^ (per os)	Sprague-Dawley	5B	No tolerance for the behavioral effect following the 14-d treatment
NOR in young and aged rats	To establish age-related deficits in object recognition	None: young (10 wk) and old (19 mo) rats show learning deficits 24 and 48 h after the acquisition trial and that at ITI of 24 h is more severe in aged rats	Wistar Han	5C	Aged rats of only this strain were available
NOR in young and aged rats 24 h after the acquisition trial	To examine cognitive effects in aged rats	0.3^a^, 1^a^ (per os) in aged animals	Wistar Han	5D	Donepezil used as the “positive” control improved learning in aged rats only
ASST	To examine the effects on cognitive flexibility	0.3, 1^a^, 3 (per os)	Sprague-Dawley	6	
FST	To examine antidepressant-like effects	0.1^a^, 0.3^a^, 1 (i.p.)	Wistar	Supplemental Fig. 11	Wistar rat is 1 of the 2 rat strains commonly used in FST (Bogdanova et al, 2013)
Spontaneous activity	To examine side effects possibly confounding forced swim readout	0.1, 0.3 (i.p.)	Wistar	Supplemental Table 5	No impact on animal spontaneous activity

ITI, intertrial interval.

a Significant effect.

### 3.4 PZKKN-94 decreases 6-OHDA–induced motor deficits in the stepping test

Quantitative evaluation of the 6-OHDA–produced motor deficits in rats is presented in Supplemental Fig. 10. After acute administration, all doses of compound PZKKN-94 (1, 3, and 10 mg/kg) dose-dependently improved the stepping test performance with the IF (Duncan test following one-way ANOVA for repeated measures, treatment effect: *F*[3,28] = 5.21, *P* < .05; time effect: *F*[1,28] = 36.90, *P* < .05; time × treatment interaction: *F*[3,28] = 5.67, *P* < .05) (Fig. 3A). After combined treatment, the first dose of L-DOPA (6 mg/kg) completely restored the symmetry in the use of forelimbs in the stepping test (Fig. 3B). The beneficial L-DOPA effect persisted after a 2-week treatment and was not affected by PZKKN-94 (one-way ANOVA for repeated measures: treatment effect *F*[3,27] = 1.11, *P* > .05; time effect *F*[3,81] = 222.98, *P* < .05; time × treatment interaction: *F*[9,81] = 2.65, *P* = .06). In the OFF phase, the therapeutic effect of L-DOPA was weaker but still significant compared with the same group of lesioned rats before treatment. However, the beneficial effect was enhanced in all groups receiving combined treatment compared with the rats treated with L-DOPA alone.

**Fig. 3:**
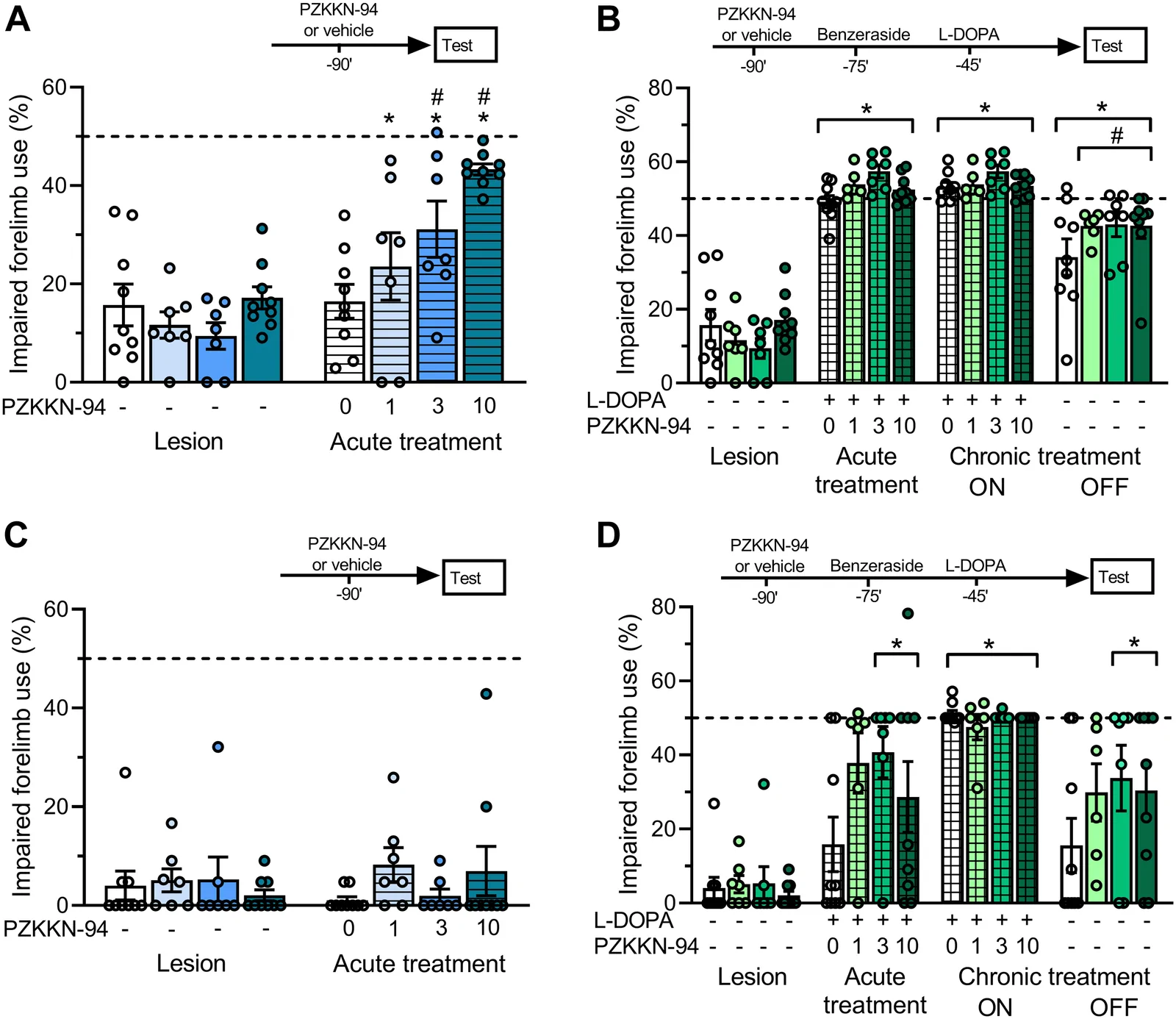
The effect of PZKKN-94 and L-DOPA on the performance of the stepping test and the vibrissae-elicited forelimb placing test in unilaterally 6-hydroxydopamine (6-OHDA)–lesioned rats. Results represent the mean percent of IF use ± SEM. (A) Acute PZKKN-94 increases IF use in rats with 6-OHDA lesions, suggesting an anti–parkinsonian-like effect. *n* = 7–9 rats per group. ^∗^*P* < .05 versus Lesion; ^#^*P* < .05 versus vehicle. (B) In the lesioned rats, L-DOPA restores IF use, and neither acute nor chronic PZKKN-94 treatment affects L-DOPA therapeutic action. In the OFF phase, combined treatment improves the test performance compared with the L-DOPA group, suggesting that PZKKN-94 may attenuate L-DOPA–induced motor fluctuations. *n* = 6–9 rats per group. ^∗^*P* < .05 versus Lesion; ^#^*P* < .05 versus vehicle + L-DOPA 6 mg/kg. (C) Acute PZKKN-94 does not affect IF use in rats with 6-OHDA lesions. *n* = 7–9 rats per group. (D) Combined acute treatment but not L-DOPA alone attenuates IF use compared with the Lesion group. Combined chronic treatment improves the test performance in the OFF phase compared with the Lesion group, suggesting that PZKKN-94 may attenuate L-DOPA–induced motor fluctuations. *n* = 6–9 rats per group. ^∗^*P* < .05 versus Lesion.

### 3.5 PZKKN-94 augments the efficacy of L-DOPA in the vibrissae test

In contrast to the stepping test, neither acute PZKKN-94 (Friedman ANOVA test: c^2^ [32, *df* 1] = 0.07, *P* > .05) (Fig. 3C) nor acute L-DOPA (Friedman ANOVA test: c^2^ [9, *df* 1] = 2.67, *P* > .05) (Fig. 3D) affected the vibrissae-elicited placement of the IF compared with the same rats before treatment. However, combined acute treatment with L-DOPA and 2 higher doses of PZKKN-94 (3 and 10 mg/kg) reduced deficits in the impaired limb compared with the same groups of untreated lesioned rats, suggesting that PZKKN-94 may enhance L-DOPA’s therapeutic effect (Friedman ANOVA test: c^2^ [31, *df* 1] = 17.64, *P* < .05). No synergistic effect between L-DOPA and PZKKN-94 after acute combined treatment was observed, probably due to the high variability within groups.

Chronic L-DOPA treatment completely restored the symmetry in the use of both forelimbs in both sessions, and PZKKN-94 did not affect L-DOPA efficacy (Friedman ANOVA test: c^2^ [31, *df* 1] = 31.00, *P* < .05). In the OFF phase, the L-DOPA effect was no longer visible; however, groups pretreated with 2 higher doses of PZKKN-94 exhibited an improvement in the use of the IF compared with the same groups of untreated lesioned rats, suggesting that PZKKN-94 may enhance L-DOPA therapeutic effect and attenuate L-DOPA–induced motor fluctuation (Friedman ANOVA test: c^2^ [31, *df* 1] = 15.70, *P* < .05) (Fig. 3D).

### 3.6 PZKKN-94 reverses cognitive deficits compromised by NMDA receptor antagonist PCP and anticholinergic scopolamine and displays a procognitive effect in aged Wistar Han rats in the NOR test

PCP, like many other NMDA receptor antagonists, is used to pharmacologically model certain symptoms of psychoses, including cognitive deficits (Zajdel et al, 2016). In this experiment, PZKKN-94 pretreated animals received PCP before the acquisition trial and were tested 60 minutes later. One-way ANOVA demonstrated that the DI in PCP-treated rats was significantly lower than in the control group (*P* < .001, Tukey’s HSD test following a significant treatment effect: *F*[4,37] = 22.19, *P* < .05; Fig. 4A). This object recognition deficit was reversed by all tested doses of PZKKN-94 (ie, 0.3, 1, and 3 mg/kg). A significant difference was observed between vehicle + PCP-treated and PZKKN-94 + PCP-treated groups of at least *P* < .05 (Tukey’s HSD test, Fig. 4A).

**Fig. 4:**
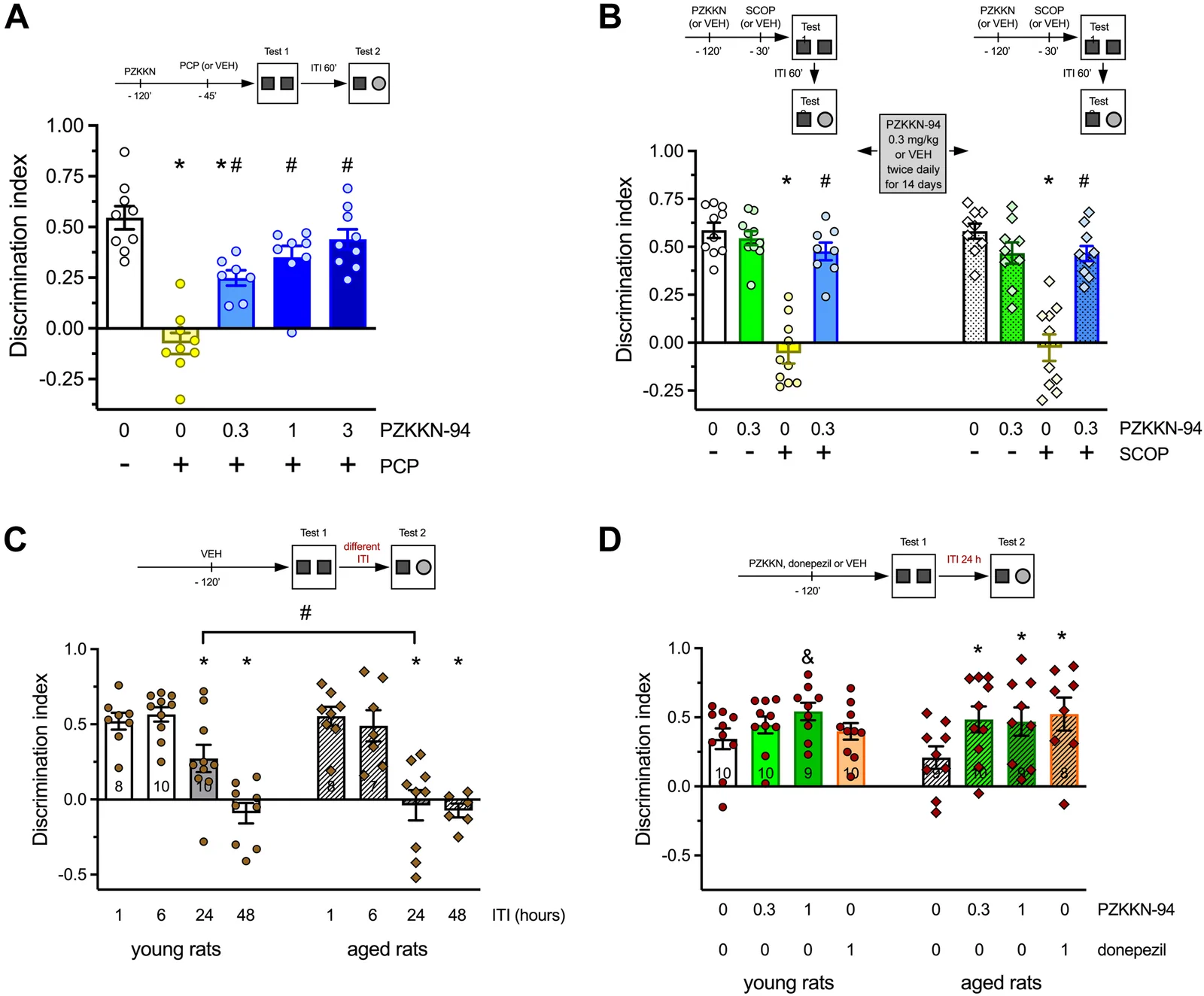
PZKKN-94 reverses PCP-induced (A) and scopolamine (SCOP)-induced (B) memory deficits in the NOR task in Sprague-Dawley rats and displays a procognitive effect in aged Wistar Han rats in the NOR test (D). Reversal of SCOP deficit was apparent both acutely and following 2 weeks of administration, as the NOR test was performed after a single administration (day 1) or 14 days of twice daily administration (day 14) of PZKKN-94. ^∗^*P* < .05, a significant reduction in DI compared with the vehicle (VEH)-treated group; ^#^*P* < .05, a significant improvement in DI compared with the PCP- or SCOP-treated group. (C) The young (10 weeks old) and aged (19 months old) Wistar Han rats demonstrate spontaneous forgetting in the NOR test, dependent on the delay between test 1 and test 2; however, at the intertrial interval (ITI) of 24 hours, the old animals display more severe deficits. ^∗^*P* < .05, significant deterioration of the respective DI compared with ITI of 1 hour; ^#^*P* < .05 shows the difference between young and aged animals at the ITI of 24 hours (gray bars). (C) While young (10 weeks old) and aged (19 months old) Wistar Han rats display a learning deficit at the long ITI of 24 hours, (D) PZKKN-94 and donepezil (a “positive” control) improve learning in aged animals only. ^∗^*P* < .05, a significant improvement in DI compared with the respective VEH-treated control group and an insignificant (*P* = .094) effect of PZKKN-94 in young rats. Data are shown as the mean ± SEM of the DI; *n* = 6–10 rats per group.

Anticholinergic compounds like scopolamine produce reliable learning deficits, which is considered a pharmacological model of dementia (Potasiewicz et al, 2020). We first examined whether scopolamine induces learning deficit and then whether PZKKN-94 affects it. The second goal of this experiment was to elucidate whether tolerance develops to the purported procognitive effects of PZKKN-94 because procognitive medications are likely to be used on a long-term basis by the patients. Following acute administration of the compounds, PZKKN-94 was administered twice daily, and the same animals were tested in the same treatment conditions.

Two-week treatment with vehicle or PZKKN-94 did not modify the response of animals to the compound and scopolamine, as the interaction between weeks (repeated factor) and PZKKN-94 and scopolamine treatment (between-subjects factors) was not significant: *F*(1,32) < 1, suggesting that tolerance does not develop to the procognitive effects of PZKKN-94 (Fig. 4B; Table 2).

However, PZKKN-94 × scopolamine interaction was strongly significant: *F*(1,32) = 91.278; *P* < .0001, and the Newman-Keuls post hoc test on data collapsed by weeks showed (1) a significant diminution of DI by scopolamine *P* < .05 and (2) that the pretreatment with PZKKN-94 prevented scopolamine-induced learning deficit (*P* < .05). PZKKN-94 per se did not affect NOR response.

The experiment evaluating age-related spontaneous forgetting (Fig. 4C) demonstrated that while the young (10 weeks old) and drug-naïve aged (19 months old) rats display a cognitive deficit, in the old animals, it was more severe at the intertrial interval of 24 hours. While expected, this is not a trivial observation because it directly shows a learning deficit related to natural aging (ie, not induced by pharmacological manipulation) in the present experimental conditions.

At the intertrial interval of 24 hours, the effects of PZKKN-94 and donepezil (the “positive” control) were compared in both age groups, and two-way ANOVA showed insignificant interaction between age and treatment: *F*(3,67) = 0.968; *P* = .412, suggesting a similar response to the treatments independent of age (Fig. 4D). Thus, in contrast to the experiment carried out in drug-free rats (see above), this set of data, specifically a priori planned contrast analyses, did not demonstrate differences in response to the vehicle (*P* = .246). However, the treatment factor was significant: *F*(3,67) = 3.050; *P* < .05, allowing further analyses. A priori planned contrasts revealed that PZKKN-94 given at 0.3 and 1 mg/kg as well as donepezil at 1 mg/kg restored aged but not young rats’ cognitive deficit (*P* < .05; Fig. 4D).

### 3.7 PZKKN-94 increases cognitive flexibility in ASST

While the effects of PZKKN-94 in 3 models of cognitive deficits appeared coherent and convincing, the NOR test represents a simple visual recognition. In contrast, ASST examines cognitive flexibility, a function compromised in psychiatric disorders like, for example, PD (Gruszka et al, 2017). Compound PZKKN-94 at a dose of 1 and 3 mg/kg, but not 0.3 mg/kg, significantly and specifically facilitated rats’ performance in the ED stage of the ASST, as indicated by a reduced number of trials to criterion during this phase (*P* < .001 for 1 and 3 mg/kg, Tukey’s HSD test following a significant treatment × discrimination phase interaction: *F*[18,168] = 34.59, *P* < .001; Fig. 5; Table 2). There was no significant drug effect during any other discrimination stage.

**Fig. 5:**
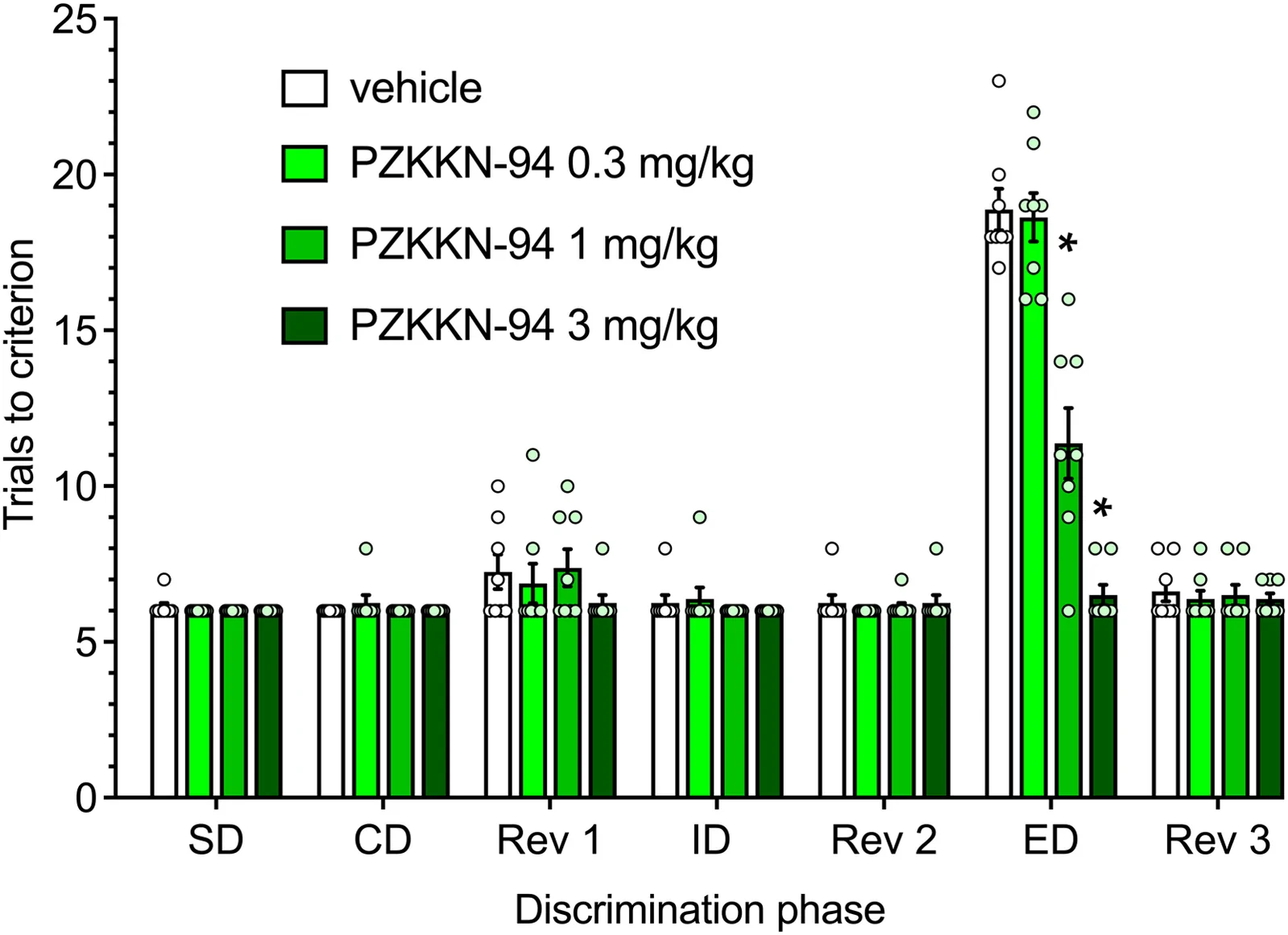
PZKKN-94 enhances cognitive flexibility in the ASST. The bars represent the mean ± SEM number of trials required to reach the criterion of 6 consecutive correct trials for each discrimination phase. *n* = 8 rats per group. ^∗^*P* < .05, a significant improvement in ED performance compared with the vehicle-treated control group. CD, compound discrimination; ID, intra-dimensional; Rev, reversal; SD, simple discrimination.

### 3.8 PZKKN-94 produces antidepressant-like activity in FST

In modified FST, the compound PZKKN-94 administered at a dose of 0.1 mg/kg significantly shortened the immobility time by 60% and increased climbing time by 191% while at a dose of 0.3 mg/kg decreased immobility by 33% and extended climbing by 77% (*F*[4,39] = 18.67, *P* < .0001 for immobility time and *F*[4,39] = 17.17, *P* < .0001 for climbing time; Supplemental Fig. 11; Table 2). PZKKN-94 at active antidepressant doses did not change the spontaneous locomotor activity of rats in the open field test (Supplemental Table 5) (Grychowska et al, 2016).

## 4 Discussion

We examined whether the compound PZKKN-94, developed as the first-in-class dual-acting 5-HT_1B_R agonist/5-HT_6_R antagonist, could be used for the treatment of motor and nonmotor symptoms of PD. We found that compound PZKKN-94 shows 10-fold more potent agonist properties in rat and m5-HT_1B_R orthologs compared with h5-HT_1B_R (Fig. 2E). Thus, compound PZKKN-94 does not show interspecies differences in our rodent models that preclude target engagement, which is often an issue for 5-HT_1B_R ligands (Oksenberg et al, 1992). For example, sumatriptan or 8-OH-DPAT behave as more potent ligands for h5-HT_1B_R than for r5-HT_1B_R (Oksenberg et al, 1992). Further, compound PZKKN-94 displays almost equipotent antagonist properties at rat and h5-HT_6_R orthologs, and it is 10-fold less potent at m5-HT_6_R (Fig. 2F). Although this aspect requires further exploration, it supports the target engagement by compound PZKKN-94 measured in rat preclinical models evaluated in our study. Moreover, in vitro ADME properties and pharmacokinetics results for PZKKN-94 support sufficient exposition to engage the target proteins in the brain (Fig. 6). The concentration of PZKKN-94 in the brain, including the free drug concentration at time of maximum concentration, reaches 185 nM. Assuming linear pharmacokinetics, this concentration is sufficient to achieve the EC_50_/*K*_B_ values for 5-HT_1B_R and 5-HT_6_R, even at a dose of 0.3 mg/kg in tested in vivo rat models.

**Fig. 6:**
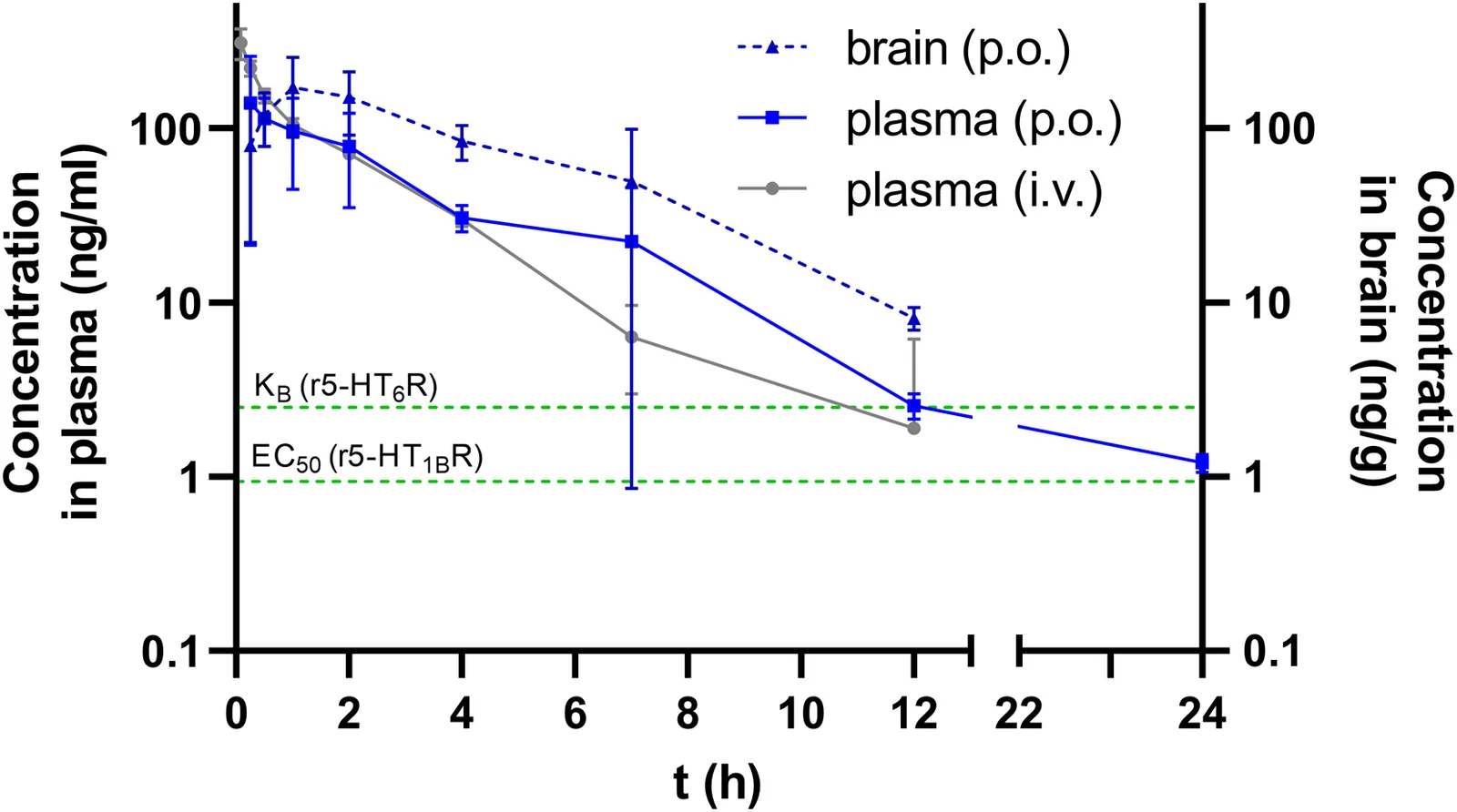
The pharmacokinetic profile of PZKKN-94 in rats demonstrates exposures for effective target engagement. PZKKN-94 was administered orally (p.o.) and intravenously in a dose of 10 mg/kg (*n* = 5, sparse sampling) and 3 mg/kg (*n* = 5, serial sampling), respectively. Brain concentration was assessed only after oral gavage. The left and right Y-axis depict concentration in plasma (ng/mL) and in the brain (ng/g tissue) on a semilogarithmic scale, respectively. Data are the mean ± SD. The dotted lines present a measure of agonist activity EC_50_ at 5-HT_1B_R (EC_50_ = 2.5 nM) and antagonist activity *K*_B_ at 5-HT_6_R (*K*_B_ = 6.6 nM) for PZKKN-94.

The present study demonstrates for the first time that the combined 5-HT_1B_R agonism and 5-HT_6_R antagonism produced antiparkinsonian effects in 6-OHDA–induced unilateral degeneration of the nigrostriatal system (Fig. 3A) and reduced haloperidol-induced catalepsy (Supplemental Fig. 7; Table 2). PZKKN-94 reversed motor deficits in the stepping test, indicative of gait problems resembling bradykinesia in patients with PD (Fig. 3A). In the vibrissae test examining sensorimotor deficits, though ineffective when administered alone, PZKKN-94 augmented the effect of the acute L-DOPA administration (Fig. 3D). Combined chronic administration of PZKKN-94 with L-DOPA did not affect the therapeutic-like effects of L-DOPA in the ON phase in either test (Fig. 3, B and D; Supplemental Fig. 8B). However, the compound increased the duration of L-DOPA action in the OFF phase in both tests (Fig. 3, B and D). Of note, neither acute nor chronic PZKKN-94 treatment influenced the L-DOPA–induced contralateral rotation or affected its anticataleptic effect (Supplemental Fig. 8B; Supplemental Fig. 9; Table 2). Thus, antiparkinsonian-like effects of PZKKN-94, together with no reduction of therapeutic L-DOPA efficacy, suggest that the combination of 5-HT_1B_R agonism and 5-HT_6_R antagonism may overcome the therapeutic burden of dual-acting 5-HT_1A_R/5-HT_1B_R agonists (Cenci et al, 2022). Accordingly, earlier findings have demonstrated that local activation of 5-HT_1B_R in the globus pallidus following administration of CP-93129, a purported 5-HT_1B_R selective agonist, attenuated reserpine-induced akinesia and haloperidol-induced catalepsy in rats, suggesting a prominent role of 5-HT_1B_R in reducing the overactive striatopallidal GABA pathway (Chadha et al, 2000; Wang et al, 2013). Furthermore, CP-94253, another purported selective 5-HT_1B_R agonist, attenuated dyskinesia in 6-OHDA–lesioned rats treated with L-DOPA (Carta et al, 2007; Zhang et al, 2008), suggesting that 5-HT_1B_R agonism may counteract hyperactivity of the striatonigral GABA neurons and might be used in patients with PD to modify the therapeutic window of L-DOPA treatment. However, the antidyskinetic effect of CP-94253 was accompanied by a decrease in L-DOPA–induced rotational behavior, indicating that selective 5-HT_1B_R agonists may diminish the antiakinetic effect of L-DOPA and thus the effectiveness of L-DOPA therapy (Dekundy et al, 2007).

PZKKN-94 effects corroborated previous findings with the selective 5-HT_6_R antagonist SB-258585, which reversed haloperidol-induced catalepsy (Ohno et al, 2015). Electrophysiological work by Bonsi et al, (2007) demonstrated that stimulation of 5-HT_6_R in the striatum activated striatal acetylcholine interneurons, which was reversed by a 5-HT_6_R antagonist. Considering the functional predominance of cholinergic over dopaminergic transmission in the PD striatum (Ztaou and Amalric, 2019), it can be hypothesized that an inhibitory effect of 5-HT_6_R antagonists on the activity of cholinergic interneurons may be involved in antiparkinsonian mechanisms (Dawson et al, 2003). One can only assume that 5-HT_1B_R and 5-HT_6_R expressed on GABAergic efferent striatal pathways and on glutamatergic neurons in the cortex (Gérard et al, 1997; Zhang et al, 2008) may also contribute to the antiparkinsonian effects of PZKKN-94 via synergistic interstructural brain mechanisms.

In patients with PD, the progressive degeneration of dopaminergic neurons also affects serotonergic, noradrenergic, and cholinergic systems and translates into diverse nonmotor disease manifestations, including cognitive impairment, depression, and sleep disturbances. To evaluate the impact of compound PZKKN-94 on cognitive processes, we used a battery of behavioral tests involving NOR and ASST in various cognition impairment conditions. PZKKN-94 reversed both PCP- and scopolamine-induced cognitive deficits, most likely preventing the negative effect of blockade of glutamatergic (via NMDA receptor) and acetylcholine (via muscarinic receptor) transmission on cognitive process (Fig. 4, A and B; Table 2). Of note, compound PZKKN-94 reversed scopolamine-induced learning deficits in the NOR test not only following acute but also following 2 weeks of administration, suggesting that the tolerance to its procognitive (and, probably, other) effects is unlikely to develop. These findings corroborated our previous reports on the procognitive properties of selective 5-HT_6_R antagonists in animal models of memory impairment (Zajdel et al, 2016). It is also known that the anticholinergic drugs, useful in PD therapy to treat tremors, accelerate cognitive impairment (Ehrt et al, 2010). Finally, in the age-related learning deficit model (Fig. 4C), PZKKN-94, as well as donepezil, the clinically available acetylcholinesterase inhibitor, produced a procognitive effect in aged but not young animals (Fig. 4D).

Following the observation that patients with PD show difficulties in switching attention (Gauntlett-Gilbert et al, 1999), we also examined the effects of compound PZKKN-94 in the ASST (Fig. 5; Table 2). This sophisticated test examines the ability to flexibly alter behavior in response to stimulus change. Compound PZKKN-94 enhanced cognitive flexibility in a dose-related manner, likely due to the expression of 5-HT_6_R and 5-HT_1B_R in the prefrontal cortex, the brain area involved in complex cognitive processes.

Depression is one of the most frequent nonmotor symptoms in patients with PD, with a prevalence of 38% (Cong et al, 2022); it appears in the early stage of the PD (or may even predate PD diagnosis) and persists throughout the whole disease duration, negatively impacting the life quality of patients with PD (for the review, see Ossowska and Lorenc-Koci, 2013). The present study suggests that PZKKN-94 displays antidepressant-like activity in rats without affecting the motor activity of the animals (Supplemental Fig. 11; Supplemental Table 5; Table 2). These results are consistent with recent findings suggesting the contribution of agonistic properties at 5-HT_1B_R to the efficacy of vortioxetine in the treatment of major depression (De Diego-Adeliño et al, 2022) and our previous data on antidepressant-like effects of 5-HT_6_R antagonists (Zajdel et al, 2016; Partyka et al, 2019). Thus, PZKKN-94 may offer patients with PD significant antidepressant benefits while reducing the risk of adverse events associated with polypharmacy.

The present report suffers from a number of limitations. Not all in vivo tests included a “positive” control. While potentially helpful, we are of the opinion that they would unnecessarily expand an already rich dataset; some of the antiparkinsonian, cognitive, and antidepressant-like effects of compounds from this laboratory, with numerous controls, were already published (Nikiforuk, 2014; Konieczny et al, 2014; Zajdel et al, 2016; Partyka et al, 2019). We are also aware that the concept of dual-acting 5-HT_1B_R/5-HT_6_R compounds would benefit from the measurement of the neurotransmitters’ concentration (ie, dopamine, acetylcholine, and GABA) in the striatum and prefrontal cortex.

We believe that our work on dual-acting compounds targeting 5-HT_1B_R/5-HT_6_R contributes to a better understanding of the serotonergic system’s role in PD treatment. Earlier studies suggest that a synergistic combination of agonism at 5-HT_1A_R and 5-HT_1B_R may be effective in treating L-DOPA–induced dyskinesia. Clinical data support the importance of inverse agonists at 5-HT_2A_R in alleviating psychosis associated with PD. Additionally, the selective blockade of 5-HT type 3 receptors (5-HT_3_R) has been suggested to alleviate L-DOPA–induced dyskinesia in marmosets (Aboulghasemi et al, 2019; Kwan et al, 2021). However, the combination of antagonism at 5-HT_6_R with 5-HT_2A_R antagonism did not improve cognition in phase 2 trials involving patients with dementia with Lewy bodies and PD dementia (Fernandez et al, 2023). Further multitargeted approaches (eg, 5-HT_6_R/monoamine oxidase type B, 5-HT_6_R/serotonin transporter, 5-HT_6_R/5-HT_3_R, 5-HT_6_R/5-HT_3_R/monoamine oxidase type B, and 5-HT_6_R/GABA-A) designed to alleviate symptoms of neurodegenerative and psychiatric disorders await validation in clinical settings (Canale et al, 2020; Zajdel et al, 2021; Grychowska et al, 2023; Marcinkowska et al, 2023; Siwek et al, 2024).

In conclusion, we demonstrate a novel nondopaminergic approach for the treatment of PD based on simultaneous activation of 5-HT_1B_R and blockade of 5-HT_6_R. Present results indicate that compound PZKKN-94 may be useful as an adjunctive therapy to L-DOPA to increase its efficacy and attenuate motor fluctuations (“on-off” phenomena). This finding may be particularly relevant because the available antiparkinsonian medications, though clearly effective in reducing dyskinesias, may worsen L-DOPA efficacy and produce a number of side effects. Furthermore, PZKKN-94 demonstrated procognitive and antidepressant-like activity without affecting motor functions. In toto, present observations suggest the utility of the combined 5-HT_1B_R agonism and 5-HT_6_R antagonism as a potential mechanism of novel treatments of PD.
