# Supplementary material for: Simultaneous 5-HT1BR agonist/5-HT6R antagonist action as a potential treatment of Parkinson’s disease and its comorbidities
Source: J Pharmacol Exp Ther. 2024 Dec 9;392(2):100055. doi: 10.1016/j.jpet.2024.100055 (PMC13727970; doi:10.1016/j.jpet.2024.100055)
Supplement: Supplementary file 1 [file mmc1.pdf]

Journal of Pharmacology and Experimental Therapeutics
manuscript number **JPET-AR-2024-002288R1**

Supporting Information

Simultaneous 5-HT_{1B}R agonist/5-HT_6R antagonist action as a potential treatment of Parkinson's disease and its comorbidities

Paweł Zajdel,[1,#,*] Mikołaj Matłoka,[2,#] Jolanta Konieczny,[3] Tomasz Kos,[3] Josie C. Lammers[4], Natalie G. Cavalco[4], Allison A. Clark[4], Tomasz Lenda,[3] Grzegorz Satała,[3] Vittorio Canale,[1] Katarzyna Grychowska,[1] Martyna Krawczyk,[3] Agnieszka Nikiforuk,[3] Anna Partyka,[1] Magdalena Jastrzębska-Więsek,[1] Klemencja Berghauzen-Maciejewska,[3] Dominika Biała,[3] Monika Janicka,[3] Artur Janusz,[3] Radosław Piast,[3] Krzysztof Mulewski,[3] Damian Smuga,[3] Jerzy Pieczykolan,[2] Maciej Wieczorek,[2] Rafał Moszczyński-Pętkowski,[2] Krzysztof Dubiel,[2] Krystyna Ossowska,[3] Andrzej J. Bojarski,[3] Krzysztof Kamiński,[1] John D. McCorvy,[4,5,6] Piotr Popik[2]

[1]Faculty of Pharmacy, Jagiellonian University Medical College, 9 Medyczna Str, 30-688 Kraków, Poland

[2]R&D Centre, Celon Pharma SA, 15 Marymoncka Str, 05-152 Kazuń Nowy, Poland

[3]Maj Institute of Pharmacology Polish Academy of Sciences, 12 Smętna Str, 31-343 Kraków, Poland

[4]Department of Cell Biology, Neurobiology and Anatomy, Medical College of Wisconsin, Milwaukee, WI 53226, USA

[5]Neuroscience Research Center, Medical College of Wisconsin, Milwaukee, WI 53226, USA

[6]Cancer Center, Medical College of Wisconsin, Milwaukee, WI 53226, USA

[#]these authors contributed equally

*Corresponding author: Paweł Zajdel

Email: pawel.zajdel@uj.edu.pl

Table of contents

1. General Chemical Methods 4
1.1. Synthesis of the compound PZKKN-94 4
2. *In vitro* biological evaluation of compound PZKKN-94 6
2.1. Human and Rodent 5-HT$_{1B}$R, 5-HT$_6$R, and 5-HT$_{1F}$R G protein dissociation BRET assays 6
2.2. Determination of the functional activity at human 5-HT$_{1B}$R 6
2.3. Determination of the functional activity at human 5-HT$_6$R 7
2.4. Selectivity screen 7
2.5. Radioligand binding assays for human 5-HT$_{1B}$R 8
2.6. Radioligand binding assays for human 5-HT$_6$R 8
2.7. Determination of the half-maximal effective concentration (EC50) against or half-maximal inhibitory concentration (IC50) against selected targets 9
2.8. Assessment of compound PZKKN-94 metabolic stability in rat liver microsomes assay 9
2.9. Assessment of compound PZKKN-94 binding to plasma proteins 9
2.10. Assessment of compound PZKKN-94 activity at CYP3A4 and CYP2D6 10
2.11. Assessment of cell permeability 10
2.12. Assessment of compound PZKKN-94 mutagenicity 11
3. *In vivo* pharmacological properties of compound PZKKN-94 12
3.1. Drug treatment 12
3.2. Animals and ethical statements 12
3.2.1. Animals for pharmacokinetic assessment, FST, and open field (OF) test 12
3.2.2. Animals for the stepping, vibrissae and catalepsy tests 13
3.2.3. Animals for NOR test and ASST 13
3.3. Experimental design, statistical analysis and requirements for studies using animals 13
3.3.1. Estimation of required sample analysis 13
3.3.2. Randomization, blinding and exclusion criteria 14
3.4. Methods 15
3.4.1. Pharmacokinetic profile of PZKKN-94 15
3.4.2. 6-OHDA-induced lesion 15
3.4.3. Rotational behavior 15
3.4.4. Novel object recognition test 16
3.4.5. Attentional set shifting task 16
3.4.6. Open Field Test. 17
4. Results 18
4.1. 6-OHDA induces motor deficits 18
4.2. PZKKN-94 does not enhance catalepsy produced by 6-OHDA lesion 18
4.3. Chronic administration of PZKKN-94 does not reduce therapeutic effect of L-DOPA in the rotational test 18
4.4. PZKKN-94 displays antidepressant-like properties 18
Supplemental Figures
Supplemental Figure 1. UPLC chart and MS spectra for PZKKN-94 19
Supplemental Figure 2. ^{1}H NMR and ^{13}C NMR spectra for PZKKN-94 20

Supplemental Figure 3. Experimental schedule of testing rats lesioned unilaterally with 6-OHDA........ 21
Supplemental Figure 4. Effect of PZKKN-94 on human 5-HT_{1F} G protein, on human and rodent 5-HT_{1B} G(oB) and on human and rodent 5-HT_6 Gs protein dissociation activity 22
Supplemental Figure 5. Rat plasma binding protein for PZKKN-94, atenolol, lidocaine and warfarin, used as references 23
Supplemental Figure 6. The impact of PZKKN-94 on *in vitro* CYP3A4 and CYP2D6 activity 24
Supplemental Figure 7. PZKKN-94 decreases the haloperidol-induced catalepsy in rats 25
Supplemental Figure 8. The effect of PZKKN-94 and L-DOPA on performance of the catalepsy test in unilaterally 6-OHDA-lesioned rats 26
Supplemental Figure 9. PZKKN-94 does not affect L-DOPA-induced contralateral rotations in the unilaterally 6-OHDA-lesioned rats suggesting no influence on the therapeutic effect of L-DOPA. 27
Supplemental Figure 10. The motor deficits produced by 6-OHDA three weeks after lesion.. 28
Supplemental Figure 11. The effects of PZKKN-94 on immobility and climbing time in FST rats 29

Supplemental Tables

Supplemental Table 1. Selectivity of PZKKN-94 at10 µM. This table presents percent activity or inhibition of a specific control. “Interaction” is denoted as value > 70% 30
Supplemental Table 2. Functional potency (IC_{50} or EC_{50}) for PZKKN-94 against targets selected based on the selectivity screen........ 32
Supplemental Table 3. Membrane permeability of PZKKN-94 assessed in a Caco2 assay . 33
Supplemental Table 4. Genotoxicity assessment of PZKKN-94 in Ames test with presence or absence of S9 metabolic fraction........ 34
Supplemental Table 5. Effect of compound PZKKN-94 on rat locomotor activity measured in the open field test........ 36
Supplemental Table 6. F and p values of both behavioral data presented in the results section 37

1. General Chemical Methods

Organic solvents (from Merck and Chempur) were of reagent grade and were used without purification. Commercially available reagents were of the highest purity (from Aldrich, Fluorochem). Solution transformations were carried out at ambient temperature, unless indicated otherwise. All workup and purification procedures were carried out with reagent-grade solvents under ambient atmosphere. Column chromatography was performed using silica gel Merck 60 (70–230 mesh ASTM).

^{1}H and ^{13}C NMR spectra were recorded on a Varian BB 300 spectrometer at 300 and 75 MHz, respectively, and were reported in ppm using deuterated solvent for calibration ($CDCl_3$). The *J* values were reported in hertz (Hz), and the splitting patterns were designated as follows: br s. (broad singlet), s (singlet), d (doublet), t (triplet), td (triplet of doublets), m (multiplet).

Mass spectra were recorded on a UPLCMS/MS system consisting of a Waters ACQUITY UPLC (Waters Corporation, Milford, MA, USA) coupled to a Waters TQD mass spectrometer (electrospray ionization mode ESI-tandem quadrupole). Chromatographic separations were carried out using the Acquity UPLC BEH (bridged ethyl hybrid) 18 column × 2.1 mm × 100 mm, and 1.7 μm particle size, equipped with Acquity UPLC BEH C18 Van Guard precolumn; 2.1 mm × 5 mm, and 1.7 μm particle size. The column was maintained at 40 ℃ and eluted under gradient conditions from 95% to 0% of eluent A over 10 min, at a flow rate of 0.3 mL min^{-1}. Eluent A: water/formic acid (0.1%, *v*/*v*), Eluent B: acetonitrile/formic acid (0.1%, *v*/v). The UPLC/MS purity of all the final compounds was confirmed to be 95% or higher.

1.1. Synthesis of the compound PZKKN-94

The synthesis of compound PZKKN-94 (2-[(2,4-difluorophenyl)sulfonyl]-4-(piperazin-1-yl)isoindoline hydrochloride MW = 415.89), was performed following the first disclosure (Patent PCT/IB2017/057247) (Kamiński et al., 2019). Briefly, the first step consisted in the microwave-assisted *N*-arylation of *N*-acetyl 4 bromo-isoindoline **1** with Boc-piperazine yielding intermediate **2** (1-[4-(piperazin-1-yl)isoindolin-2-yl]ethan-1-one) (Scheme S1). After a one-pot deacetylation/sulfonylation procedure in aqueous basic media, final compound was obtained upon deprotection of the Boc function of intermediate **3** using HCl methanolic solution.

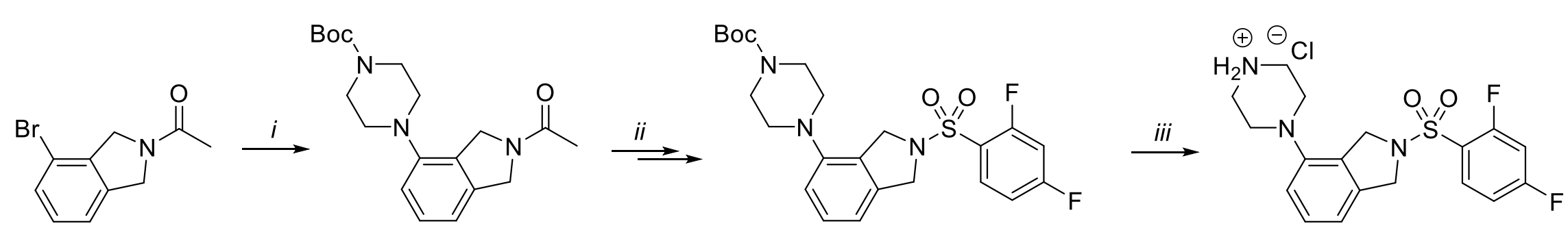

Scheme S1. Synthetic route of compound PZKKN-94, reaction and conditions: *i*) Boc-piperazine (2 eq), Pd_2dba_3 (0.04 eq), BINAP (0.06 eq), sodium *tert*-butoxide (1.4 eq), dioxane$_{anh}$, 1.5 h, 130 °C (yield 82%); *ii*) EtOH/10% $NaOH_{aq}$ (1/2, *v*/*v*), 2 h, 70 °C then 2,4-difluorobenzenesulfonyl chloride (1.2 eq), CH_2Cl_2, 2 h, 0 °C→rt, (yield 70%); *iii*) EtOH, 4M HCl in MeOH, 16 h, rt, (yield 97%).

1.1.1. 1-[4-(Piperazin-1-yl)isoindolin-2-yl]ethan-1-one (**2**)

N-acetyl 4 bromo-isoindoline (500 mg, 1.5 mmol, 1 eq), Pd_2dba_3 (55 mg, 0.06 mmol, 0.04 eq), BINAP (56 mg, 0.09 mmol, 0.06 eq), sodium *tert*-butoxide (200 mg, 2.1 mmol, 1.4 eq) and Boc-piperazine (600 mg, 3.0 mmol, 2 eq) were solubilized in anhydrous dioxane (15 mL) and a resulting mixture was submitted under microwave irradiation at 130 °C for 1.5 h under argon atmosphere. The resulting mixture was filtrated through a Celite pad, concentrated and purified by column chromatography on silica gel using CH_2Cl_2/MeOH (9/0.3, *v*/*v*), as a developing solvent, to yield

compound **2** as white solid (425 mg, isolated yield 82%); UPLC/MS purity 100%, t_R = 4.83; $C_{19}H_{27}N_3O_3$, MW 345.44, Monoisotopic Mass 345.21, $[M+H]^+$ 346.2. ^1H NMR (300 MHz, $CDCl_3$) δ ppm 1.45 (s, 9H), 1.53 (s, 3H), 3.22–3.25 (m, 4H), 3.51–3.55 (m, 4H), 4.33 (s, 2H), 4.77 (s, 2H), 7.18–7.43 (m, 3H).

1.1.2 Tert-*butyl 4-(2-((2,4-difluorophenyl)sulfonyl)isoindolin-4-yl)piperazine-1-carboxylate* (**3**)

Compound **2** (400 mg, 1.16 mmol, 1 eq) was solubilized in EtOH (10 mL) followed by the addition of 10% NaOH aqueous solution (20 mL) and the reaction mixture was stirred for 2h at 70 °C. After evaporation of EtOH and addition of CH_2Cl_2 (8 mL), a solution of 2,4-difluorobenzenesulfonyl chloride (0.21 mL, 1.58 mmol, 1.2 eq) in CH_2Cl_2 (2 mL) was added dropwise to the suspension at 0 °C. The mixture was stirred for 2 h at room temperature. Then, the organic phase was separated, washed with water (1 x 5 mL), brine (1 x 5 mL), dried over Na_2SO_4, filtered and concentrated. The crude product was purified by column chromatography on silica gel using AcOEt/Hex (3/7, *v*/*v*) as a developing solvent, yielding compound **3** as white solid (390 mg, isolated yield 70%); UPLC/MS purity 100%, t_R = 8.07; $C_{23}H_{27}F_2N_3O_4S$, MW 479.54, Monoisotopic Mass 479.17, $[M+H]^+$ 480.2. ^1H NMR (300 MHz, $CDCl_3$) δ ppm 1.44 (s, 9H), 2.86 (t, J = 5.1 Hz, 4H), 3.48–3.55 (m, 4H), 4.31 (s, 2H), 4.75 (s, 2H), 6.86–6.95 (m, 1H), 6.96–7.03 (m, 1H), 7.18–7.25 (m, 1H), 7.30–7.38 (m, 1H), 7.55–7.65 (m, 1H), 7.92–8.01 (m, 1H).

1.1.3 (2-[(2,4-Difluorophenyl)sulfonyl]-4-(piperazin-1-yl)isoindoline hydrochloride (**PZKKN-94**)

To a solution of compound **3** (350 mg, 0.73 mmol) in anhydrous EtOH (10 mL), 4M solution of HCl in MeOH (2 mL) was added. After 16 h, the precipitate was filtered and then washed with diethyl ether (3 x 5 mL) to yield PZKKN-94 as hydrochloride salt (white solid, 270 mg, isolated yield 97%); UPLC/MS purity 100%, t_R = 4.40; $C_{18}H_{19}F_2N_3O_2 \cdot HCl$, MW 415.89, Monoisotopic Mass 379.12, $[M+H]^+$ 380.1. ^1H NMR (300 MHz, DMSO-d_6) δ ppm 3.01–3.24 (m, 8H), 4.60 (s, 4H), 6.89 (d, J = 8.2 Hz, 1H), 6.96 (d, J = 7.0 Hz, 1H), 7.19–7.33 (m, 2H), 7.46–7.57 (m, 1H), 7.95 (td, J = 8.5, 6.4 Hz, 1H), 9.44 (br s, 2H). ^{13}C NMR (75 MHz, DMSO-d_6) δ ppm 43.5, 47.8, 52.4, 53.7, 106.8 (t, J_{C-F} = 19.6 Hz), 112.8 (d, J_{C-F} = 3.5 Hz), 113.12 (d, J_{C-F} = 2.3 Hz), 117.4, 117.9, 122.1 (d, J_{C-F} = 3.5 Hz), 122.3 (d, J_{C-F} = 3.5 Hz), 129.3, 129.6, 133.4 (d, J_{C-F} = 11.5 Hz), 138.0, 147.3. Elemental analysis calc. for $C_{18}H_{19}F_2N_3O_2 \cdot HCl$: C: 45.96%, H: 4.93%, N: 8.93%, S: 6.82%; found C: 45.75%, H: 5.16%, N: 8.78%, S: 6.53%.

2. *In vitro* biological evaluation of compound PZKKN-94

2.1. Human and Rodent 5-HT$_{1B}$R, 5-HT$_6$R, and 5-HT$_{1F}$R G protein dissociation BRET assays

Bioluminescence resonance energy transfer (BRET) assays were conducted using BRET in HEK293T cells (ATCC CRL-11268; mycoplasma-free), which were subcultured in high-glucose DMEM (VWR) supplemented with 10% FBS (Life Technologies) (Lewis et al., 2023). Human 5-HT$_{1B}$R, 5-HT$_{1D}$R, and 5-HT$_6$R constructs in G protein-dissociation BRET2 assays were derived from the codon-optimized Tango pcDNA3.1 library (Addgene) with V2tail/TEV/tTA encoding regions deleted to yield “de-Tango” constructs (Kroeze et al., 2015). Rat and mouse 5-HT$_{1B}$R and 5-HT$_6$R were cloned into pcDNA3.1 using Gibson Assembly from geneblocks synthesized by Integrated DNA Technologies (IDT). All Gα-Rluc8, Beta, and GFP2-γ constructs were derived from the TRUPATH pcDNA5/FRT/TO library (Addgene) (Olsen et al., 2020). Approximately 48 hours before assays, cells were transfected in 10% dialyzed (dFBS) in a 1:1:1:1 ratio of h/m/r5-HT$_{1B}$R or h5-HT$_{1F}$R: GαoB-Rluc8: Beta1: GFP2-γ2 constructs or 1:1:1:1 ratio of h/m/r5-HT$_6$R: Gαs(short)-Rluc8: Beta1: GFP^2-γ1 constructs. All transfections were prepared in Opti-MEM (Invitrogen) and used a 3:1 ratio of TransIT-2020 (Mirus) uL:ug total DNA. On the day before the assay, cells were detached using trypsin, centrifuged and concentrated, and plated in white 96-well plates (Grenier Bio-one) at an approximate density of 40,000 cells per well in 1% dFBS DMEM. Next day, plates were decanted and 60 uL of drug buffer per well (1 × HBSS, 20 mM HEPES, pH 7.4) was added using a Multidrop (ThermoFisher Scientific), and plates were allowed to equilibrate at 37°C in a humidified incubator before receiving drug stimulation. Drug dilutions of all compounds were performed in McCorvy buffer (1 × HBSS, 20 mM HEPES, pH 7.4, supplemented with 0.3% BSA fatty acid free (GoldBio), and 0.03% ascorbic acid). Drugs were dispensed into plates using a multichannel pipette spanning 100 μM to 10 pM concentrations. Next, plates were incubated with drug at 37 °C in a humidified incubator for 60 minutes. During 60 min compound incubation, addition of coelenterazine 400a (5 μM final concentration; Nanolight Technology) was performed 15 minutes before reading. Plates were read at 400 nm Rluc emission and GFP^2 emission at 510 nm at 1 second per well using a PheraStarFSX or at 0.65 second per well on a ClarioStar Plus (BMB LabTech). The BRET ratios of 510/400 (acceptor/donor) luminescence were calculated per well and were plotted as a function of compound concentration using Graphpad Prism 10 (Graphpad Software Inc., San Diego, CA). Data were normalized to % 5-HT control stimulation, which was present on every plate, and analyzed using nonlinear regression “log(agonist) *vs*. response” to yield E_{MAX} and EC_{50} parameter estimates. Antagonist 5-HT$_6$R assays utilized either 100 nM or 320 nM concentration of 5-HT and K_B was calculated from method by Cheng (2001) utilizing 5-HT EC_{50} for each respective 5-HT$_6$R ortholog and corresponding IC_{50} estimate for PZKKN-94.

2.2. Determination of the functional activity at human 5-HT$_{1B}$R

The agonist activity of PZKKN-94 was assessed according to protocol from DiscoverX. Briefly, enzyme Fragment Complementation (EFC) with β-galactosidase (β-Gal) as the functional endpoint method was used. cAMP Hunter cells were seeded into white walled, 384-well microplates. cAMP modulation was determined using the DiscoverX HitHunter cAMP XS+ assay. Cells were incubated with compounds at concentration range of 0.3 nM – 10 μM for 30 min followed by forskolin challenge at the EC_{80} concentration for another 30 min. After incubation, assay signal was generated and read following signal generation with a PerkinElmer Envision™ instrument for chemiluminescent signal detection. Compounds were tested in quintuplicate at eight concentrations in the range from 10^{-11} to 10^{-4} M.

2.3. Determination of the functional activity at human 5-HT$_6$R

Compound PZKKN-94 was examined on 5-HT$_6$R using their ability to inhibit cAMP production induced by 1 μM (EC$_{80}$) 5-carboxamidotryptamine (5-CT) (Zajdel et al., 2021). The level of cAMP was measured in 1321N1 cells expressing the h5-HT$_6$R (PerkinElmer, #ES-316-CF). According to the manufacturer's instructions, total cAMP was measured using the LANCE Ultra cAMP Detection Kit (PerkinElmer, #TRF0263). Cells were incubated with a mixture of compounds for 30 min at room temperature (RT) in a white polystyrene OptiPlate-384 (PerkinElmer, #6007299) microplate. After incubation, the reaction cells were lysed by the addition of 10 μL of cAMP detection buffer, including Eu-cAMP tracer and ULight-anti-cAMP working solution. The plate was incubated at RT for 1 h before measuring the signal with a Tecan multimode plate reader (Infinite M1000 Pro). Compound was tested in quintuplicate at eight concentrations in the range from 10^{-11} to 10^{-4} M. K_B constant was calculated from the Cheng-Prusoff equation adapted to functional assays (Cheng and Prusoff, 1973, Cheng 2001).

2.4. Selectivity screen

The Safety44Panel® assay was carried out by DiscoverX (Fremont, CA, USA) according to the service-provider's protocol. PZKKN-94 was tested at a concentration of 10 μM against a panel of 44 potential off-targets proteins, including G protein-coupled receptors (GPCRs), transporters, ion channels, nuclear receptors kinases and other non-kinase enzymes, by use of cells *in vitro* (Supp. Table 1). Briefly, for beta-1 adrenergic receptor and beta-2 adrenergic receptor antagonistic mode, Enzyme Fragment Complementation (EFC) with β-galactosidase (β-Gal) as the functional endpoint method was used. cAMP Hunter cells were plated into white walled, 384-well microplates. cAMP modulation was determined using the DiscoverX HitHunter cAMP XS+ assay. Cells were incubated with compounds at concentration range of 0.3 nM – 10 μM for 30 min followed by agonist challenge at the EC$_{80}$ concentration (betaxolol and ICI118,551, respectively) for another 30 min. After appropriate compound incubation, assay signal was generated and read following signal generation with a PerkinElmer Envision™ instrument for chemiluminescent signal detection.

IC$_{50}$ determination against cholinergic receptor muscarinic 1 and 5-hydroxytryptamine receptor 2A was performed by Calcium Mobilization method. Briefly, cells were plated into black-walled, clear-bottom, Poly-D-lysine coated 384-well microplates. Assays were performed in 1 x Dye Loading Buffer consisting of 1 x Dye, 1 x Additive A and 2.5 mM Probenecid in HBSS/20 mM Hepes. Cells were loaded with dye prior to testing and incubated for 30–60 min. at 37 °C. Cells were preincubated with sample compound at concentration range of 0.3 nM – 10 μM for 30 min at room temperature in the dark. Compound antagonist activity was measured on a FLIPR Tetra (MDS). Calcium mobilization was monitored for 2 minutes and EC$_{80}$ agonist in HBSS/20 mM Hepes was added to the cells 5 seconds into the assay.

For IC$_{50}$ determination against 5-hydroxytryptamine receptor 3A and sodium channel protein type 5 subunit alpha, Ion Channel Assay was applied. Briefly, cells were seeded into black-walled, clear-bottom, Poly-D-lysine coated, 384-well microplates. Assays were performed in 1 x Dye Loading Buffer consisting of 1 x Dye, and 2.5 mM Probenecid. Cells were loaded with dye prior to testing and incubated for 30–60 min. at 37 °C. For blocker format determination, cells were preincubated with sample at concentration range of 0.3 nM – 10 μM for 30 min at room temperature in the dark followed by addition of EC$_{80}$ of an agonist (1.45 μM of serotonin hydrochloride for 5-HT$_{3A}$ receptor) for the next 30 min. Compound activity was measured on a FLIPR Tetra (MDS). Compound activity was analyzed by the service provider using CBIS data analysis suite (ChemInnovation, CA).

For antagonist mode assay, percentage inhibition is calculated using the following formula:

$$\%\,inhibition = \frac{100\% * (1 - (mean\ RLU\ of\ test\ sample - mean\ RLU\ of\ vehicle\ control)}{(mean\ RLU\ of\ EC_{80}\ control - mean\ RLU\ of\ vehicle\ control)} \quad 1$$

For agonist mode assays, percentage activity is calculated using the following formula:

$$\% \, activity = \frac{100\% * (1 - (mean\ RLU\ of\ test\ sample - mean\ RLU\ of\ MAX\ control)}{(mean\ RLU\ of\ vehicle\ control - \ mean\ RLU\ of\ MAX\ control)} \quad 2$$

2.5. Radioligand binding assay for human 5-HT$_{1B}$R

2.5.1. Cell culture and preparation of cell membranes for radioligand binding assay

CHO-K1 cell line overexpressing *h*5-HT$_{1B}$ receptor was maintained at 37 °C in a humidified atmosphere with 5% CO_2 and grown in Dulbecco's Modifier Eagle Medium containing 10% dialyzed fetal bovine serum. For membrane preparation, cells were subcultured in 150 cm^2 flasks, grown to 90% confluence, washed twice with prewarmed to 37 °C phosphate-buffered saline (PBS), and pelleted by centrifugation (200 x g) in PBS. Pellets were stored at -80 °C.

2.5.2. Radioligand binding assay

Cell pellets were thawed and homogenized in 10 volumes of assay buffer using an Ultra Turrax tissue homogenizer and centrifuged twice at 35,000 g for 15 min at 4 °C. The incubation buffer consisted of 50 mM Tris HCl, 10 mM $MgCl_2$ and 0.1 mM EDTA. Assay was incubated in a total volume of 200 µL in a 96-well microplate for 0.5 h at 37 °C. The process of equilibration was terminated by rapid filtration through Unifilter plate with a 96-well cell harvester and radioactivity retained on the filter was quantified on a Microbeta plate reader (PerkinElmer, USA). For binding studies, the assay samples contained as radioligand 1 nM [^{3}H]-5-CT (PerkinElmer, USA). Non-specific binding was defined with 10 µM 5-HT. Compound was tested in quintuplicate at seven concentrations in the range from 10^{-10} to 10^{-4} M. The inhibition constants (K_i) were obtained from the Cheng−Prusoff model (Cheng and Prusoff, 1973). The acquired results were presented as means of two independent experiments.

2.6. Radioligand binding assay for human 5-HT$_6$R

All the experiments were carried out according to previously published procedures (Zajdel et al., 2018, 2021). In brief, HEK293 cells with stable expression of *h*5-HT$_6$ receptor was grown in Dulbecco's modified Eagle medium containing 10% dialyzed fetal bovine serum and 500 µg/mL G418 sulfate. For membrane preparation, cells after reaching 90% confluence, were washed with phosphate buffered saline (PBS), and pelleted by centrifugation (200 × g) in PBS containing 0.1 mM EDTA and 1 mM dithiothreitol. The cell pellets were homogenized in assay buffer using a tissue homogenizer (Ultra Turrax IKAT25), centrifuged twice (35000 × g, 15 min, 4 °C), and incubated (15 min, 37 °C) between centrifugation rounds. The buffers used were dedicated to a given type of receptor, and their composition was the same as in previously published articles. All assays were incubated in 96-well round bottom microwell plates for 1 h at 37 °C. The total reaction volume was 200 µL. The incubation process was terminated by filtration through UniFilter-96 (PerkinElmer) plates with the FilterMate Universal Harvester (PerkinElmer, #C961962), and radioactivity retained on the filters was quantified on a MicroBeta counter for radiometric detection (PerkinElmer). For competitive studies, the assay samples contained as radioligands: 2 nM [^{3}H]-LSD (PerkinElmer, # NET638250UC) for 5-HT$_6$R. K_D parameter for LSD equals 1.8 nM.

To evaluate the level of nonspecific signal 10 µM of methiothepine for 5-HT$_6$R was used. Compound was tested in quintuplicate at seven concentrations in the range from 10^{-10} to 10^{-4} M. The inhibition constants (K_i) were obtained from the Cheng−Prusoff model (Cheng and Prusoff, 1973). The acquired results were presented as means of two independent experiments.

2.7. Determination of the half-maximal effective concentration (EC_{50}) against or half-maximal inhibitory concentration (IC_{50}) against selected targets

The IC_{50} and EC_{50} assays for beta-1 adrenergic receptor, beta-2 adrenergic receptor, cholinergic receptor muscarinic 1, 5-hydroxytryptamine receptor 2A, 5-hydroxytryptamine receptor 3A, sodium channel protein type 5 subunit alpha were carried out by DiscoverX (Fremont, CA, USA) according to the service-provider's protocol (Supp. Table 2).

2.8. Assessment of compound PZKKN-94 metabolic stability in rat liver microsomes assay

Assessment of metabolic phase I stability in rat liver microsomes (Invitrogen) was performed on 96-well non-binding plates (Greiner) at 1 µM concentration for verapamil and donepezil (positive control) and PZKKN-94. Each biological replicate was prepared in triplicates. Briefly, compounds were incubated in 100 mM potassium phosphate buffer with microsomes (0.5 mg/mL) and NADPH (1.3 mM) on a plate shaker (500 rpm) in dark at 37 ºC. 4x solution of NADPH, cofactor for metabolic enzymes, was prepared directly prior to the experiment by reducing NADP with G6P dehydrogenase (3.3 mM $MgCl_2$, 3.3 mM G6P, 1.3 mM NADP, 3.2 U/mL G6P dehydrogenase, 20 min at 30 ºC, 500 rpm). The negative control contained buffer instead of NADPH solution. Samples were collected at 0, 10, 20 and 40 min or 0 and 40 min for the negative and double negative controls. The reaction was stopped by protein precipitation in 2 volumes of ice cold MeOH with 200 nM imipramine (as an internal standard for LC-MS analysis). Then, the extract was mixed (1 min, 1000 rpm), filtered through 0.22 µm filter on 96-well plate vacuum manifold and subjected to LC-MS analysis. LC-MS analysis was performed using a UPLC Waters H-Class coupled with TQD Waters detector (MS/MS) in the positive mode and multiple reaction monitoring (MRM) mode. The other MS parameters were as follows: desolvatation temperature 400°C, desolvataion gas flor 800 L/min, cone gas 45 psi, capillary voltage 1000 V. MRM transitions for KKN-94 were 380 → 175 (CE 24 V) and 380 → 83 (CE 34 V) at cone voltage 42V. MRM transitions for imipramine were 281.2 → 86.1 (CE 16 V) and 281.2 → 193.1 (CE 42 V) at cone voltage 30 V. MRM transitions for verapamil 455.3 → 165.0 (CE 32 V) and 455.3 → 150.0 (CE 46 V) at cone voltage 52 V. MRM transition for donepezil: 380.2 → 91.0 (CE 36 V), 380.2 → 243.2 (CE 30V) at cone voltage 56V. LC separation was achieved using an ACQUITY UPLC BEH C18 50 x 2.1 mm, 1.7 µm column (Waters) at 40 °C. The mobile phases were (A) 25 mM HCOOH in water and (B) 25 mM HCOOH in acetonitrile. Samples were eluted using a timed, linear gradient from 30% B at 0 min to 90% B at 1.5 min then a hold 90% B to 2.5 min, after it back to initial 30% B to 2.6 min. The columns was equilibrated for up to 5 min. The flow rate was 0.5 mL/min. The injection volume was 2 µL. Intrinsic clearance (Cl_{int}) was calculated using the rate of compound disappearance from the reaction. The elimination rate constant (k) was calculated as a module of slope of the linear regression from ln [substrate] *vs.* time. Then, clearance was calculated as $Cl_{int} = k/d$, where d is protein density for microsomal stability.

2.9. Assessment of compound PZKKN-94 binding to plasma proteins

Prewarmed rat plasma was spiked with a compound to the final concentration of (1 µM). 100 µL of spiked plasma was subsequently added to the "plasma" compartment of the RED (Rapid Equilibrium Dialysis; Thermo Scientific cat. 90006) device and the "buffer" compartment was filled with 300 µL of PBS. RED devices were incubated for 4 h in 37 °C. The bound fractions of compounds were stopped by dialysis membrane, while unbound compound diffused freely between both compartments, samples were collected in 3 replicates for every compound and concentration. Simultaneously, samples of freshly spiked plasma before the (t0) and after the incubation for 4 h in 37 °C (t4) in a separate vessel were prepared for stability and recovery assessment. After incubation samples from sections Plasma, Buffer, T4 and T0 were precipitated in MeOH with addition of known concentration of IS, centrifuged and supernatants were analyzed using the MS-MS method.

Retrieved supernatants were diluted in proportion 1 to 1 with deionized water in 96 well microplate. For data acquisition was used Exion AD LC chromatograph linked with Sciex QTRAP 5500+. Samples were separated on Acquity UHPLC BEH C18, 2.1 × 30 mm, 1.7 µm column with protective precolumn. Aqueous phase consisted of 0.1% acetic acid in miliQ H_2O, and organic one of 0.1% acetic acid in MS grade acetonitrile. MS analysis was operated in the positive ionization mode. Ionization conditions parameters were as follows: curtain gas (CUR) flow rate of 30 L/min, an ionization temperature (TEM) set at 400 °C, gas source 1 (GS1) and gas source 2 (GS2) both set to 50 L/min, and a collision-activated dissociation (CAD) gas flow rate of 8 L/min. Targeted detection was possible using MRM parameters for selected, specific fragmentation patterns of ionized compounds. Raw data was recorded as area under the curve (AUC) of peaks corresponding to compound and IS. Signals from compounds were normalized to IS AUC and processed in Graphpad Prism 9.5 for calculation of fraction bound, recovery and stability.

2.10. Assessment of compound PZKKN-94 activity at CYP3A4 and CYP2D6

The luminescent CYP3A4 P450-Glo™ and CYP2D6 P450-Glo™ assays and protocols were provided by Promega (Madison, WI, USA). The P450-Glo™ based on the conversion proluciferins, derivatives of beetle luciferin [(4S)-4,5-dihydro-2-(6´-hydroxy-2´-benzothiazolyl)-4-thiazolecarboxylic acid] into D-luciferin catalyzed by respective CYP isoform. D-luciferin is formed and detected in a second reaction with the Luciferin Detection Reagent (LDR). The amount of light produced in the second reaction is proportional to CYP activity.

The stock solutions (10 mM) of ketoconazole, quinidine and examined compounds were performed in DMSO. The 4X concentrated dilutions were prepared before the assays (0.04-100 µM). The enzymatic reactions were conducted in white polystyrene, flat-bottom Nunc™ MicroWell™ 96-Well Microplates (Thermo Scientific, Waltham, MA USA).

The CYPs, proluciferin and examined compounds (25 µl/well) were preincubated first for 5 min and next the NADPH Regeneration System was added (25 µl) to start the reaction. The final concentrations of KE and examined compounds were in range from 0.01 µM to 25 µM. The final concentrations of QD were from 0.001 to 10 µM. The control reactions for measuring the 100% of CYPs activity and minus-CYP negative control reactions for measure background luminescence were also prepared. The microplate was incubated in room temperature for 30 min (CYP3A4) or 45 min (CYP2D6). Finally, LDR was added (50 µl/well) and after 20 min of incubation in room temperature the luminescence signal was measured with a microplate reader (EnSpire) in luminescence mode. The signal produced by CYPs without the presence of compounds was considered as 100% of CYP activity. The IC_{50} values were calculated using GraphPad Prism 5 Software.

2.11. Assessment of cell membrane permeability

The assay was carried out by Eurofins Cerep (Celle L'Evescault, France) according to the service-provider’s protocol. Briefly, Caco-2 cells were seeded at 1×10^5 cells/cm^2 in 96-well Multiscreen™ plates (Millipore). Permeability assays were performed with the cells at days 21-25 post-seeding. The assay was performed in either the apical to basolateral A-B or B-A direction. The test compound was prepared at 10 µM in HBSS-HEPES (pH 7.4) with a final DMSO concentration of 1 %. The working solution was then centrifuged and the supernatant was added to the donor side. The assay plate was incubated at 37 °C with gentle shaking for 60 min or 40 min for the A-B or B-A assay, respectively. For assays tested in the presence of verapamil, 100 µM verapamil was included on both the A and B sides. Samples were aliquoted from the donor side at time zero and the end point, and from the receiver side at the end point. Propranolol (highly permeable), labetalol (moderately permeable), ranitidine (poorly permeable), and colchicine (P-glycoprotein substrate) are included in each assay as assay controls. Samples were analyzed on a Sciex triple quadrupole 5500 coupled to an Agilent Infinity II 1290 UHPLC.

Verapamil was used as PgP inhibitor – efflux B2A/A2B ratio higher than 2 suggests that the compound needs to be considered as PgP substrate. Results are presented in Supp. Table 3.

2.12. Assessment of compound PZKKN-94 mutagenicity

The study of PZKKN-94-induced genotoxicity was conducted by Covance (Harrogate,England) according to the service-provider's protocol and in compliance with the Good Laboratory Practice (GLP). The compound was tested at a concentration range of 0.05–50 mg/mL (5-5000 µg/plate). Mutagenicity was evaluated in five strains of Salmonella typhimurium (TA98, TA100, TA1535, TA1537 and TA102), in a single experiment, at the concentrations detailed previously, using triplicate plates without and with S-9 for test article, vehicle and positive controls. The assay was considered valid if the vehicle control counts fell within the laboratory's historical control ranges and the positive control chemicals induced increases in revertant numbers of ≥ 1.5 fold (in strain TA102), ≥ 2-fold (in strains TA98 and TA100) or ≥ 3-fold (in strains TA1535 and TA1537) the concurrent vehicle control confirming discrimination between different strains, and an active S-9 preparation. The test article was considered to be mutagenic if a concentration related increase in revertant numbers was ≥ 1.5-fold (in strain TA102), ≥ 2-fold (in strains TA98 or TA100) or ≥ 3-fold (in strains TA1535 or TA1537) the concurrent vehicle control values. Results are given in Supp. Table 4.

3. *In vivo* pharmacological properties of compound PZKKN-94

3.1. Drug treatment

PZKKN-94 was obtained following the protocol disclosed Section 1.1. All other compounds were provided by Sigma-Aldrich (Poznan, Poland) unless stated otherwise. Custom preparation of haloperidol (Polfa, Warszawa, Poland; ampoules of 5 mg/1 ml) was diluted to a concentration of 0.25 mg/kg with distilled water. 6-OHDA was dissolved in physiological saline containing 0.05% ascorbic acid. Apomorphine hydrochloride, benserazide hydrochloride, donepezil (Abcam, Cambridge, UK), phencyclidine, scopolamine and PZKKN-94 were dissolved in distilled water, and L-DOPA was suspended in propylene glycol (Chempur).

For rat's anesthesia, a mixture of ketamine (70 mg/kg, Bioketan, Biowet, Poland) and xylazine (6 mg/kg, Sedazin, Biowet, Poland) administered intraperitoneally (i.p.) was used.

Haloperidol was administered i.p. in a volume of 2 ml/kg. Apomorphine hydrochloride was injected subcutaneously (*sc*) in a volume of 2 ml/kg. For haloperidol-induced catalepsy, PZKKN-94 was administered i.p. in a volume of 2 ml/kg. For behavioral tests performed in 6-OHDA lesioned rats, benserazide and L-DOPA were injected i.p., whereas PZKKN-94 was administered per os using a gastric feeding tube, all in a volume of 2 ml/kg.

For cognition-related tests phencyclidine and scopolamine were solubilized in distilled water and were administered i.p. at the dose of 5 mg/kg (phencyclidine) and 1.25 mg/kg (scopolamine) 45 and 30 min before familiarization phase (T1), respectively. PZKKN-94 and donepezil were diluted in distilled water and administrated po 120 min before the familiarization phase (T1) of the novel object recognition (NOR) test or before the first discrimination phase of the attention set shifting task (ASST) (PZKKN-94). The drugs or vehicle (distilled water) were administered at a volume of 1 ml/kg of body weight.

For the forced swim test (FST) and open field test, PZKKN-94 was administered i.p., in a volume of 2 ml/kg, 60 min before respective test. Control rats were injected with the vehicle according to the same schedule.

3.2. Animals and ethical statements

3.2.1. Animals for pharmacokinetic assessment, FST and open field test (OF)

Male Wistar rats weighing 220-250 g were obtained from the accredited animal facility at Jagiellonian University Medical College (Kraków, Poland). The rats were kept in groups of 4 in Makrolon type III cages (dimensions 26.5 × 15 × 42 cm) in an environmentally controlled room (ambient temperature 22 ± 2°C; relative humidity 50-60%; air changes 15-20 times/h; light: dark cycle 12 h:12 h, lights on at 7:00). The animals had free access to standard laboratory food (LSM-B) and filtered water. Wood blocks, paper tubes and strips were used to enrich the environment. All experiments were performed between 9:00 and 14:00 in separate groups of animals by two observers unaware of the treatment applied. Animals were used only once. Immediately after the experiment, the animals were sacrificed by inhalation of carbon dioxide in the euthanasia chamber.

All experimental procedures were carried out in strict accordance with EU Directive 2010/63/EU and accordingly with Polish legal regulations (Dz.U. 2015 pos. 266) were approved by the II Local Ethics Commission at the Institute of Pharmacology (Krakow, Poland). The 3D rule was implemented in the study. A procedure for early, humane termination of the experiment was developed in the event of significant deterioration of an animal's health. The procedure was intended to be initiated when at least two of the following symptoms occurred: convulsions,

respiratory disturbance, movement disorder, loss of postural reflex, lack of water and/or food intake, muscle relaxation, or lack of touch response.

3.2.2. *Animals for the stepping, vibrissae and catalepsy tests*

Male Wistar Han rats (Charles River, Germany) with initial body weight of 270–300 g were used for the study. Rats were kept in an animal room (5 animals per cage) under standard laboratory conditions, with a temperature of 21 ± 1 °C and relative humidity of 50 ± 5%, under an artificial light/dark cycle (12/12 h), with free access to standard laboratory food and water. Wood blocks were used to enrich the environment. Behavioral testing was performed during the light phase of the light/dark cycle by two experienced investigators blind to the treatment group.

The experiments were carried out in accordance with the NIH Guide for the Care and Use of Laboratory Animals and were approved by the Local Ethics Committee for Animal Experiments at the Institute of Pharmacology (Krakow, Poland). All efforts were made to follow the 3R principles, using the minimal number of animals necessary to produce statistically reliable results, and minimizing animal suffering.

3.2.3. Animals for NOR test and ASST

Male Sprague-Dawley rats (Charles River, Germany) weighing 170–200 g on arrival were used for the scopolamine and phencyclidine NOR studies and ASST study. Young (10-weeks-old) and aged (19-months-old) Wistar Han rats were purchased at Janvier (France) and were used for the natural forgetting NOR study. Rats were group housed (4-5 rats per cage) in a temperature-controlled (21 ± 1 °C) and humidity-controlled (40–50%) colony room under a 12 h/12 h light/dark cycle (lights on at 06:00 am). For the ASST experiments, rats were mildly food deprived (17 g of food pellets per day) for at least one week before testing. For the NORT studies, rats had free access to food and water. Behavioral testing was performed during the light phase of the light/dark cycle.

The experiments were conducted in accordance with the NIH Guide for the Care and Use of Laboratory Animals and were approved by the Ethics Committee for Animal Experiments, Institute of Pharmacology (Krakow, Poland).

3.3. Experimental design, statistical analysis and requirements for studies using animals

We used male rats only a) to minimize effects of cycling on behavior, b) to gain insight into how present data compare with published results and c) due to the housing conditions, as females in our institutions' animal rooms must be housed separately from males.
Throughout the manuscript, $P < 0.05$ was considered significant.

3.3.1. *Estimation of required sample analysis*

Effect size for the **stepping test** was calculated based on previous data from our laboratory, published by Lenda et al. (2021). Based on the most conservative use case (comparing between within- and between-subject interactions) we obtained eta.sq = 0.71; thus f = 1.56. This is a very strong effect, not constrained by sample size: with the current design (2 within-subject measurements and 4 groups), even with alpha = 0.01 and power = 0.95, the estimated required total sample size is 12 (i.e. N = 3 per group). For **rotation test**, based on previous data published by Lenda et al. (2021), the relevant effect size is f = 0.49 (eta.sq = 0.194); with the design used (3 time points and 4 treatment groups), with alpha = 0.05 and power = 0.8 this corresponds to N = 16 (4/group). Effect size for **vibrissae test** was estimated based on Lenda et al. (2021) Fig. 3. Here, for the interaction between treatment and time point, we obtained eta.sq = 0.11, thus f = 0.35;

assuming alpha = 0.05 and power = 0.8 this translates to a required total sample size of N = 28 (7/group).

For 6-OHDA-induced **catalepsy test**, the post-hoc power analysis of data acquired from 32 animals, assuming alpha = 0.05 and power = 0.8, results in an estimated minimal detectable effect size f = 0.31 (i.e., within the range of “medium” effects, according to Cohen).

For **haloperidol-induced catalepsy test**, we used prior data by Konieczny and Lenda (2013), with effect size f = 0.74; with one-way design and three experimental groups, assuming alpha = 0.05 and power = 0.8, the required sample size N = 24 (i.e. 8/group) and for a more restrictive power = 0.95 the sample size required is N = 33 (11/group).

A novel object recognition (**NOR**) experiment from our laboratory, reported by Zajdel *et a*l. (2021), served to determine the required sample size of NOR experiments reported here. On its Fig. 4 we analyzed the effects of VEH, PCP and four drug + PCP combinations on Discrimination Index. With the help of https://www.psychometrica.de/effect_size.html the pooled SD = 0.136, total N = 44 and the number of groups = 6, Cohen’s effect size (f) = 1.68. We analyzed these data with G*Power software, and, assuming alpha = 0.001, power = 0.95 and 5 groups, the total N = 25 is required, suggesting that N = 5/group should reveal significant differences. See Fig. 4 of the present manuscript. With similar assumptions, and 8 groups (see Fig S16 and Fig S18 of the present ms), the total N = 32 required, suggesting N = 4/group.

The attentional set shifting test (**ASST**) experiment from our laboratory published as Drop et al. (2021) and Zajdel et al. (2021) served for required sample size analysis for the present manuscript’s Fig. 5. The pooled SD (data of ED phase) is 0.883, total N = 31, 3 groups, Cohen’s effect size (f) = 0.966. We analyzed these data with G*Power software, and, assuming alpha = 0.01, power = 0.95 and 4 groups, the total N = 32 is required, suggesting N = 8/group should reveal significant differences. See Fig S19 of the present manuscript.

The forced swim test (**FST**) experiment from our laboratory published as Grychowska et al. (2016) served to determine the sample size required for experiments (Supp. Fig. 11of the present manuscript). The pooled SD (data for immobility time) is 35,4, total N = 29, and the number of groups is 4, Cohen’s effect size (f) = 1,0867. We analyzed these data with the G*Power software, and assuming alpha = 0.01, power = 0.95, the total N = 28 is required, suggesting that N = 7 per group should reveal significant differences. See Supp. Fig. 11of the present manuscript.

The **motor activity** of compound PZKKN-94 was tested in two experiments carried out on different days. In the *first* experiment, the effect of the compound administered at doses of 0.1, 0.3 and 1 mg/kg was tested, and since the highest activity of PZKKN-94 was shown at the lowest dose of 0.1 mg/kg, the effect of the dose of 0.03 mg/kg was tested in the *next* experiment. The results for the control groups were similar (the mean immobility time in the first experiment was 187.5 ± 12.3 seconds, and in the second was 183.3 ± 8.0 sec.), therefore, the results obtained in control groups were pooled for statistical analysis.

3.3.2. *Randomization, blinding and exclusion criteria*

For **haloperidol-treated rats**, naïve animals were randomly assigned to one of the three treatment groups using computer-generated random numbers (Excel), and coded.

For **6-OHDA-lesioned** rats, only rats with severe lesions were included in the behavioral studies, which was confirmed by the apomorphine-induced rotations. Rats that passed the apomorphine rotation test (> 100 contralateral turns/h) were allocated to individual groups, balanced with respect to their rotational score. Then, groups were randomly assigned to one of the four treatments using computer-generated random numbers, and coded.

In general, rats were randomly assigned to treatment groups as follows. Each rat was assigned a unique integer and then, using a freeware online random number generator, subsequent numbers were drawn to allocate animals to control and experimental groups.

All measurements were performed by investigators **blinded** to the treatment received by rats. The experimental groups were coded, and data analysis was performed by an investigator who

did not participate in the administration of the compounds and measurements. Novel object recognition (NOR) test was video-recorded using AnyMaze(R) software, and also blindly analyzed off-line.

All experiments were arranged so that animals from all experimental groups were tested in each session.

3.4. Methods

3.4.1. Pharmacokinetic profile of PZKKN-94

Rats were fasted for 12 h (water was available at libitum) before administration and fed 4 h post-dose. Rats received PZKKN94 either intravenously (3 mg/kg) or per oral gavage (10 mg/kg). The blood was collected at each of eight time points (5 min, 15 min, 30 min as well as 1, 2, 4, 7 and 12 h) for animals after intravenous administration (n = 7). Animals receiving a dose of 10 mg/kg (n = 5/time-point) per oral gavage were sacrificed (by inhalation of isoflurane and decapitation) at each of time points (15 min, 30 min as well as 1, 2, 4, 7, 12 and 24 h). Blood and brain tissue was collected.

For all groups, blood was collected into tubes containing the anticoagulant K_2EDTA and centrifuged (3000 × g, 15 min, 4 °C) to obtain plasma (stored at −20 °C). Brain samples were homogenized in water (3 mL water per 1 g of brain tissue) and stored at −80 °C until analysis.
Acetonitrile (150 μL) spiked with internal standard was added to 50 μL of each plasma homogenate or brain homogenate, respectively. The mixture was shaken vigorously for 1 min and then centrifuged (10621 × g, 4 min, room temperature). Supernatants were injected into a high-performance liquid chromatography system (Agilent Technologies, Santa Clara, CA, USA) with a Zorbax SB C-18 column (50 cm × 2.1 cm; 1.8 μm; Agilent Technologies). The mobile phase was ammonium formate (20 mM, pH 3.0) and acetonitrile with 0.1% formic acid. The gradient chromatographic separation used a flow rate of 0.3 mL/min for 8 min at a nominal temperature of 50 °C. Signal acquisition was undertaken for 8 min using of a triple quadrupole mass spectrometer (Sciex QTrap 4500; Sciex) with electrospray ionization (3500 V, 400 °C). Multiple-reaction monitoring was applied to detect the following transitions of the m/z ratio: 390.2 to 175.4 and 380.2 to 202.2 for PZKKN-94; 447.5 to 296.3 and 447.5 to 362.3 for internal standard. The drug concentration was calculated from the ratio of the peak area response relative to the internal standard using a standard curve. The pharmacokinetic parameters were analyzed using Phoenix® WinNonlin® version 8.3 (Certara, Inc., Princeton, NJ, USA).

3.4.2. 6-OHDA-induced lesion

Unilateral 6-OHDA-induced lesion of the rat medial forebrain bundle is a standard experimental PD model to produce a total degeneration of the dopaminergic system resulting in significant impairment of motor functions on the side contralateral to the lesion.

Two weeks after lesion, the number of the apomorphine-induced contralateral rotations (which result from activation of supersensitive dopaminergic D_2 receptors in the denervated striatum by apomorphine) was approx. 200 turns/h in the individual groups which corresponds to the total lesion of DA system (Konieczny et al., 2014) (99.6% decrease in DA level in the lesioned striatum which was confirmed in the present study by the HPLC analysis; data not shown).

3.4.3. Rotational behavior

Rotational behavior was recorded in automated rotameters (Panlab), immediately following injection of apomorphine or L-DOPA (Konieczny et al., 2014). The number of complete 360° rotations in both directions was analyzed for 60 or 120 min, for apomorphine and L-DOPA,

respectively, with 10 min intervals. Since rats showed a negligible number of ipsilateral rotations, only contralateral rotations were included in further analysis.

3.4.4. Novel object recognition test

Rats were tested in a dimly lit (25 Lux) open field made of dull grey plastic (length × width × height: 66 × 56 × 30 cm) and habituated to the arena (without any objects) for 5 min 24 h before testing (Potasiewicz et al., 2020). The test consisted of two 3-min trials separated by an inter-trial interval (ITI) of 24 h or 60 min in the scopolamine and PCP studies. During the first trial (familiarization, T1), two identical objects (A1 and A2) were presented in opposite corners, approximately 10 cm from the walls of the open field. In the second trial (retention, T2), one of the objects was replaced by a novel one (A = familiar, and B = novel). The objects used were a glass bulb filled with gravel and a plastic bottle filled with sand. The height of the objects was comparable (~12 cm), and they were heavy enough not to be displaced by the animals. Half of the animals from each group received the glass bulb as a novel object, and the other half received the plastic bottle. The location of the novel object in the recognition trial was randomly assigned for each rat. Exploration of an object was defined by looking, licking, sniffing, or touching the object while sniffing but not leaning against, standing, or sitting on the object. Any rat that spent less than 5 s exploring the two objects within 3 min of T1 or T2 was eliminated from the study. The behavior was recorded by the camera placed above the arena and connected to the Any-maze® tracking system (Stoelting Co., Illinois, USA). The experimenter, blinded to the treatment conditions, manually assessed the exploration time. Based on the exploration time (E) of the two objects in the retention trial, a discrimination index (DI) was calculated as $(E_B–E_A)/(E_A+E_B)$.

3.4.5. Attentional set shifting task

Testing was conducted in a Plexiglas apparatus (length × width × height: 38 x 38 x 17 cm) with the grid floor and wall dividing half of the length of the cage into two sections. During testing, one ceramic digging pot (internal diameter of 10.5 cm and a depth of 4 cm) was placed in each section. Each pot was defined by a pair of cues along with two stimulus dimensions. To mark each pot with a distinct odor, 5 µL of a flavoring essence (Dr. Oetker®, Poland or The Body Shop, UK) was applied to a piece of blotting paper fixed to the external rim of the pot immediately prior to use. A different pot was used for each combination of digging medium and odor; only one odor was ever applied to a given pot. The bait (one-half of a Honey Nut Cheerio, Nestle®) was placed at the bottom of the “positive” pot and buried in the digging medium. A small amount of powdered Cheerio was added to the digging media to prevent the rat from trying to detect the buried reward by its smell. As described previously (Potasiewicz et al., 2018; Popik et al., 2020), the procedure lasted 3 days for each rat.

Day 1, habituation: rats were habituated to the testing area and trained to dig in the pots filled with sawdust to retrieve the food reward. Rats were transported from the housing facility to the testing room, where they were presented with one unscented pot (filled with several pieces of Cheerios) in their home cages. After the rats had eaten the Cheerio from the home cage pot, they were placed in the apparatus and given three trials to retrieve the reward from both of the sawdust-filled baited pots. With each exposure, the bait was covered with an increasing amount of sawdust. The following pairs of exemplars were used: Pair 1: odor: spicy *vs.* vanilla, medium: cotton wool *vs.* crumpled tissue; Pair 2: odor: lemon *vs*. almond, medium: shredded pipette tips *vs.* wooden sticks; and Pair 3: odor: rum *vs*. cream, medium: shredded papers *vs.* silk. The exemplars were always presented in pairs and varied so that only one animal within each treatment group received the same combination. In addition, the assignment of each exemplar in a pair as being positive or negative at a given phase and the left-right positioning of the pots in the test apparatus on each trial were randomized.

Day 2, training: rats were trained on a series of simple discriminations (SD) to a criterion of six consecutive correct trials. For these trials, rats had to learn to associate the food reward with an odor cue (e.g., arrack *vs.* orange, both pots filled with sawdust) and/or a digging medium (e.g., plastic balls *vs.* pebbles, no odor). All rats were trained using the same pairs of stimuli. The positive and negative cues for each rat were presented randomly and equally. These training stimuli were not used again in later testing trials.

Day 3, testing: rats performed a series of discriminations in a single test session. The first four trials at the beginning of each discrimination phase were discovery trials, during which the animals were allowed to dig in both bowls. The first trial of the discovery period was not included in the six criterion trials. In the subsequent trials, each incorrect choice was recorded as an error. Digging was defined as any distinct displacement of the digging media with either the paw or the nose; the rat could investigate a digging pot by sniffing or touching it without displacing material. Testing was continued at each phase until the rat reached the criterion of six consecutive correct trials, after which testing proceeded to the next phase. In the simple discrimination involving only one stimulus dimension, the pots differed along one of two dimensions (e.g., digging medium). For the compound discrimination (CD), the second (irrelevant) dimension (i.e., odor) was introduced, but the correct and incorrect exemplars of the relevant dimension remained constant. For the reversal of this discrimination (Rev 1), the exemplars and relevant dimensions were unchanged, but the previously correct exemplar was now incorrect and vice versa. The intra-dimensional (ID) shift was then presented, comprising new exemplars of both the relevant and irrelevant dimensions, with the relevant dimension remaining the same as previously. The ID discrimination was then reversed (Rev 2) so that the formerly positive exemplar became the negative one. For the extra-dimensional (ED) shift, a new pair of exemplars was again introduced, but a relevant dimension was also changed this time. Finally, the last phase was the reversal (Rev 3) of the ED discrimination.

The following pairs of exemplars were used: Pair 1: odor: spicy *vs*. vanilla, medium: cotton wool *vs.* crumpled tissue; Pair 2: odor: lemon *vs.* almond, medium: shredded pipette tips *vs.* wooden sticks; and Pair 3: odor: rum *vs.* cream, medium: shredded papers *vs*. silk. The exemplars were always presented in pairs and varied so that only one animal within each treatment group received the same combination. In addition, the assignment of each exemplar in a pair as being positive or negative at a given phase and the left-right positioning of the pots in the test apparatus on each trial were randomized.

3.4.6. Open Field Test

This test was carried out in a dark room using the Motor Monitor System (Campden Instruments, Ltd., UK) consisting of two Smart Frame Open Field stations (dimensions 40 × 40 × 38 cm) with 16 × 16 beams, located in sound-attenuating chambers and connected to PC software by control chassis. Each animal injected with vehicles or the tested compound was placed in the center of the station. The total distance covered by the animal in 5 min was recorded by the automated Motor Monitor System (Supp. Table 5).

4. Results

4.1. 6-OHDA induces motor deficits

"Pre-lesion" tests showed no preference in using both forelimbs and no catalepsy in rats (Supp. Fig. 9). Three weeks after lesion, all rats which passed the apomorphine test (N = 32) had significant deficits in performing the movement using the impaired forelimb in the stepping test, especially when they were moved in the forehand direction (decrease to 13.9%, paired Student's t-test: $t(31) = 23{,}04$, $P < 0.05$) (Supp. Fig. 9A). Deficits were weaker (decrease to 42.7%) when animals were moved in the backhand direction (data not shown). Greater disability in performing the movement using the impaired forelimb was observed in the vibrissae test (Supp. Fig. 9B) in which the response of the impaired forelimb was reduced to 4.0% (Wilcoxon test: Z-score = 4.94, $P < 0.05$). Unilateral 6-OHDA administration into the MFB also produced moderate catalepsy (97.1 s ± 10.65) in rats (Wilcoxon test: Z-score = 4.86; $P < 0.05$) (Supp. Fig. 9C).

4.2. PZKKN-94 does not enhance catalepsy produced by 6-OHDA lesion

Evaluation of the data from rats treated acutely with compound PZKKN-94 by one-way ANOVA for repeated measures revealed only a significant effect of time ($F[1,28] = 53{,}94$, $P < 0.05$). Accordingly, descent latency gradually rose with testing in all treatment groups (Duncan test). This finding is in line with other reports showing increased duration of catalepsy after repeated testing (Hillegaart et al., 1987). None of doses of compound PZKKN-94 influenced the lesion-induced catalepsy when compared to the solvent group (Supp. Fig. 10).

An anticataleptic effect of L-DOPA was seen already after the first dose and persisted during chronic treatment (ON phase) (one-way ANOVA for repeated measures: time effect: $F[3,81] = 57.62$, $P < 0.05$). Compound PZKKN-94 did not affect the anticataleptic effect of L-DOPA in any of the measurements. In OFF phase, the anticataleptic effect of L-DOPA was no longer visible, and combined treatment did not significantly influence L-DOPA effect.

4.3. Chronic administration of PZKKN-94 does not reduce therapeutic effect of L-DOPA in the rotational test

Both L-DOPA alone, and combined with PZKKN-94 produced strong, contralateral rotations already after acute administration, and their number increased after chronic treatment (one-way ANOVA for repeated measures: effect of time $F[2,54] = 17.01$, $P < 0.05$). No dose of PZKKN-94 significantly affected the L-DOPA-induced rotations (Supp. Fig. 10).

4.4. PZKKN-94 displays antidepressant-like properties

The compound PZKKN-94 showed antidepressant-like activity in Wistar rats without affecting the motor activity of the animals. In modified forced swim test, the compound administered at a dose of 0.1 mg/kg significantly shortened the immobility time by 60% and increased climbing time by 191% while at a dose of 0.3 mg/kg decreased immobility by 33% and extended climbing by 77% ($F(4,39) = 18.67$, $P < 0.0001$ for immobility time and $F(4,39) = 17.17$, $P < 0.0001$ for climbing time) (Supp. Fig. 11). Compound PZKKN-94 at active antidepressant doses did not change the spontaneous locomotor activity of rats in the open field test (Supp. Table 5), which suggests that observed effect is specific. The modified FST allows for a preliminary differentiation of the mechanisms of action of active substances in this test. Increase in noradrenergic and/or dopaminergic transmission leads to an increase in climbing time, while serotoninergic duration of swimming (Detke et al., 1995; Rénéric and Lucki, 1998). Hence, it can be concluded that rather noradrenergic and/or dopaminergic, but not serotoninergic system, participates in the antidepressant activity of compound PZKKN-94.

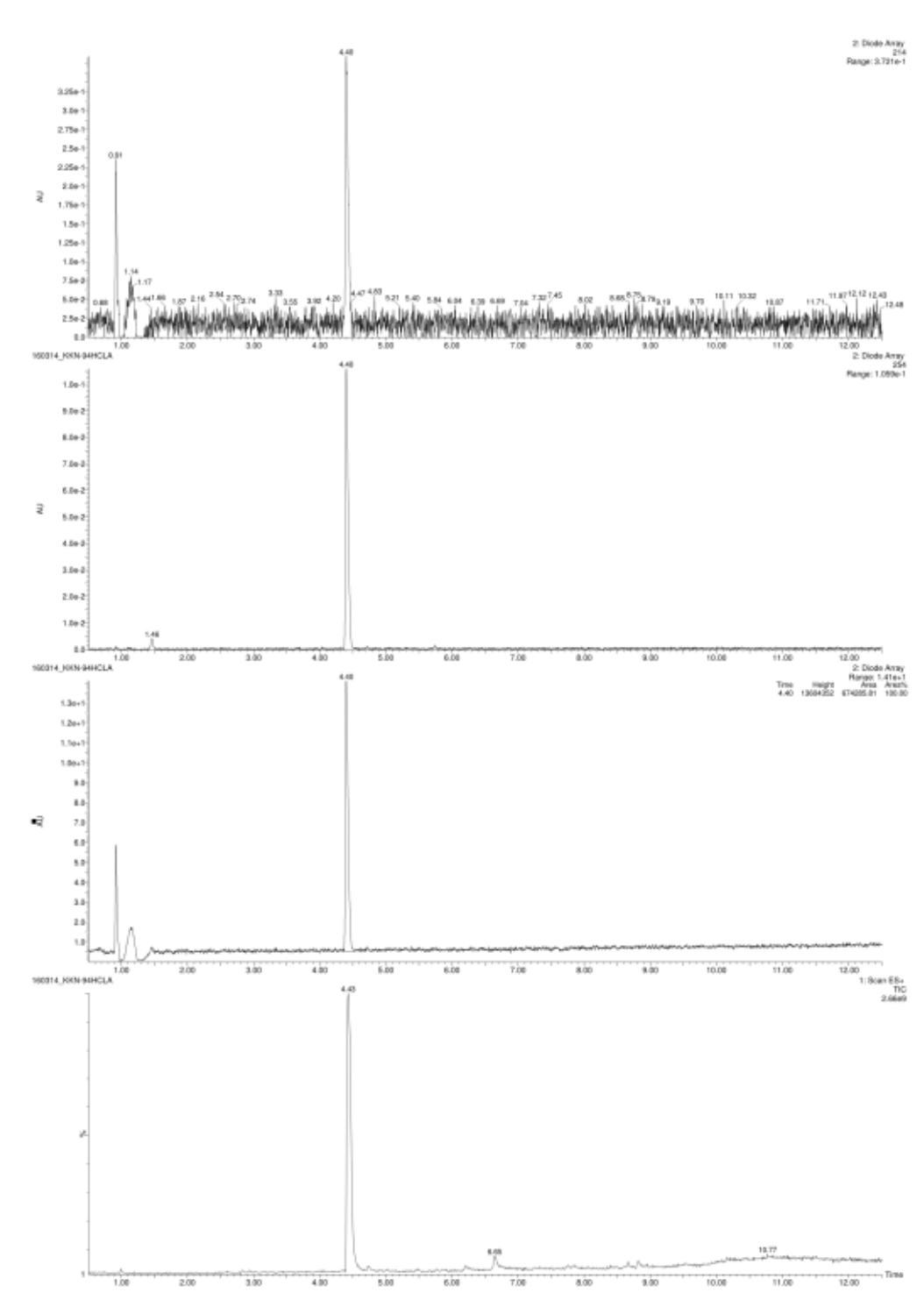

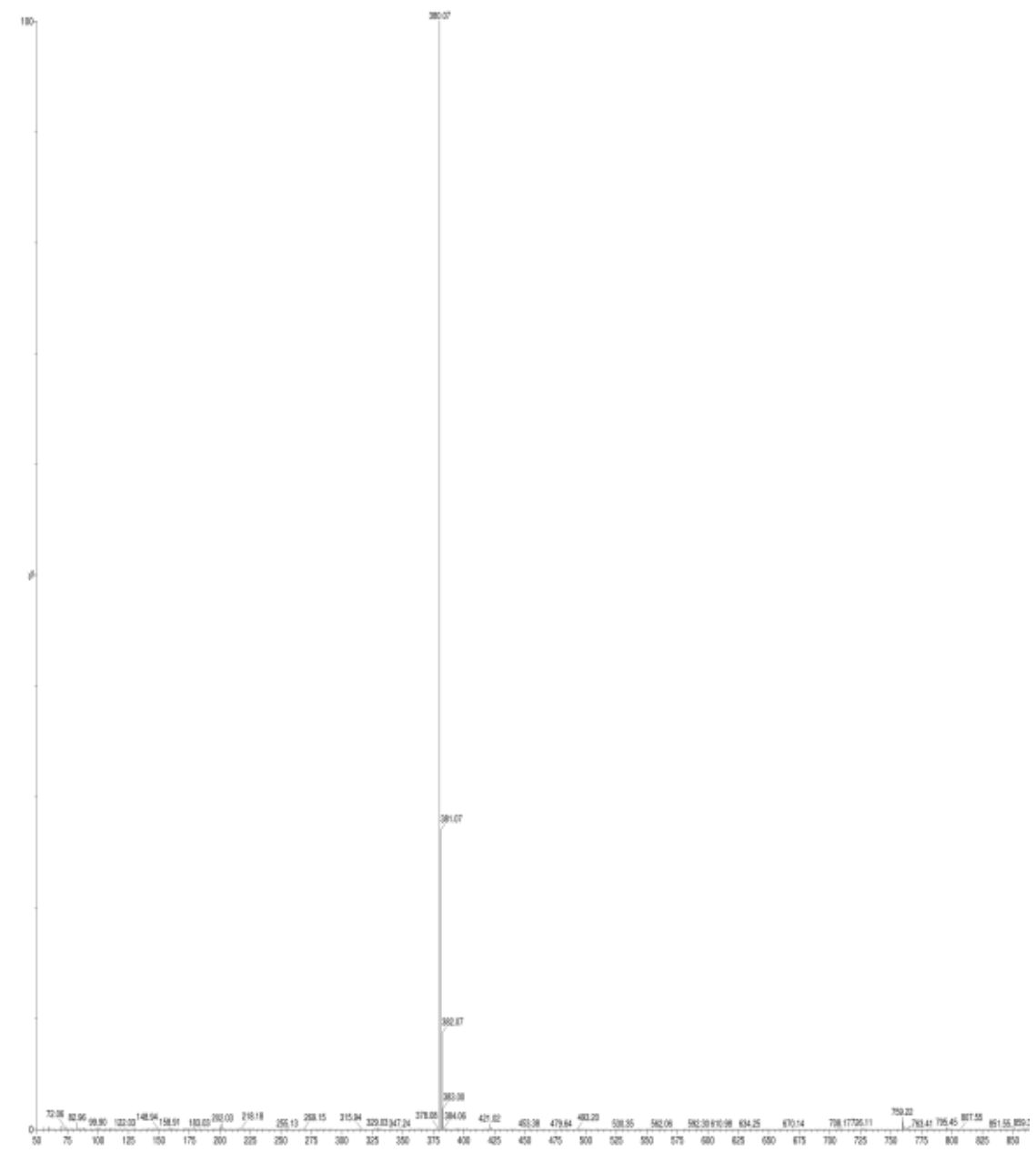

Supplemental Figure 1. UPLC chart and MS spectra for PZKKN-94. A. UPLC chart for PZKKN-94. B. MS spectra for PZKKN-94.

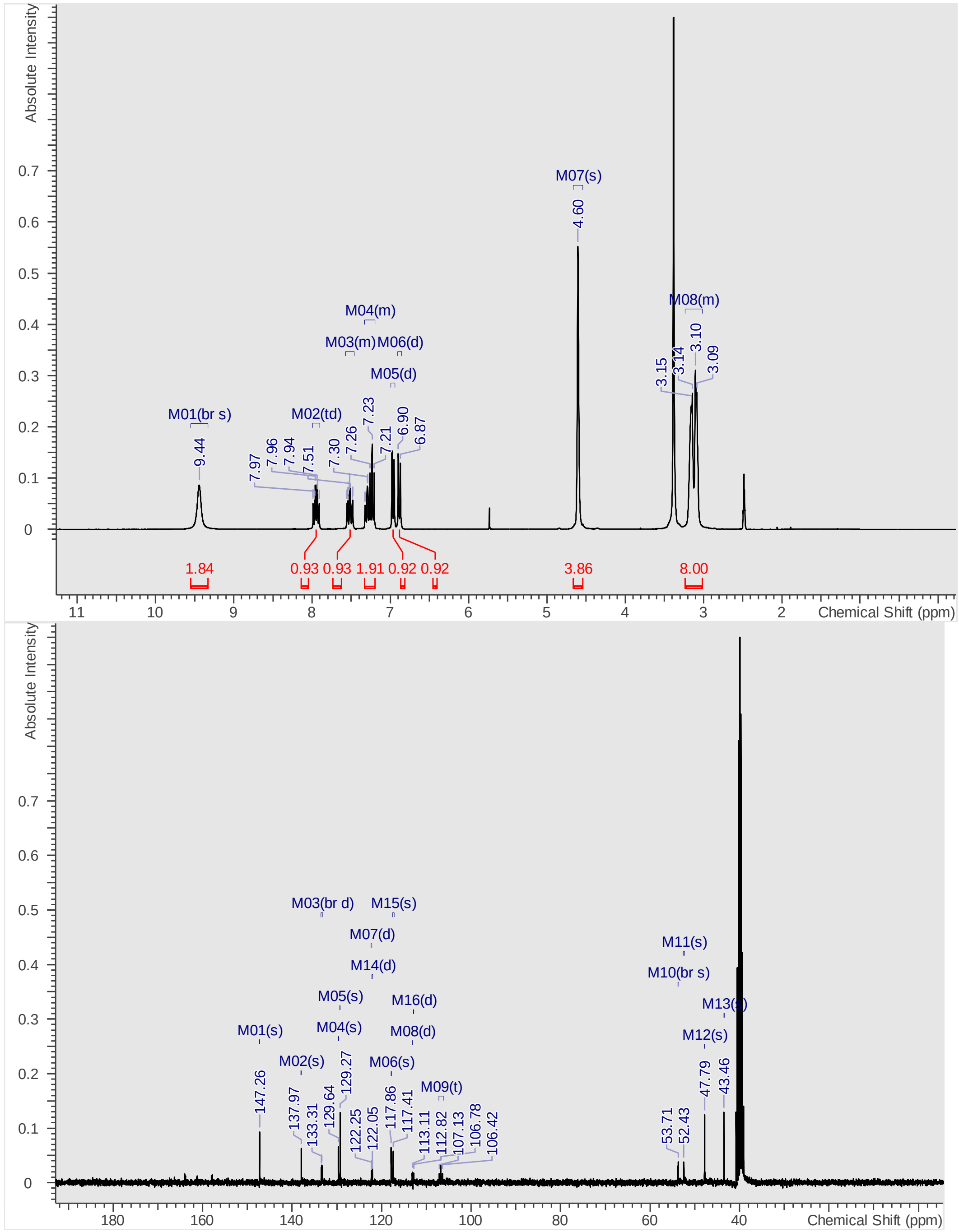

Supplemental Figure 2. ^{1}H NMR and ^{13}C NMR spectra for PZKKN-94. A. ^{1}H NMR spectra for PZKKN-94 (300 MHz, DMSO-d_6). B. ^{13}C NMR spectra for PZKKN-94 (75 MHz, DMSO-d_6).

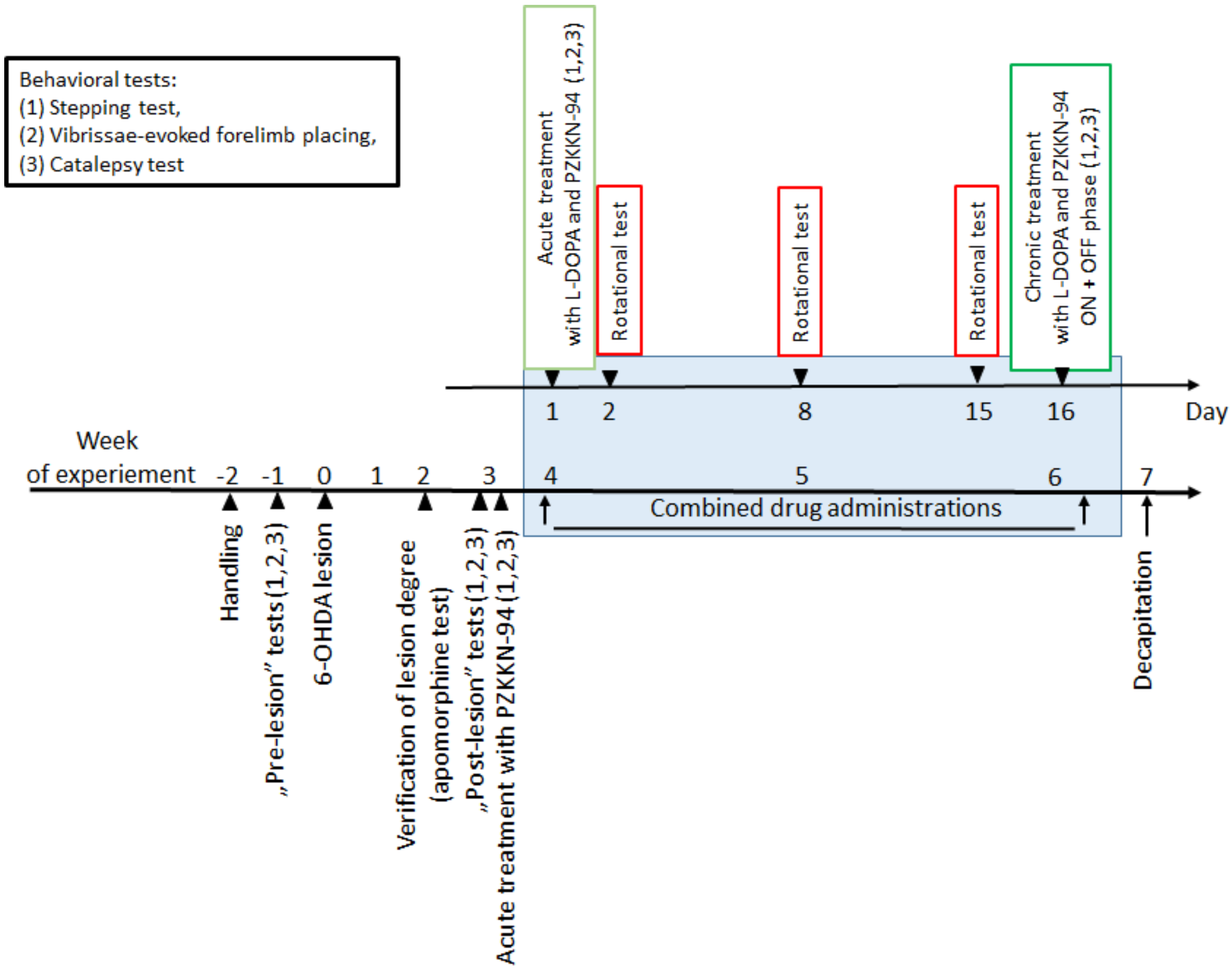

Supplemental Figure 3. Experimental schedule of testing rats lesioned unilaterally with 6-OHDA.

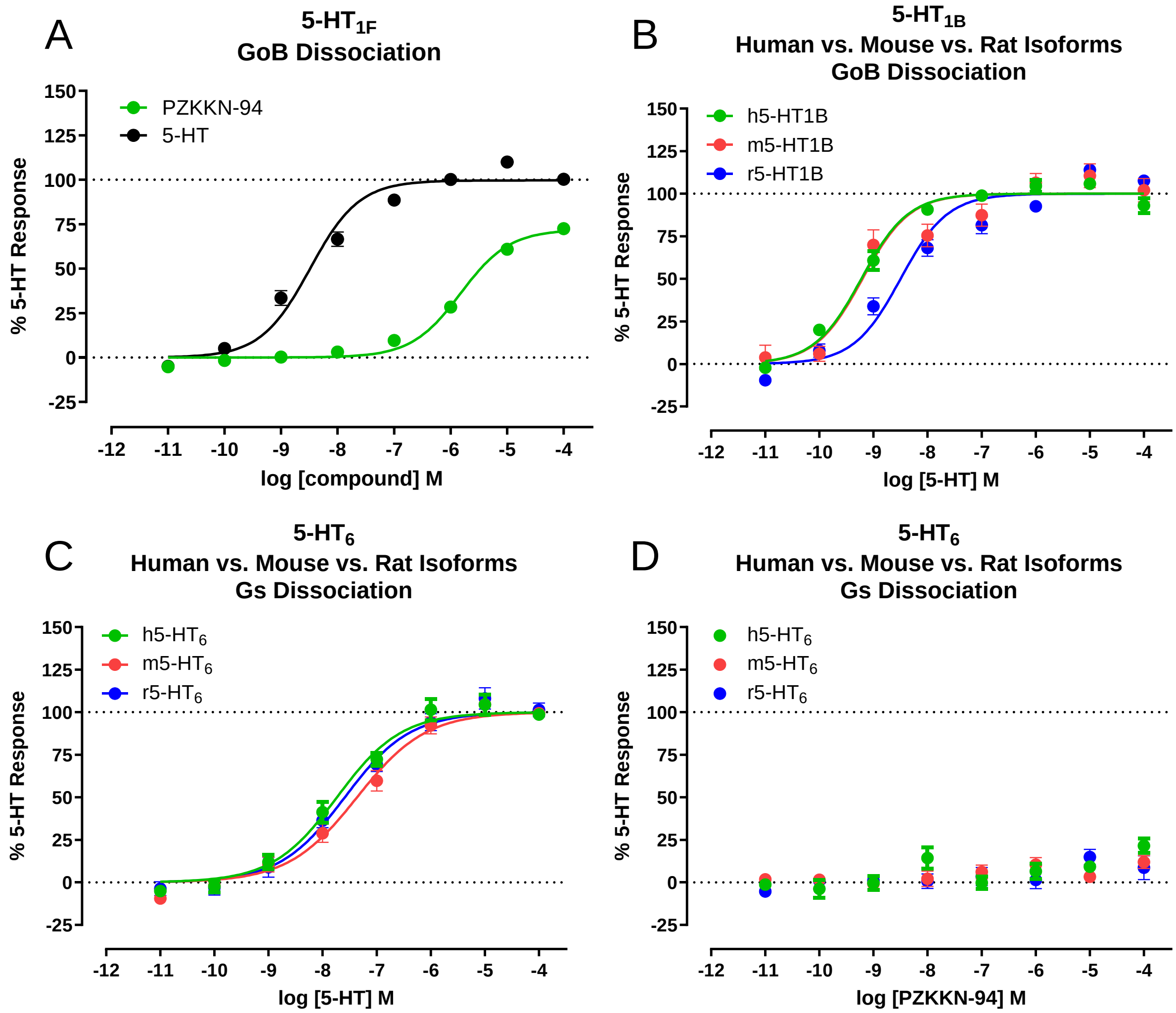

Supplemental Figure 4. A) Effect of PZKKN-94 on human 5-HT_{1F} G (oB) protein dissociation activity as measured by BRET. PZKNN-94 (green; EC_{50} = 1490 nM E_{MAX} = 72.0% of 5-HT response) compared to 5-HT (black; EC_{50} = 3.2 nM). Data represent mean ± SEM from five independent experiments (N = 5) performed in duplicate. **B**) Effect of 5-HT activity at human and rodent 5-HT_{1B} G (oB) protein dissociation as measured by BRET. human (h5-HT_{1B}; green; EC_{50} = 0.60 nM), mouse (m5-HT_{1B}; red; EC_{50} = 0.63 nM), and rat (r5-HT_{1B}; blue; EC_{50} = 3.2 nM). Data represent mean ± SEM from three independent experiments (N = 3) performed in duplicate. **C**) Effect of 5-HT activity at human and rodent 5-HT_6 Gs protein dissociation as measured by BRET. 5-HT activity at human (h5-HT_6; green; EC_{50} = 18.4 nM), mouse (m5-HT_6; red; EC_{50} = 43.3 nM), and rat (r5-HT_6;blue; EC_{50} = 25.7 nM). Data represent mean ± SEM from three independent experiments (N = 3) performed in duplicate. **D**) Effect of PZKKN-94 agonist activity at human and rodent 5-HT_6 Gs protein dissociation as measured by BRET. No agonist activity detected for PZKKN-94 activity at human (h5-HT_6; green), mouse (m5-HT_{1B}; red), and rat (r5-HT_{1B};blue). Data represent mean ± SEM from three independent experiments (N = 3) performed in duplicate.

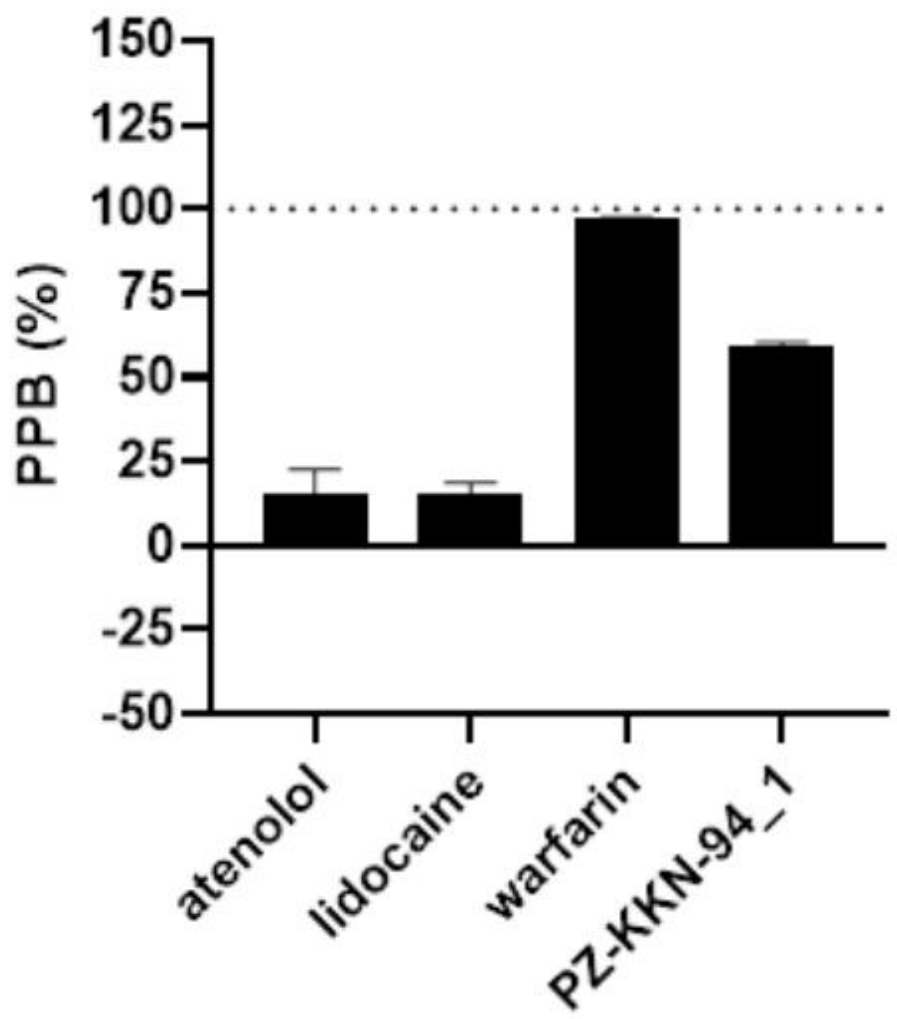

	PPB (%)		
	Mean	SD	N
atenolol	15.057	7.488	3
lidocaine	15.236	3.438	3
warfarin	97.286	0.139	3
PZ-KKN-94_1	58.882	1.556	6

Supplemental Figure 5. Rat plasma binding protein for PZKKN-94, atenolol, lidocaine and warfarin, used as references. Data are presented as mean ± SD (N = 6).

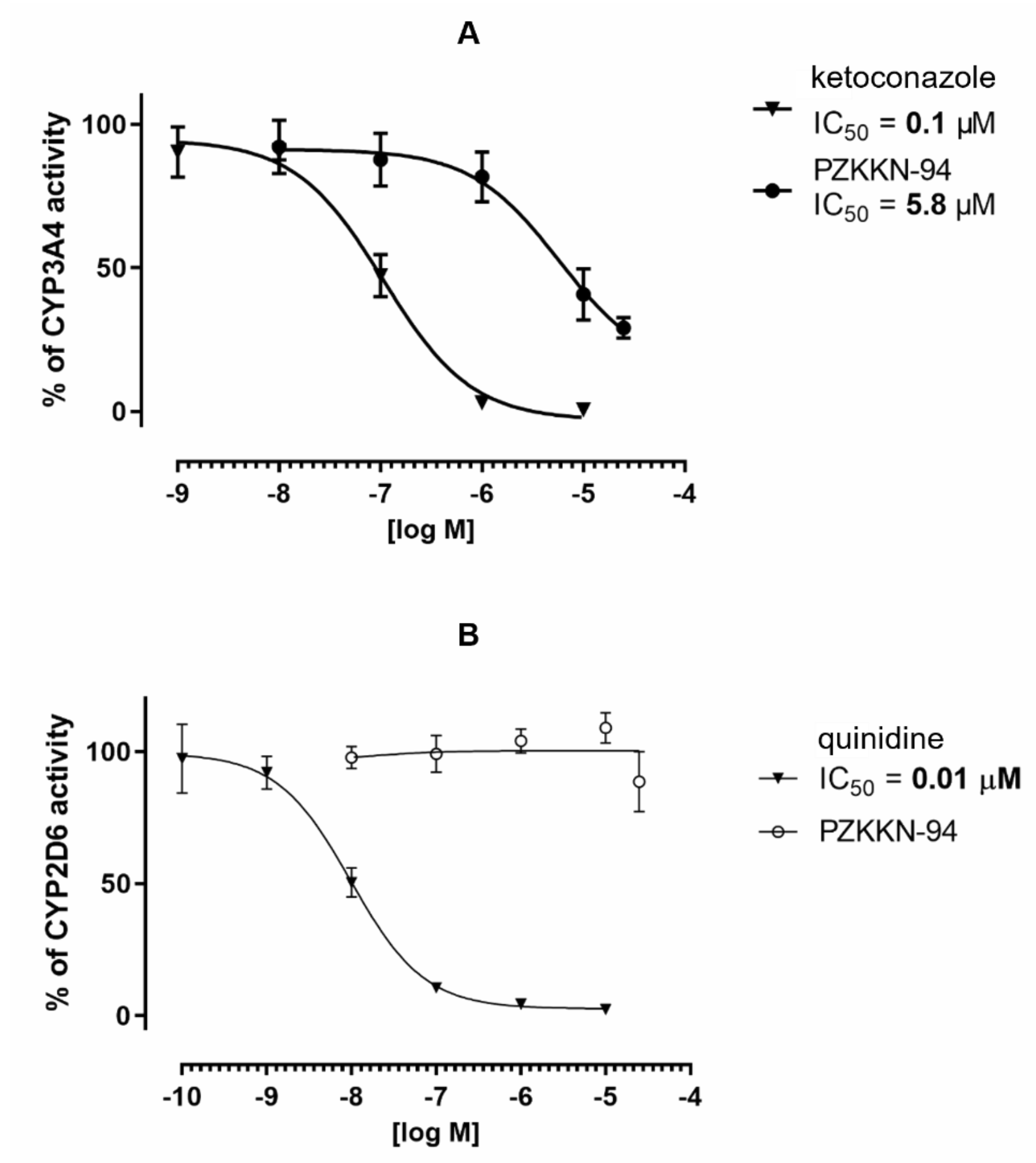

Supplemental Figure 6. The impact of PZKKN-94 on *in vitro* CYP3A4 and CYP2D6 activity. A) The impact of PZKKN-94 and ketoconazole, used as reference on *in vitro* CYP3A4 activity. Data are presented as mean ± SD (N = 5). B) The impact of PZKKN-94 and quinidine, used as reference, on *in vitro* CYP2D6 activity. Data are presented as mean ± SD (N = 5).

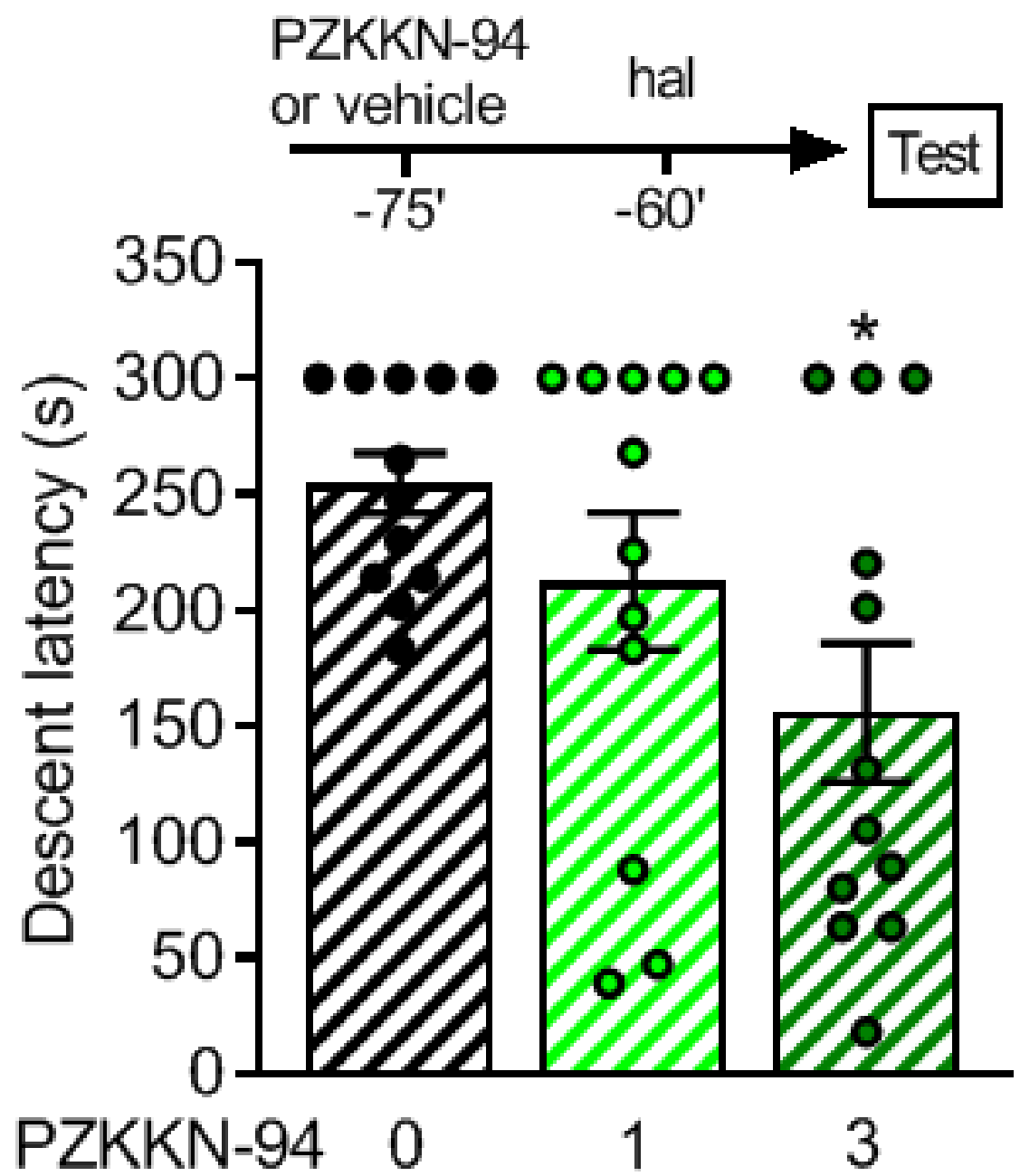

Supplemental Figure 7. PZKKN-94 decreases haloperidol-induced catalepsy in rats. Results represent the mean descent latency ± SEM. N = 12 rats per group. *P < 0.05 *vs*. haloperidol 0.25 mg/kg.

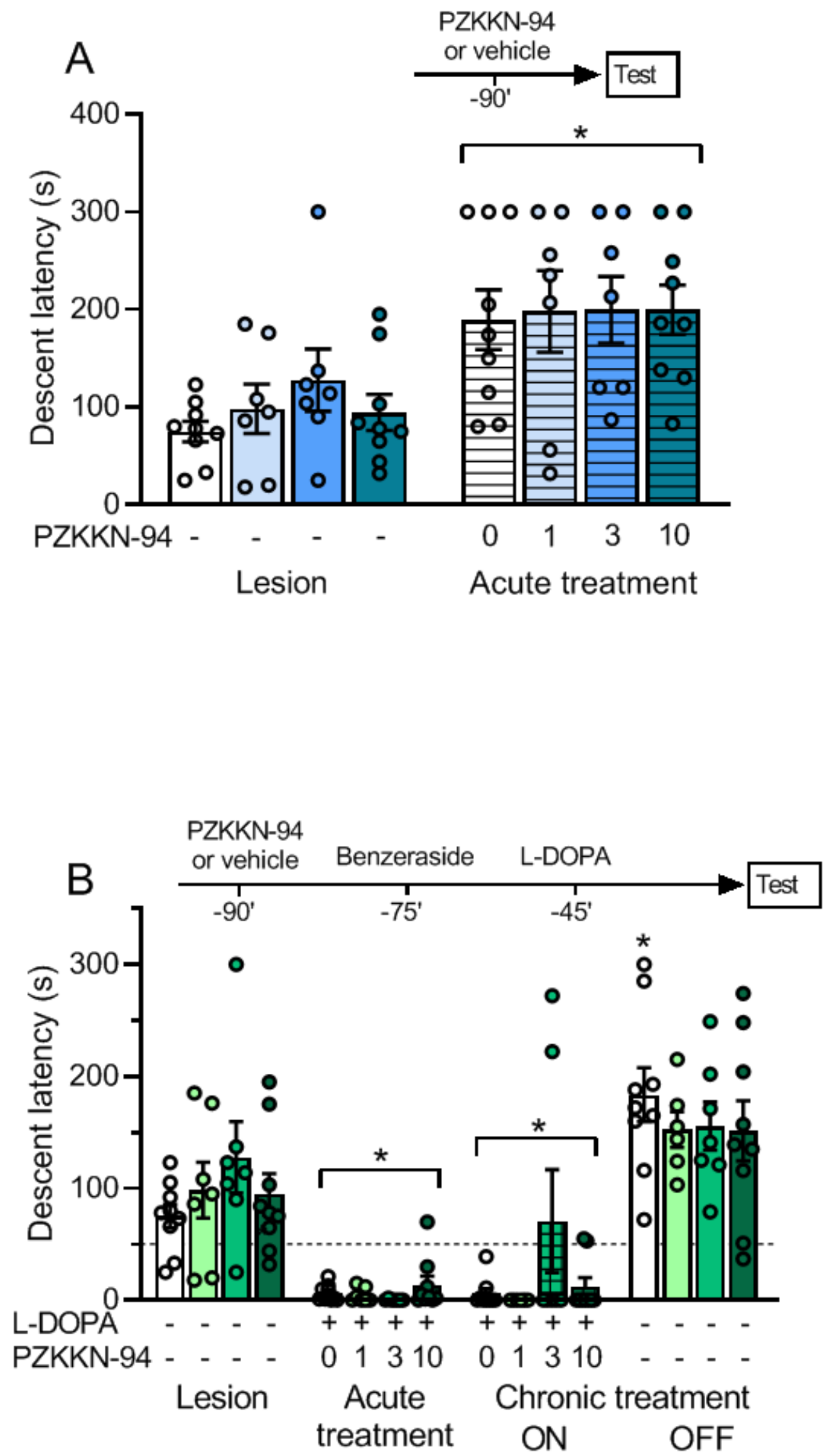

Supplemental Figure 8. The effect of PZKKN-94 and L-DOPA on performance of the catalepsy test in unilaterally 6-OHDA-lesioned rats. Results represent the mean descent latency ± SEM.(A) Acute PZKKN-94 does not affect catalepsy induced by 6-OHDA lesion. N = 7–9 rats per group. *P < 0.05 *vs.* Lesion. (B) After combined treatment, PZKKN-96 does not affect the therapeutic effect of L-DOPA. N = 6–9 rats per group. *P < 0.05 *vs.* Lesion.

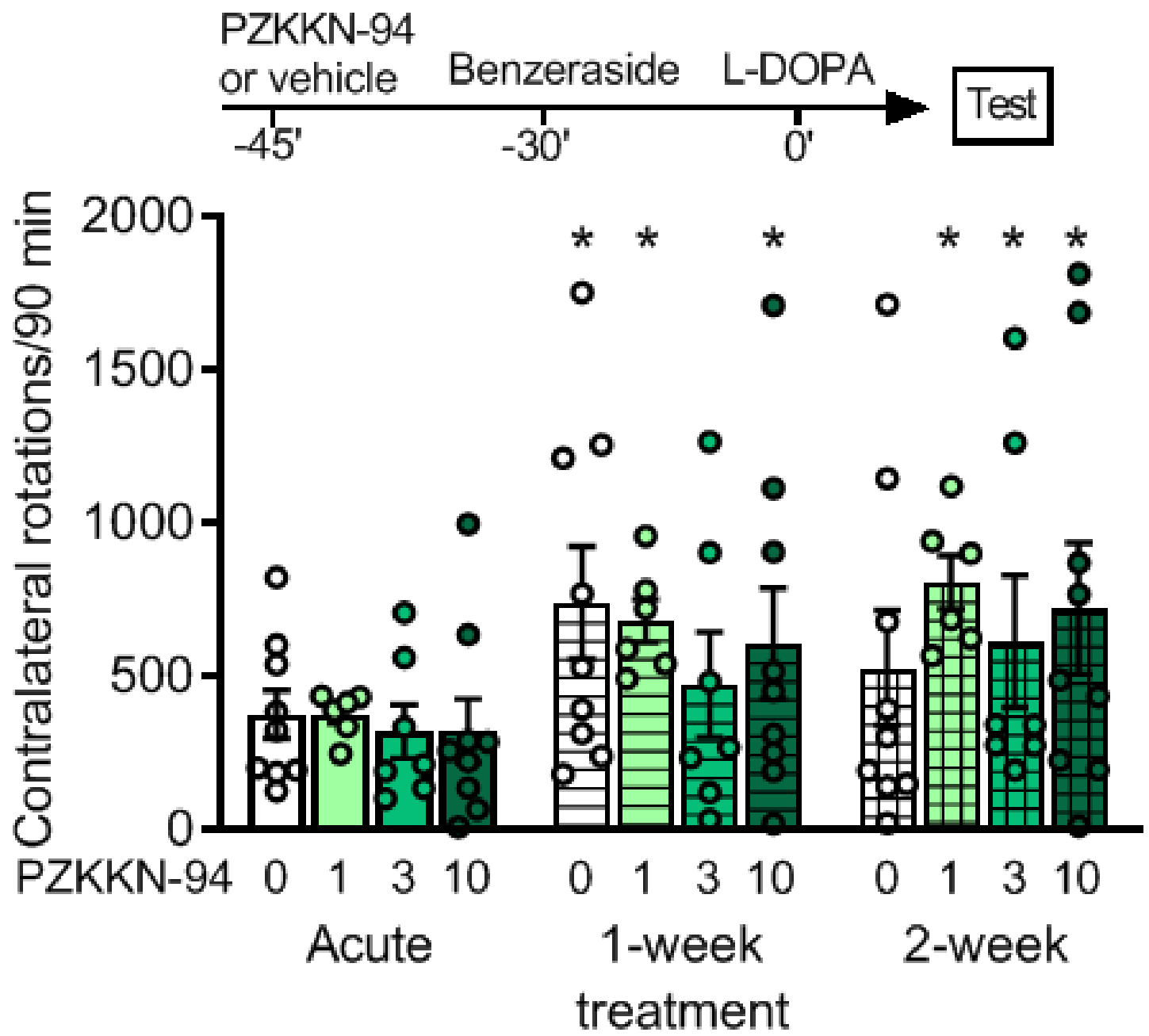

Supplemental Figure 9. PZKKN-94 does not affect L-DOPA-induced contralateral rotations in the unilaterally 6-OHDA-lesioned rats suggesting no influence on the therapeutic effect of L-DOPA. Results represent the mean number of contralateral rotations/90 min ± SEM. N = 6–9 rats per group. *P < 0.05 *vs.* Acute.

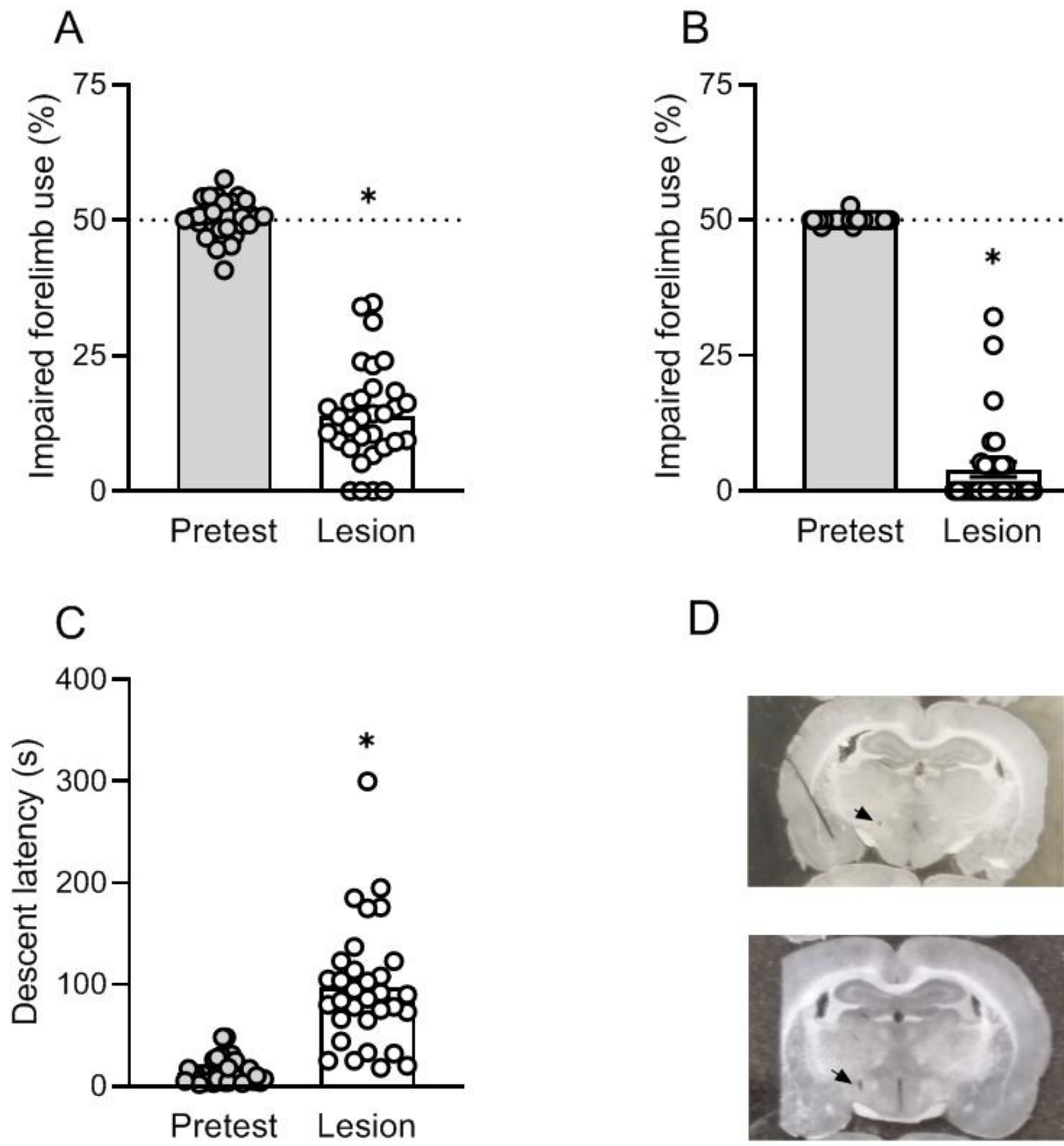

Supplemental Figure 10. The motor deficits produced by 6-OHDA three weeks after lesion. Results are presented as a mean ± SEM. (A) Stepping test. *P < 0.05 *vs*. Pretest. (B) Vibrissae test. *P < 0.05 *vs.* Pretest. (C) Catalepsy. *P < 0.05 *vs*. Pretest. N = 32 rats. The cohort contains all rats that passed the apomorphine rotation test two weeks after 6-OHDA lesion and were then allocated into one of the four treatment groups (Details in the *Methods*). (D) Typical examples of the cannula tip position (black arrowheads) in the medial forebrain bundle on the coronal brain section from two rats.

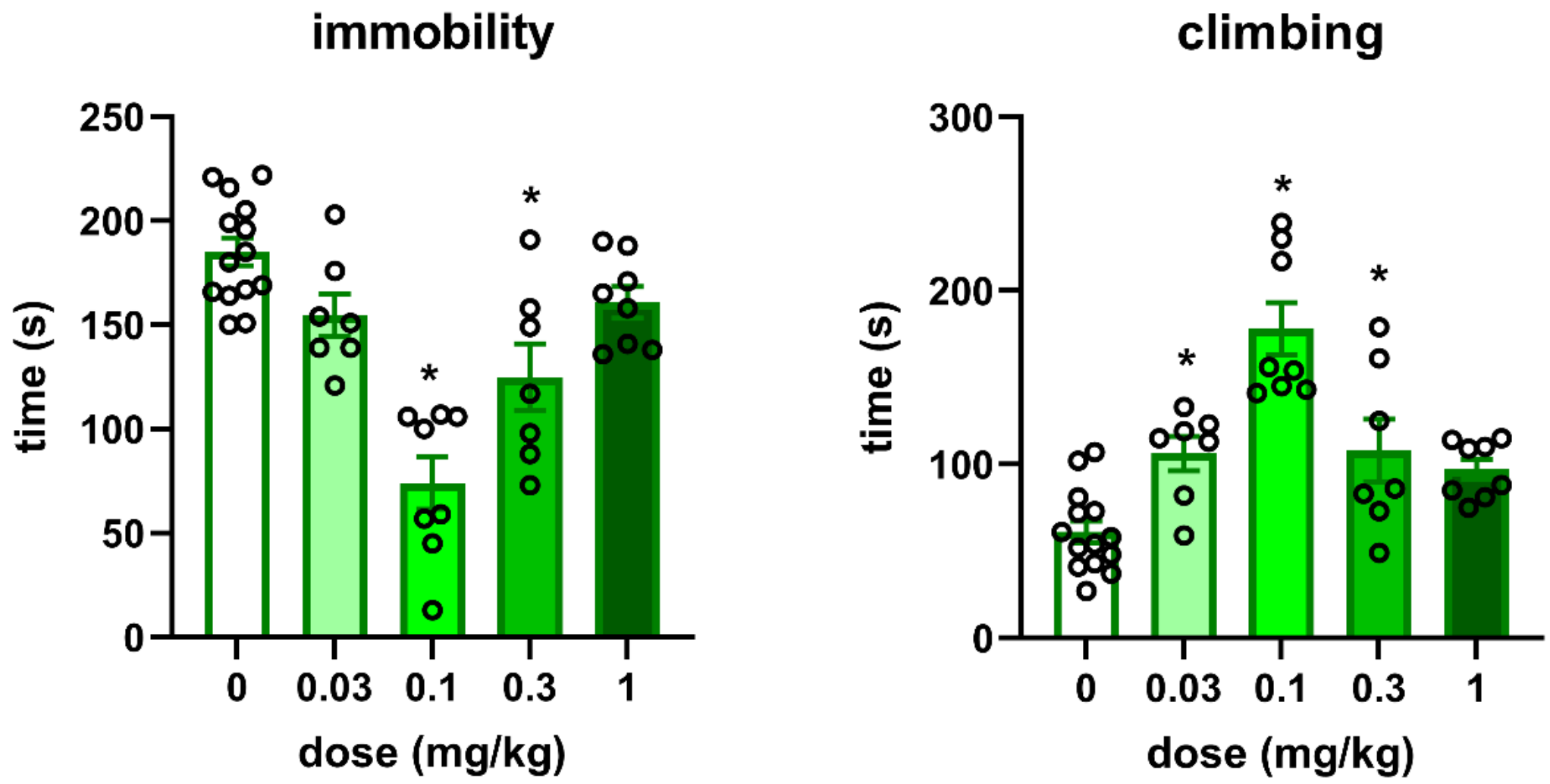

Supplemental Figure 11. The effects of PZKKN-94 on immobility and climbing time in FST rats. The data are shown as the mean ± SEM. N = 7-14 rats per group. Symbols: * $P < 0.05$, a significant decrease in immobility and/or increase in climbing compared with vehicle-treated control group.

Supplemental Table 1. Selectivity of PZKKN-94 at 10 μM. This table presents percent activity or inhibition of a specific control. "Interaction" is denoted as value > 70%.

Protein	Assay Mode	
	Agonistic	Antagonistic
5-hydroxytryptamine receptor 1B	105.3[a]	-0.2
5-hydroxytryptamine receptor 6	NT	100[b]
adenosine A2A receptor	-4.1	-2.0
alpha-1A adrenergic receptor	-20.9	50.1
alpha-2A adrenergic receptor	-16.3	6.4
beta-1 adrenergic receptor	0.4	86.2
beta-2 adrenergic receptor	0.5	89.9
vasopressin receptor 1A	-1.7	-2.4
cholecystokinin A Receptor	-1.1	1.9
cholinergic Receptor Muscarinic 1	2.6	77.1
cholinergic Receptor Muscarinic 2	-50.5	35.8
cholinergic Receptor Muscarinic 3	3.8	41.8
cannabinoid receptor 1	-22.5	-1.4
cannabinoid receptor 2	-65.1	0.9
dopamine receptor D1	0.8	-12.7
dopamine receptor D2 (short form)	-14.7	2.9
endothelin A receptor	-1.9	-5.5
histamine Receptor H1	1.9	37.8
histamine Receptor H2	0.5	-49.8
5-hydroxytryptamine receptor 1A	50.2	-0.9
5-hydroxytryptamine receptor 2A	-2.6	68.8
5-hydroxytryptamine receptor 2B	3.5	5.4
opioid receptor delta 1	-2.0	0.6
opioid receptor kappa 1	-17.3	2.8
opioid receptor. mu 1	-8.5	1.1
androgen receptor	-0.4	3.2
glucocorticoid receptor	-0.3	-13.7
	**Blocker**	**Opener**
dopamine active transporter	28.8	-
norepinephrine transporter	50.3	-
serotonin transporter	32.0	-
calcium channel. voltage-dependent. L type.	3.7	-
γ-Aminobutyric acid type A	10.3	-2.3
potassium voltage-gated channel subfamily H	61.9	-
5-Hydroxytryptamine Receptor 3A	96.8	6.6
sodium channel protein type 5 subunit alpha	72.5	-
	**Inhibitor**	
acetylcholinesterase	8.5	
cyclooxygenase 1	4.2	
cyclooxygenase 2	-26.4	
monoamine oxidase A	10.3	

phosphodiesterase 3A	-4.5
phosphodiesterase 4D2	-2.0
insulin receptor	-8.6
lymphocyte-specific protein tyrosine kinase	6.1
rho-associated protein kinase 1	2.7
vascular endothelial growth factor 2	33.9

[a] tested at Eurofins: EC_{50} (5-HT_{1B}R) equals 59 nM). [b] tested in-house: K_b (5-HT_6) for PZKKN-94 equals 1.2 nM

Supplemental Table 2. Functional potency (IC_{50} or EC_{50}) for PZKKN-94 against targets selected based on the selectivity screen (Supplemental Table 1).

Protein	Mode	IC_{50}/EC_{50} (μM)[a]
beta-1 adrenergic receptor	Antagonist	2.3 (1.4;3.3)
beta-2 adrenergic receptor	Antagonist	2.3 (2.0;2.5)
cholinergic receptor muscarinic 1	Antagonist	2.3 (1.5;3.0)
5-hydroxytryptamine receptor 2A	Antagonist	5.9 (6.5; 5.4)
5-hydroxytryptamine receptor 3A	Blocker	1.6 (1.6; 1.6)
sodium channel protein type 5 subunit alpha	Blocker	> 10 (n.d.)

[a] Results represent the mean (range) (n = 2); n.d. - not determined

Supplemental Table 3. Membrane permeability of PZKKN-94 assessed in a Caco2 assay.

Compound		Caco2 Permeability (Direction=A2B) (pH 7.4/7.4)	Caco2 Permeability (Direction=B2A) (pH 7.4/7.4)	Ratio B2A / A2B
**Test item**	**Concentration (µM)**	**Mean Papp [a] (x 10^{-6} cms^{-1})**	**Mean Papp [a] (x 10^{-6} cms^{-1})**	
PZKKN-94	10	**22.4 (19.8; 25.1)**	**19.9 (19.9; 19.9)**	0.9
PZKKN-94 +verapamil	10	**28.0 (27.6; 28.4)**	**16.7 (16.4; 18.1)**	0.6
Colchicine	10	**0.8 (0.7; 1.0)**	**3.8 (3.2; 4.4)**	4.6
Labetalol	10	**10.5 (10.4; 10.6)**	**17.8 (16.9; 17.1)**	1.6
Propranolol	10	**37.4 (34.7;40.2)**	**23.1 (21.8; 24.4)**	0.6
Ranitidine	10	**0.5 (0.5; 0.5)**	**0.7 (0.6; 0.9)**	1.4

[a] Results represent the mean (range) (n = 2)

Supplemental Table 4. Genotoxicity assessment of PZKKN-94 in Ames test with presence or absence of S9 metabolic fraction.

**Without metabolic activation (-S9 mix) n=3**						
Strain	Vehicle/ Compound/ PC	Conc. Level (µg/plate)	Mean	Standard Deviation	Fold Increase	Range
TA98	DMSO	-	16.3	4.0	-	(12; 20)
	PZKKN-94	5	22.0	4.4	1.3	(17; 25)
		16	23.3	3.8	1.4	(19; 26)
		50	27.0	3.6	1.7	(24; 31)
		160	19.0	4.4	1.2	(16; 24)
		500	23.3	5.5	1.4	(17; 27)
		1600	23.7	6.4	1.4	(19; 31)
		5000	-	-	-	(n.d.)
	2NF	5	575.3	64.3	35.2	(526; 648)
TA100	DMSO	-	93.0	7.2	-	(85; 99)
	PZKKN-94	5	84.3	9.2	0.9	(79; 95)
		16	86.0	5.2	0.9	(80; 89)
		50	94.0	17.3	1.0	(84;114)
		160	97.3	2.5	1.0	(95; 100)
		500	88.3	4.7	0.9	(83; 92)
		1600	53.3	9.2	0.6	(48; 64)
		5000	-	-	-	- T, - T, - T (n.d.)
	NaN_3	2	602.3	34.1	6.5	(567; 635)
TA1535	DMSO	-	11.7	3.1	-	(9; 15)
	PZKKN-94	5	12.3	2.5	1.1	(10; 15)
		16	9.7	5.5	0.8	(4; 15)
		50	13.7	2.9	1.2	(12; 17)
		160	15.7	1.2	1.3	(15; 17)
		500	13.0	6.6	1.1	(6;19)
		1600	14.0	4.4	1.2	(11; 19)
		5000	-	-	-	(n.d.)
	NaN_3	2	526.3	25.4	45.1	(497; 542)
TA1537	DMSO	-	8.7	1.5	-	(7; 10)
	PZKKN-94	5	3.7	2.5	0.4	(1; 6)
		16	8.3	2.1	1.0	(6; 10)
		50	4.0	3.6	0.5	(0; 7)
		160	13.0	2.6	1.5	(10; 15)
		500	5.3	1.5	0.6	(4; 7)
		1600	3.7	2.3	0.4	(1; 5)
		5000	-	-	-	(n.d.)
	AAC	50	186.0	29.3	21.5	(163; 219)
TA102	DMSO	-	283.7	47.4	-	(229; 314)
	PZKKN-94	5	286.3	8.4	1.0	(281; 296)
		16	309.3	44.6	1.1	(276; 360)
		50	321.7	34.1	1.1	(286; 354)
		160	324.3	25.4	1.1	(296; 345)
		500	308.0	20.7	1.1	(285; 325)
		1600	-	-	-	(n.d.)
		5000	-	-	-	(n.d.)
	MMC	0.2	955.3	168.3	3.4	(762; 1069)

With metabolic activation (+S9 mix) n = 3						
Strain	Vehicle/ Compound/ PC	Conc. Level (µg/plate)	Mean	Standard Deviation	Fold Increase	Revertant Numbers Per Plate
TA98	DMSO	-	31.0	5.3	-	(25; 35)
	PZKKN-94	5	31.3	8.5	1.0	(25; 41)
		16	23.3	2.9	0.8	(20; 25)
		50	35.3	5.5	1.1	(30; 41)
		160	31.7	5.8	1.0	25; 35()
		500	37.3	6.4	1.2	(30; 41)
		1600	21.3	5.9	0.7	(17; 28)
		5000	-	-	-	(n.d.)
	B[a]P	10	143.0	12.2	4.6	(129; 151)
TA100	DMSO	-	97.3	4.6	-	(92; 100)
	PZKKN-94	5	94.3	19.3	1.0	(72; 106)
		16	97.0	13.0	1.0	(82; 105)
		50	95.7	10.2	1.0	(84; 103)
		160	89.3	12.9	0.9	(75; 100)
		500	96.7	8.5	1.0	(88; 105)
		1600	43.3	9.9	0.4	(32; 50)
		5000	-	-	-	(n.d.)
	AAN	5	1395.3	32.7	14.3	(1374; 1433)
TA1535	DMSO	-	14.0	2.6	-	(11;16)
	PZKKN-94	5	10.0	1.0	0.7	(9;11)
		16	14.3	4.0	1.0	(12; 19)
		50	17.3	6.4	1.2	(10; 21)
		160	14.3	3.1	1.0	(11; 17)
		500	14.3	2.1	1.0	(12; 16)
		1600	10.3	2.9	0.7	(7; 12)
		5000	-	-	-	(n.d.)
	AAN	5	326.3	28.4	23.3	(308; 359)
TA1537	DMSO	-	11.7	8.1	-	(7; 21)
	PZKKN-94	5	13.3	3.8	1.1	(9; 16)
		16	10.0	1.7	0.9	(9; 12)
		50	14.0	4.4	1.2	(9; 17)
		160	13.3	6.7	1.1	(9; 21)
		500	10.0	2.6	0.9	(7; 12)
		1600	2.3	3.2	0.2	(0; 6)
		5000	-	-	-	(n.d.)
	AAN	5	273.0	36.2	23.4	(235; 307)
TA102	DMSO	-	381.0	13.2	-	(371; 396)
	PZKKN-94	5	386.0	7.9	1.0	(377; 392)
		16	401.0	20.0	1.1	(379; 418)
		50	397.7	13.3	1.0	(389; 413)
		160	366.0	17.1	1.0	(348; 382)
		500	325.3	39.9	0.9	(297; 371)
		1600	-	-	-	(n.d.)
		5000	-	-	-	(n.d.)
	AAN	20	1412.7	133.9	3.7	(1260; 1510)

Abbreviations: PC – positive control; 2NF - 2-Nitrofluorene; NaN_3 - Sodium azide, AAC - 9-Aminoacridine; MMC - Mitomycin C; B[a]P - Benzo[a]pyrene; AAN - 2-Aminoanthracene; S - Slight thinning of background bacterial lawn; T - Toxic, no revertant colonies

Supplemental Table 5. Effect of compound PZKKN-94 on rat locomotor activity measured in the open field test.

Treatment (mg/kg)	N	Total distance (cm) during 5 min Mean ± SEM		
Vehicle	8	2761,4	±	119,9
PZKKN-94 (0.1)	6	2787,7	±	140,8
PZKKN-94 (0.3)	6	2542,8	±	54,9
		F(2,17) = 1,279, NS		

PZKKN-94 was injected *i.p*. 60 min before the test. Values represent the mean ± SEM during the 5-min test session compared to the vehicle-treated group (one-way ANOVA is followed by Bonferroni's post hoc test); (N = 6-8); NS not significant.

Supplemental Table 6. F and p values of both behavioral data presented in the results section.

Figure(s)	Effect	F (df, error)	P
4A PZKKN-94 in stepping test	treatment<br>time (repeated factor)<br>treatment x time interaction	F[3,28] = 5.206<br>F[1,28] = 36.895<br>F[3,28] = 5.675	P = 0.006<br>P < 0.0001<br>P = 0.004
4B PZKKN-94 + L-DOPA in stepping test	treatment<br>time (repeated factor)<br>treatment x time interaction	F[3,27] = 1.114<br>F[3,81] = 222.975<br>F[9,81] = 1.895	P = 0.361<br>P < 0.0001<br>P = 0.064
4C acute PZKKN-94 in vibrissae test	treatment	chi2 (32, df 1) = 0.067	P = 0.796
4D acute L-DOPA in vibrissae test	treatment	chi2 (9, df 1) = 2.667	P = 0.102
4D acute PZKKN-94 + L-DOPA in vibrissae test	treatment	chi2 (31, df 1) = 17.640	P < 0.0001
4D chronic PZKKN-94 + L-DOPA in vibrissae test	treatment	chi2 (31, df 1) = 31.000	P < 0.0001
4D chronic PZKKN-94 + L-DOPA OFF phase	treatment	chi2 (31, df 1) = 15.696	P < 0.0001
5A (PZKKN-94 in PCP NOR)	treatment	F(4,37) = 22.19	P < 0.001
5B (PZKKN-94, in scopolamine NOR)	week (repeated factor) x PZKKN-94 x scopolamine (between-subjects factors) interaction<br>PZKKN-94 x scopolamine interaction	F(1,32) = 0.019<br>F(1,32) = 91.278	P = 0.888<br>P < 0.001
5C (age differences in NOR)	age x ITI interaction	F(3,59) = 2.255	P = 0.09
5D (PZKKN-94 & Donepezil in NOR in young & aged)	age x treatment interaction<br>treatment	F(3,67) = 0.968<br>F(3,67) = 3.050	P = 0.412<br>P = 0.0344
6 (PZKKN-94 in ASST)	treatment x discrimination phase interaction	F[18,168] = 34.59	P< 0.001

Supp. 7 PZKKN-94 in haloperidol-induced catalepsy	treatment	F(2,33) = 3.802	P = 0.033
Supp. 8 PZKKN-94 + L-DOPA in 6-OHDA-produced catalepsy	treatment<br>time (repeated factor)<br>treatment x time interaction	F(3,27) = 0.892<br>F(3,81) = 57.624<br>F(9,81) = 1.311	P = 0.458<br>P < 0.0001<br>P = 0.244
Supp. 9 PZKKN-94 + L-DOPA in rotational behavior	treatment<br>time (repeated factor)<br>treatment x time interaction	F(3,27) = 0,158<br>F(2,54) = 17.017<br>F(6,54) = 1.364	P = 0.923<br>P < 0.0001<br>P = 0.246
Supp. 10 6-OHDA-induced deficits in<br>A stepping test<br>B vibrissae test<br>C catalepsy	lesion vs. pretest<br>lesion vs. pretest<br>lesion vs. pretest	t(31) = 23,044<br>Z = 4.937<br>Z = 4.860	P < 0.0001<br>P < 0.0001<br>P < 0.0001
Supp. 11 (PZKKN-94 in FST)	treatment<br>immobility time<br>climbing time	F(4,39) = 18.67<br>F(4,39) = 17.17	P < 0.0001<br>P < 0.0001

SI References

Cheng, H.C. (2001). The power issue: determination of K_B or K_i from IC_{50}. A closer look at the Cheng-Prusoff equation, the Schild plot and related power equations. *J. Pharmacol. Toxicol. Methods 46*: 61–71.

Cheng, Y., and Prusoff, W.H. (1973). Relationship between the inhibition constant (*K*1) and the concentration of inhibitor which causes 50 per cent inhibition (I_{50}) of an enzymatic reaction. *Biochem. Pharmacol. 22*: 3099–3108.

Detke, M.J., Rickels, M., and Lucki, I. (1995). Active behaviors in the rat forced swimming test differentially produced by serotonergic and noradrenergic antidepressants. *Psychopharmacology 121*: 66–72.

Drop, M., Canale, V., Chaumont-Dubel, S., Kurczab, R., Satała, G., Bantreil, X., et al. (2021). 2-Phenyl-*1H*-pyrrole-3-carboxamide as a new scaffold for developing 5-HT_6 receptor inverse agonists with cognition-enhancing activity. *ACS Chem. Neurosci. 12*: 1228–1240.

Grychowska, K., Satała, G., Kos, T., Partyka, A., Colacino, E., Chaumont-Dubel, S., et al. (2016). Novel *1H*-pyrrolo[3,2-*c*]quinoline based 5-HT_6 receptor antagonists with potential application for the treatment of cognitive disorders associated with Alzheimer's Disease. *ACS Chem. Neurosci. 7*: 972–983.

Hillegaart, V., Ahlenius, S., Magnusson, O., and Fowler, C.J. (1987). Repeated testing of rats markedly enhances the duration of effects induced by haloperidol on treadmill locomotion, catalepsy, and a conditioned avoidance response. *Pharmacol. Biochem. Behav. 27*: 159–164.

Kamiński, K., Grychowska, K., Canale, V., Bojarski, A.J., Satała, G., Lenda, T., et al. (2019). Isoindoline derivatives for the treatment of CNS diseases. PCT/IB2017/057247.

Konieczny, J., Czarnecka, A., Lenda, T., Kamińska, K., and Lorenc-Koci, E. (2014). Chronic L-DOPA treatment attenuates behavioral and biochemical deficits induced by unilateral lactacystin administration into the rat substantia nigra. *Behav. Brain Res. 261*: 79–88.

Konieczny, J., and Lenda, T. (2013). Contribution of the mGluR7 receptor to antiparkinsonian-like effects in rats: a behavioral study with the selective agonist AMN082. *Pharmacol. Rep. 65*: 1194–1203.

Kroeze, W.K., Sassano, M.F., Huang, X.-P., Lansu, K., McCorvy, J.D., Giguère, P.M., et al. (2015). PRESTO-Tango as an open-source resource for interrogation of the druggable human GPCRome. *Nat. Struct. Mol. Biol. 22*: 362–369.

Lenda, T., Ossowska, K., Berghauzen-Maciejewska, K., Matłoka, M., Pieczykolan, J., Wieczorek, M., et al. (2021). Antiparkinsonian-like effects of CPL500036, a novel selective inhibitor of phosphodiesterase 10A, in the unilateral rat model of Parkinson's disease. *Eur. J. Pharmacol. 910*: 174460.

Lewis, V., Bonniwell, E.M., Lanham, J.K., Ghaffari, A., Sheshbaradaran, H., Cao, A.B., et al. (2023). A non-hallucinogenic LSD analog with therapeutic potential for mood disorders. *Cell Rep. 42*: 112203.

Olsen, R.H.J., DiBerto, J.F., English, J.G., Glaudin, A.M., Krumm, B.E., Slocum, S.T., et al. (2020). TRUPATH, an open-source biosensor platform for interrogating the GPCR transducerome. *Nat. Chem. Biol. 16*: 841–849.

Popik, P., Khoo, S.Y.-S., Kuziak, A., Golebiowska, J., Potasiewicz, A., Hogendorf, A., et al. (2020).

Distinct cognitive and discriminative stimulus effects of ketamine enantiomers in rats. *Pharmacol. Biochem. Behav. 197*: 173011.

Potasiewicz, A., Golebiowska, J., Popik, P., and Nikiforuk, A. (2018). Procognitive effects of varenicline in the animal model of schizophrenia depend on α4β2- and α 7-nicotinic acetylcholine receptors. *J. Psychopharmacol. 33*: 269881118812097.

Potasiewicz, A., Krawczyk, M., Gzielo, K., Popik, P., and Nikiforuk, A. (2020). Positive allosteric modulators of alpha 7 nicotinic acetylcholine receptors enhance procognitive effects of conventional anti-Alzheimer drugs in scopolamine-treated rats. *Behav. Brain Res. 385*: 112547.

Rénéric, J.P., and Lucki, I. (1998). Antidepressant behavioral effects by dual inhibition of monoamine reuptake in the rat forced swimming test. *Psychopharmacology 136*: 190–197.

Zajdel, P., Grychowska, K., Mogilski, S., Kurczab, R., Satała, G., Bugno, R., et al. (2021). Structure-based design and optimization of FPPQ, a dual-acting 5-HT_3 and 5-HT_6 receptor antagonist with antipsychotic and procognitive properties. *J. Med. Chem. 64*: 13279–13298.

Zajdel, P., Kos, T., Marciniec, K., Satała, G., Canale, V., Kamiński, K., et al. (2018). Novel multi-target azinesulfonamides of cyclic amine derivatives as potential antipsychotics with pro-social and pro-cognitive effects. *Eur. J. Med. Chem. 145*: 790–804.
